# Copper-Catalyzed One-Pot Synthesis of Thiazolidin-2-imines

**DOI:** 10.1021/acs.joc.4c00394

**Published:** 2024-05-10

**Authors:** Leandros P. Zorba, Ioannis Stylianakis, Nikolaos Tsoureas, Antonios Kolocouris, Georgios C. Vougioukalakis

**Affiliations:** † Laboratory of Organic Chemistry, Department of Chemistry, National and Kapodistrian University of Athens, Panepistimioupolis, 15771 Athens, Greece; ‡ Laboratory of Medicinal Chemistry, Section of Pharmaceutical Chemistry, Department of Pharmacy, National and Kapodistrian University of Athens, Panepistimioupolis Zografou, 15771 Athens, Greece; § Laboratory of Inorganic Chemistry, Department of Chemistry, National and Kapodistrian University of Athens, Panepistimioupolis, 15771 Athens, Greece

## Abstract

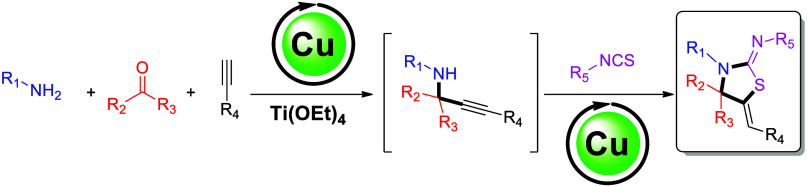

The synthesis of thiazolines, thiazolidines, and thiazolidinones has been extensively studied, due to their biological activity related to neurodegenerative diseases, such as Parkinson’s and Alzheimer’s, as well as their antiparasitic and antihypertensive properties. The closely related thiazolidin-2-imines have been studied less, and efficient strategies for synthesizing them, mainly based on the reaction of propargylamines with isothiocyanates, have been explored less. The use of one-pot approaches, providing modular, straightforward, and sustainable access to these compounds, has also received very little attention. Herein, we report a novel, one-pot, multicomponent, copper-catalyzed reaction among primary amines, ketones, terminal alkynes, and isothiocyanates, toward thiazolidin-2-imines bearing quaternary carbon centers on the five-membered ring, in good to excellent yields. Density functional theory calculations, combined with experimental mechanistic findings, suggest that the copper(I)-catalyzed reaction between the *in situ*-formed propargylamines and isothiocyanates proceeds with a lower energy barrier in the pathway leading to the S-cyclized product, compared to that of the N-cyclized one, toward the chemo- and regioselective formation of 5-exo-dig S-cyclized thiazolidin-2-imines.

## Introduction

**Figure 1:**
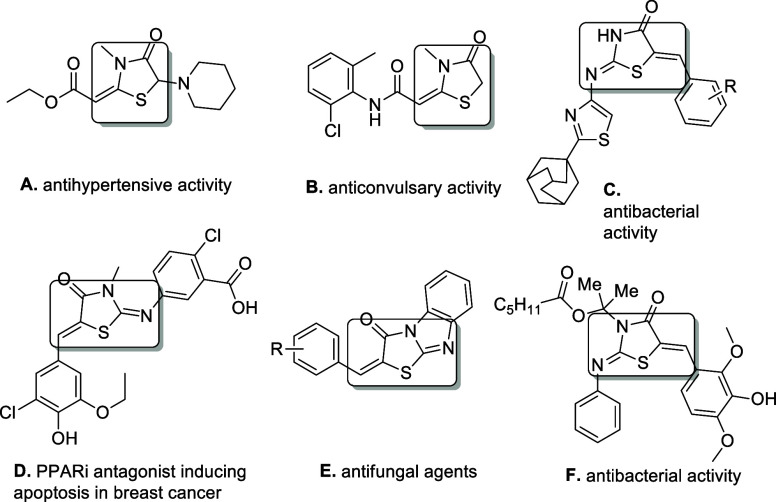
Selected examples of thiazolidine-based compounds with biological activity.

Nitrogen-, sulfur-, and oxygen-containing heterocycles are among the most intriguing biologically relevant classes of compounds in organic chemistry.^1^ Their privileged structures are omnipresent in a plethora of pharmaceuticals and natural products, as well as in numerous ligand scaffolds used in catalysis. The case of thiazolines, thiazolidinones, and thiazolidines is of particular interest, given that they have important biological applications against neurodegenerative diseases, such as Alzheimer’s and Parkinson’s, as well as anticonvulsant, anti-inflammatory, anticancer, antiviral, antihypertensive, and antiparasitic properties, among others.^2^ Examples include etozoline **A** (Figure 1A), which exhibits antihypertensive activity,^3^ ralitoline **B** (Figure 1B), which exhibits anticonvulsant properties,^3^ 4-adamantyl-2-thiazolylimino-5-arylidene-4-thiazolidinones **C** (Figure 1C), which exhibit antibacterial activity,^4^ 3-thiazolidine-benzoic acid derivative **D** (Figure 1D), which exhibits anticancer activity,^2e,5^ specifically acting as a PPARγ antagonist, benzimidazo-thiazolinone-ylidine derivatives **E** (Figure 1E), which exhibit antifungal activity,^6^ and thiazolidine-ylidene derivative **F** (Figure 1F), which exhibits antibacterial activity against *Salmonella enterica*.^4^ When the carbonyl group in the five-membered thiazolidinone core of compound **C**, **D**, or **F** is replaced with carbon-based substituents, compounds with antiproliferative activity against breast cancer are obtained.^7^

**Scheme 1:**
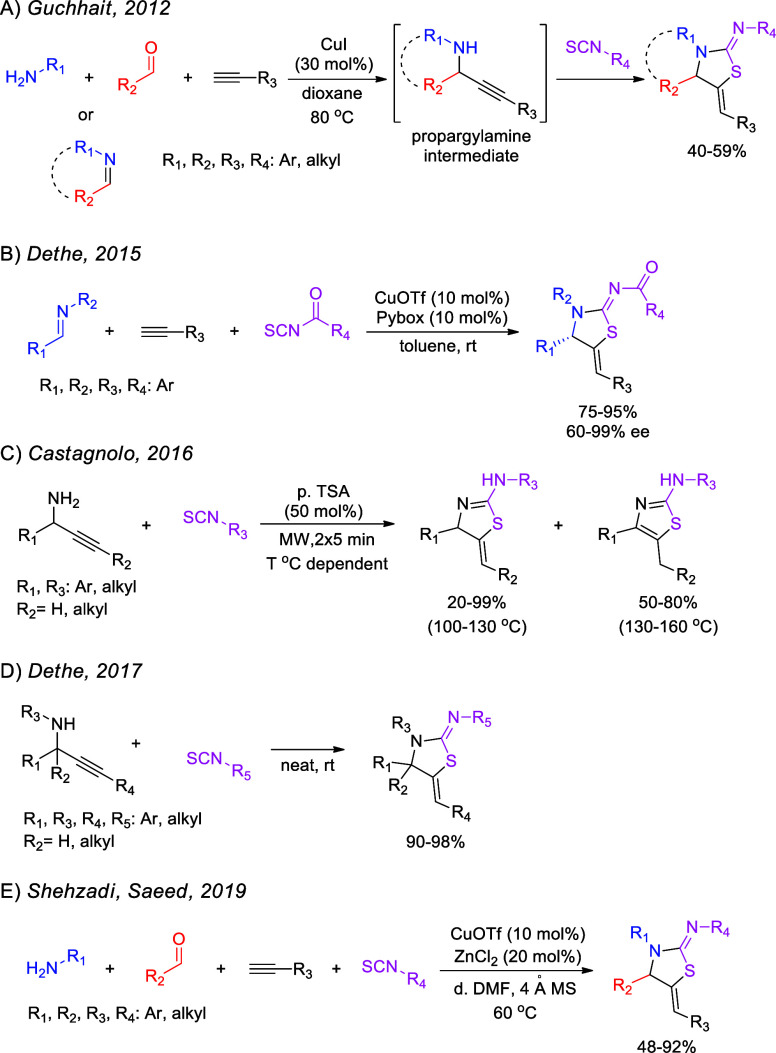
Overview of the Reactions of Isothiocyanates with *In Situ*-Generated Propargylamines from Primary Amines, Terminal Alkynes, and Carbonyl Compounds, or by Using Preformed Propargylamines

Synthetic approaches toward these types of thiazolidines are relatively common in the literature, usually employing isothiocyanate derivatives as reaction partners;^2a,8^ however, thiazolidin-2-imines (or thiazolidin-2-ylideneamines) in which the α-carbon situated between the nitrogen atom of the five-membered ring and the exocyclic C=C bond is attached to substituents other than oxygen (carbonyl) or hydrogen (methylenic) have not received an analogous amount of attention. These scaffolds are usually accessed by reacting propargylamines with isothiocyanate derivatives; in most cases, no catalyst is required.^9^ In this regard, the (primary or secondary) nitrogen atom of the propargylamine substrate nucleophilically attacks the carbon center of the isothiocyanate moiety, toward the corresponding propargyl thiourea, which, via a 5-exo-dig S-cyclization or a 5-exo-dig N-cyclization, furnishes the corresponding thiazolidin-2-imines or imidazol-2-thiones, respectively.^9b^ Originally observed by Easton and co-workers in 1964, the reaction of a series of propargylamines with isothiocyanates does not stop with propargyl thioureas, as they are cyclized to thiazolidin-2-imines (via a 5-exo-dig S-cyclization).^9^ Along these lines, several transformations toward thiazolidin-2-imines have been reported in the literature. Guchhait and co-workers have reported an efficient and sustainable protocol for the synthesis of thiazolidin-2-imines via a one-pot, two-step procedure, employing as starting materials primary amines and aldehydes, or preformed imines, along with terminal alkynes and isothiocyanate derivatives, in moderate yields (Scheme 1A).^10^ This was the first reported protocol for the synthesis of thiazolidin-2-imines via a one-pot, multicomponent approach, without isolating the required propargylamine intermediates. Note that multicomponent reactions are highly practical tools for synthesizing complex compounds in a one-pot manner, reducing the number of synthetic steps, experimental time, effort, and cost, compared to those of traditional multistep procedures.^11^ Later, the group of Dethe reported an elegant, one-pot multicomponent process toward chiral thiazolidin-2-imines (Scheme 1B), employing preformed imines, terminal alkynes, and isothiocyanates as starting materials, copper(I) triflate as the catalyst, and chiral pyridine bisoxazoline (pybox) as the ligand.^12^ The enantioselectivity of this process relies on the copper(I)/chiral pybox-mediated asymmetric insertion of the terminal alkyne into the imine moiety, followed by a 5-exo-dig S-cyclization, toward the desired products in good to high yields and enantiomeric excesses. In another related work, Castagnolo and co-workers reported an efficient, microwave-irradiation mediated reaction of tertiary propargyl α-primary amines and isothiocyanates (Scheme 1C), employing *p*-toluenesulfonic acid (*p*-TSA) as the catalyst, providing moderate to very good yields of the corresponding 2-aminothiazoles (structurally resembling thiazolidin-2-imines, when the amine functionality of propargylamines is primary), as well as 2-amino-4-methylenethiazolines, with an increase in the temperature of the reaction.^13^ Moreover, Dethe and co-workers have reported the reaction of tertiary and, to a lesser extent, quaternary propargylamines (that is, having quaternary carbon centers adjacent to the nitrogen and the alkyne group), either by mixing the two reactants under neat conditions or by using toluene or chloroform as the solvent (Scheme 1D).^14^

Similarly, Lovely and co-workers have reported the reaction of propargylamines having secondary or tertiary carbon centers with isothiocyanates, including propargylamines bearing terminal alkynes, instead of the previous examples, where propargylamines feature internal alkynes.^15^ In that work, the nucleophilic attack of the propargylamine on the isothiocyanate was reported to be relatively quick, in relation to the 5-exo-dig S-cyclization, which can be expedited in the presence of silica gel.^15^ Another multicomponent transformation toward thiazolidin-2-imines was reported by Shehzadi, Saeed, and co-workers, who claim to have accomplished a one-pot, four-component reaction, by introducing all reagents simultaneously (Scheme 1E).^16a^ This comprises the first report toward the synthesis of thiazolidin-2-imines in a one-pot, one-step manner. The resulting thiazolidine compounds were also evaluated as acetylcholinesterase inhibitors, providing promising results. Finally, Nikoofar and co-workers have reported a similar approach, using magnetized nano Fe_3_O_4_-SiO_2_@Glu-Cu(II) as the catalyst.^16b^

To the best of our knowledge, the synthesis of thiazolidin-2-imines via a one-pot, multicomponent strategy, from readily available primary amines, ketones, terminal alkynes, and isothiocyanates, without the necessity of isolating the corresponding α-secondary propargylamines bearing quaternary carbon centers adjacent to the nitrogen atom and the alkyne group, has not been reported thus far. Upon adoption of a multicomponent strategy, the overall synthetic steps required to furnish these products are minimized, therefore promoting atom economy, reduction of waste, and reduction of the overall time and cost required for the entire synthetic endeavor.^9b,17^ Our continuous interest in the design and development of catalytic protocols promoting atom economy, use of low-cost and nontoxic metal sources, in low catalyst loadings, toward invaluable and biologically relevant compounds, in an efficient and simple synthetic manner,^18^ prompted us to develop an efficient and user-friendly protocol for the synthesis of these thiazolidin-2-imines, via a one-pot sequence, employing commercially available starting materials, as well as low-cost and low-toxicity transition state metal catalysts.

## Results and Discussion

We initially investigated the one-pot, four-component strategy employing benzylamine, cyclohexanone, phenylacetylene, and phenyl isothiocyanate as substrates (Table 1). On the basis of our experience in the field of KA^2^ coupling (ketone–amine–alkyne reaction), we chose CuCl_2_ as the catalyst, which was added to an equimolar mixture of the reactants under neat conditions or with toluene as the solvent in the presence of molecular sieves. Molecular sieves were used to trap water, which is the byproduct of the first step of the target reaction (Table 1). However, no **6a** was detected under these conditions, nor was corresponding intermediate **5a** (Table 1, entry 1). Alternative drying agents were also used, such as Ti(OEt)_4_ and MgSO_4_, again not leading to the desired product (Table 1, entries 2–4). The use of DABCO, acting as a base, also did not lead to the target compound (Table 1, entry 5). Instead, thiourea byproduct **7a** was detected in all cases, formed by the nucleophilic attack of benzylamine to phenyl isothiocyanate. We then tried to reproduce the results of the published work, in which aldehydes, instead of ketones, react with primary amines, terminal alkynes, and isothiocyanate derivatives in a one-pot fashion (Scheme 1E).^16a^ As mentioned above, Cu(I) and Zn(II) salts were employed as the catalytic system in that work. Accordingly, several reaction conditions were tested, using Cu(OTf)_2_/ZnCl_2_ (Table 1, entries 6 and 7), Cu(OTf)_2_/ZnBr_2_ (Table 1, entry 8), or CuBr/Zn(OTf)_2_ (Table 1, entry 9), at 80 or 110 °C in DMF, using cyclohexanone as the carbonyl reagent. No desired product was formed in any of these reactions, in some cases leading to thiourea **7a**, among other byproducts. We also tested the exact reported catalytic protocol and conditions, using benzaldehyde, butanal, or pivalaldehyde at 60 °C in DMF (Table 1, entries 10–13).^16a^ No product was observed once again, in addition to the corresponding thiourea byproduct. Although these findings contradict the published work (Scheme 1E),^16a^ we were not entirely surprised. In specific, it is well-known that isothiocyanates are very reactive electrophiles, readily reacting with nitrogen nucleophiles, even in the absence of a base;^1e,6a,9b,19^ the outcome of this nucleophilic attack is a thiourea. On the contrary, amines react with carbonyl groups with respect to the formation of imines, a process that can be reversible, depending on the reaction conditions and the stability of the imine. Taking all of these factors into consideration, we reasoned that in a reaction mixture containing a primary amine along with carbonyl- and isothiocyanate-bearing compounds, the amine will nucleophilically attack the isothiocyanate group first. This is exactly what we observed experimentally (Table 1) in contrast to protocols reporting the inverse reactivity pattern.^16^

**Table 1:**
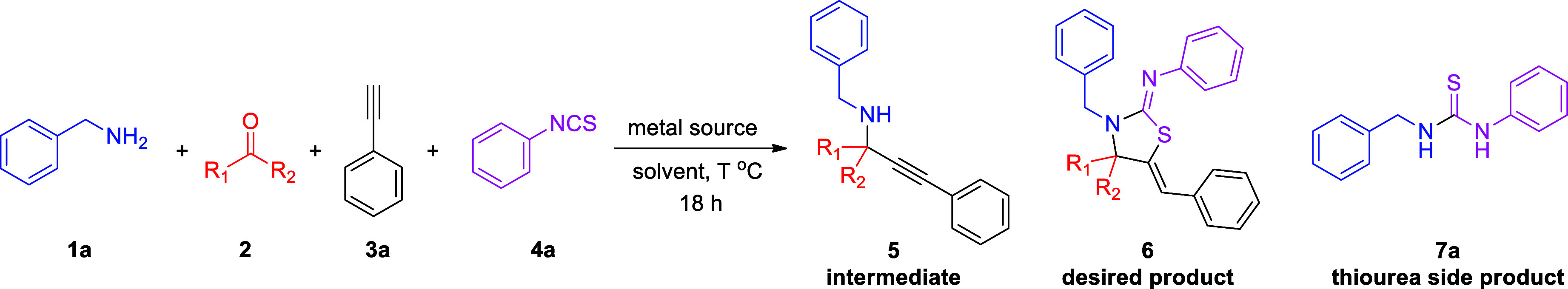
Experiments Targeting the One-Pot, Four-Component Reaction toward Products **6**^a^

entry	substrate	catalyst (mol %)	additive (equiv)	*T* (°C)	solvent	yield of **6** (%)
1	cyclohexanone	CuCl_2_ (5)	MS	110	–	–
2	cyclohexanone	CuCl_2_ (10)	Ti(OEt)_4_ (1)	110	–	–
3	cyclohexanone	CuCl_2_ (5)	MgSO_4_ (1)	110	toluene	–
4	cyclohexanone	CuCl_2_ (5)	MgSO_4_ (1)	110	–	–
5	cyclohexanone	CuCl_2_ (5)	DABCO (0.5)	110	–	–
			MgSO_4_ (1)			
6	cyclohexanone	Cu(OTf)_2_ (10)/ZnCl_2_ (20)	MS	80	DMF	–
7	cyclohexanone	Cu(OTf)_2_ (10)/ZnCl_2_ (20)	MS	110	DMF	–
8	cyclohexanone	Cu(OTf)_2_ (10)/ZnBr_2_ (20)	MS	80	DMF	–
9	cyclohexanone	CuBr (10)/Zn(OTf)_2_ (20)	MS	110	DMF	–
10	cyclohexanone	CuOTf (10)/ZnCl_2_ (20)	MS	60	DMF	–
11	benzaldehyde	CuOTf (10)/ZnCl_2_ (20)	MS	60	DMF	–
12^b^	butanal	CuOTf (10)/ZnCl_2_ (20)	MS	60	DMF	–
13^b^	pivalaldehyde	CuOTf (10)/ZnCl_2_ (20)	MS	60	DMF	–

a All reactions were carried out in a Schlenk J.-Young tube, under an an Ar atmosphere. The quantities of all starting materials were 1 equiv, unless otherwise mentioned.

b With 2 equiv of benzylamine.

Using CuCl_2_ as a catalyst, heating the mixture of benzylamine, cyclohexanone, and phenylacetylene at 110 °C, followed by decreasing the temperature to 35 °C and adding phenyl isothiocyanate, gave 42% of the desired product (Table 2, entry 1). Upon the introduction of toluene as the solvent at the second step, the yield of **6a** decreased to 27% (Table 2, entry 2), but increasing the second step’s temperature to 110 °C increased the yield of the desired product (Table 2, entry 3). Interestingly, using 2-octanone under the conditions of entry 3, to examine whether more challenging ketones are reactive under these conditions, provided the corresponding product in 39% isolated yield (Table 2, entry 4). By repeating the same reaction without adding phenyl isothiocyanate at the second step, we obtained a 35% isolated yield of the corresponding propargylamine, suggesting that the challenging step of the reaction is the first (Table 2, entry 5). The utilization of Ti(OEt)_4_ in the absence of a solvent increased the yield of the reaction to 57% (isolated yield, Table 2, entry 6). The use of Lewis base DABCO or DBU hindered the reaction substantially (Table 2, entries 7–10).

**Table 2:**
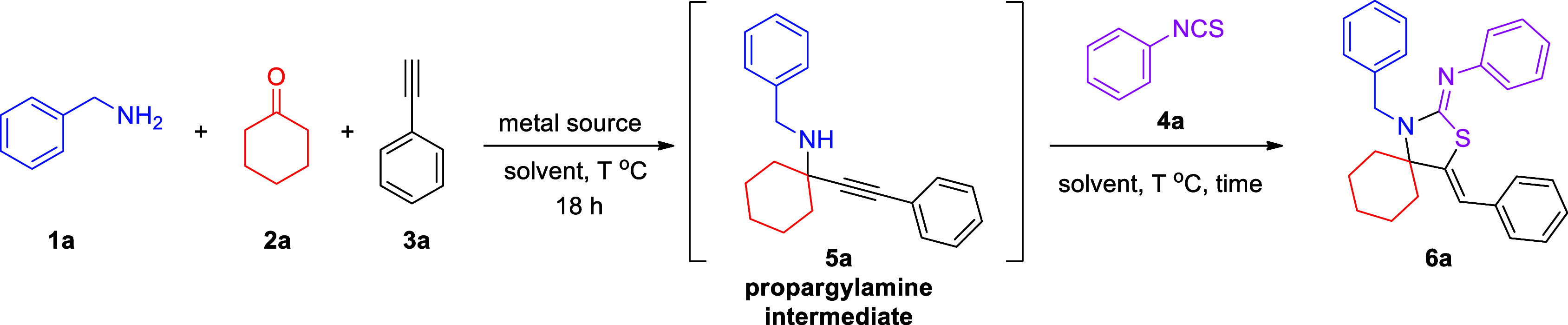
Optimization of the Sequential One-Pot Reaction^a^

entry	catalyst (mol %)	additive (equiv)	*T* (°C)	solvent (first step/second step)^b^	results^c^
1	CuCl_2_ (5)	–	110/35	–	42%
2	CuCl_2_ (5)	–	110/35	–/toluene	27%
3	CuCl_2_ (5)	–	110/110	–/toluene	45%
4^d^	CuCl_2_ (5)	–	110/110	–/toluene	45% (39%)
5^d^,^e^	CuCl_2_ (5)	–	110	–	44% (35%)
6	CuCl_2_ (5)	Ti(OEt)_4_ (0.5)	110/35	–	63% (57%)
7	CuCl_2_ (5)	DABCO (1)	110/35	–/toluene	17%
8	CuCl_2_ (5)	DBU (1)	110/35	–/toluene	9%
9	CuCl_2_ (5)	DABCO (0.5)	110/35	–/toluene	16%
10	CuCl_2_ (5)	DABCO (0.5)	110/0	–/toluene	16%
11	CuCl_2_ (5)	Ti(OEt)_4_ (1)	110/35	–	41% (35%)
12	CuCl_2_ (5)	Ti(OEt)_4_ (0.5)	110/110	toluene	71% (67%)
13	CuCl_2_ (5)	Ti(OEt)_4_ (0.5)	110/rt	toluene	73% (68%)
14	CuCl (5)	Ti(OEt)_4_ (0.5)	110/rt	toluene	40%
15^e^	CuCl_2_ (5)	–	110	–	17%
	Cu(OTf)_2_ (5)				
16^e^	CuCl_2_ (5)	–	110	–	13%
	InCl_3_ (5)				

a All reactions were carried out in a Schlenk J.-Young tube, under an an Ar atmosphere.

b Solvent utilized and the step of the transformation in which the solvent was added.

c Percentages show the yield based on ^1^H NMR analysis, while isolated yields are shown in parentheses.

d 2-Octanone used as the ketone substrate.

e Reaction halted at the first step.

In the reactions presented above, the corresponding imidazole-2-thione was also observed as a byproduct, under the influence of a base, formed by a 5-exo-dig N-cyclization, albeit in low yield; this observation is in line with the related work of Dethe.^20^ We then more carefully examined the effect of Ti(OEt)_4_ on the outcome of the first step. Ti(OEt)_4_ is well established to activate carbonyl groups, while also acting as a drying agent.^17,18d,21^ In this regard, the conditions of entry 6 of Table 2 were employed, using 1 equiv of Ti(OEt)_4_, in the absence of a solvent, leading to **6a** in 35% isolated yield, after chromatographic purification (Table 2, entry 11). By comparing the results of entries 6 and 11 of Table 2, we deduced that the increase in the amount of Ti(OEt)_4_ has a negative impact on the performance of the reaction. Upon introduction of toluene as the solvent, under the conditions of entry 11 of Table 2, and with an increase in the temperature of the second step to 110 °C, we obtained a 67% isolated yield, after chromatographic purification, for **6a** (Table 2, entry 12). Also of note is the fact that in the absence of a solvent the reaction mixture was very viscous, making stirring almost impossible. Carrying out the second step of the reaction at room temperature led to a 68% isolated yield (Table 2, entry 13). Replacing CuCl_2_ with CuCl as the catalyst led to a 40% yield for **6a** (Table 2, entry 14). We also tried to improve the outcome of the first step by employing two Lewis acids, instead of one, by concomitantly using Cu(OTf)_2_ (Table 2, entry 15) or InCl_3_ (Table 2, entry 16), but with poor results.

**Scheme 2:**
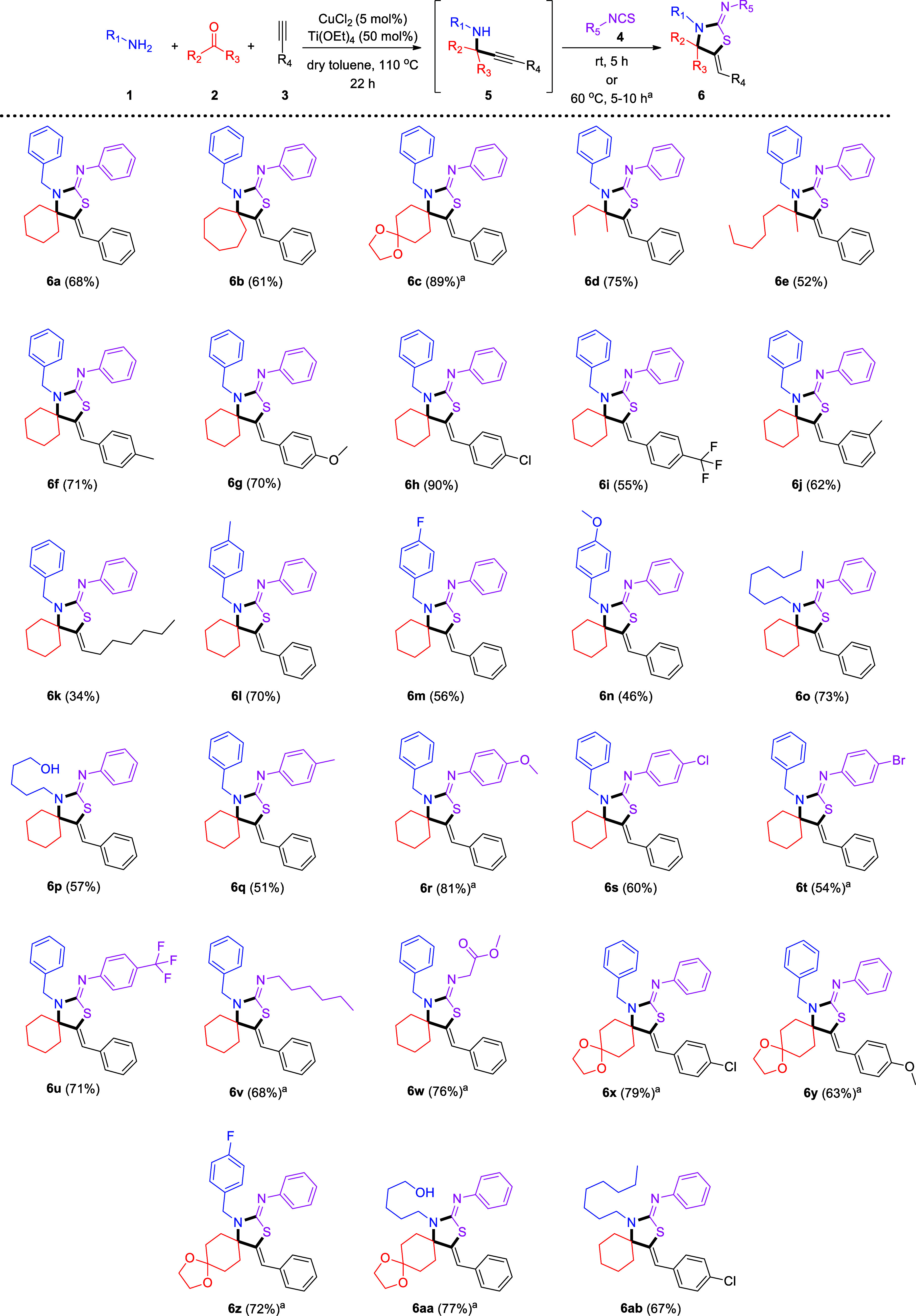
Reaction of a Variety of Substrates in the One-Pot Transformation Reported Herein The second step was left at room temperature or heated at 60 °C for 5–10 h. All reactions were carried out using equimolar amounts of amine (0.4 mmol), ketone (0.4 mmol), alkyne (0.4 mmol), and isothiocyanate (0.4 mmol), as well as CuCl_2_ (5 mol %) and Ti(OEt)_4_ (50 mol %), in toluene (50–500 μL).

With the optimized reaction conditions established, we continued with the study of the reactivity of a variety of substrates. Increasing the ring size of the carbonyl compound to cycloheptyl led to a very satisfactory 61% yield of **6b** (Scheme 2), whereas going down to cyclopentyl gave poor reactivity toward the corresponding product, which was anticipated, considering the relative reactivity of cyclohexanone versus cyclopentanone in the KA^2^ reaction. Using 1,4-dioxaspiro[4.5]decan-8-one led to product **6c** in an excellent 89% isolated yield, while 2-pentanone and 2-octanone were also effectively incorporated into our protocol, leading to **6d** and **6e** in 75% and 52% yields, respectively (Scheme 2). These results were also anticipated, given the known moderate KA^2^ reactivity of linear ketones. More “exotic” ketones, such as 2-chloro-3-butanone, 1-hydroxy-3-butanone, tropinone, and cyclododecanone, proved to be challenging substrates, providing poor results. The first two can clearly not be integrated into the reaction, due to their unique structural features, involving the presence of a chloride group and a hydroxyl group near the ketone group, respectively. Interestingly, although the propargylamine intermediate deriving from the use of cyclododecanone was identified in the ^1^H nuclear magnetic resonance (NMR) spectrum of the crude mixture, no reaction with phenyl isothiocyanate, followed by intramolecular cyclization toward the corresponding thiazolidin-2-imine, was detected.

We then studied the reactivity of a variety of alkynes, having an aryl or alkyl substitution. Incorporating electron-donating groups at the *para* position of phenylacetylene, such as a methyl or a methoxy, led to a small increase in the yield of the final products, **6f** or **6g** (71% or 70%, respectively) (Scheme 2). The presence of a chloride at the *para* position significantly increased the yield to 90% (**6h**), whereas stronger electron-withdrawing groups, such as -CF_3_, at the *para* position made the reaction more challenging, providing a 55% isolated yield of **6i** (Scheme 2). Introducing a methyl substituent at the *meta* position did not substantially change the reaction outcome, leading to a 62% isolated yield of **6j** (Scheme 2). This trend of reactivity was expected, given that electron rich aryl alkynes are more nucleophilic than electron poor aryl alkynes; therefore, a more successful nucleophilic attack of the *in situ*-generated copper acetylide on the electrophilic ketiminium is anticipated at the rate-determining step for the formation of intermediate propargylamine **5** [first step of the overall transformation (KA^2^)]. Interestingly, introducing a bromide at the *ortho* position of the phenyl ring of phenylacetylene led to a very low reactivity, and the product could not be fully purified. Employing alkyl-substituted terminal alkynes, as in the case toward product **6k**, significantly reduced the efficiency of the reaction, leading to a 34% isolated yield (Scheme 2).

With regard to the reactivity of amines, substitution at the *para* position of the phenyl ring of benzylamine with a methyl group gave a 70% isolated yield of **6l** (Scheme 2). On the contrary, *p*-F- and *p*-OMe-substituted benzylamines afforded products **6m** and **6n** in 56% and 46% yields, respectively (Scheme 2). This observation is in line with the findings of Larsen and co-workers for their KA^2^ protocol catalyzed by the same catalyst (CuCl_2_).^22^ A representative alkyl-substituted amine was also efficiently integrated into our protocol, affording **6o** in 73% isolated yield (Scheme 2). Interestingly, alkyl amines bearing a free hydroxyl group are also amenable to our protocol, for example, affording **6p** in 57% yield (Scheme 2). On the contrary, no product was obtained when *p*-OH benzylamine or aniline was used as the substrate. The introduction of more sterically demanding primary amines, such as (*S*)-(−)-1-phenylethylenamine or cyclohexylamine, does not lead to the formation of the corresponding products, because of the inefficient KA^2^ reaction. In other words, the progress of the first step of the overall transformation is negatively affected by stereochemical hindrance near the nucleophilic amino group.

Finally, we studied a variety of isothiocyanate analogues, with regard to the substituents on the aromatic ring of phenyl isothiocyanate, also probing alkyl isothiocyanates. Incorporation of a methyl group at the *para* position of phenyl isothiocyanate led to a decrease in the yield of product **6q** to 51% (Scheme 2). Utilization of a *p*-methoxy substituent increased the yield to 81% (**6r**, Scheme 2). Halogen (-Cl or -Br) or even stronger electron-withdrawing moieties, such as -CF_3_, at the *para* position resulted in good to very good yields for products **6s**–**6u** (60%, 54%, and 71%, respectively) (Scheme 2). An alkyl-substituted isothiocyanate was also efficiently incorporated, leading to **6v** in 68% isolated yield (Scheme 2). Our protocol also tolerates the presence of an ester moiety at the α-position to the isothiocyanate group, leading to **6w** in 76% yield (Scheme 2). This fact increases the usefulness of the transformation in late-stage functionalization and toward the synthesis of thiazolidin-2-imine derivatives with synthetically and biologically relevant moieties. More interesting functional moieties were attached to the five-membered ring (Scheme 2). The use of 1,4-dioxaspiro[4.5]decan-8-one along with *p*-chloro phenylacetylene led to **6x** in 79% yield (Scheme 2), whereas combining *p*-methoxy phenylacetylene with the same ketone led to **6y** in 63% yield (Scheme 2). Employing the same modified cyclohexanone along with *p*-fluoro benzylamine or 5-amino-pentan-1-ol afforded **6z** or **6aa** in 72% or 77% isolated yield, respectively. 1-Octylamine was also probed, along with *p*-chloro phenylacetylene, leading to **6ab** in 67% yield (Scheme 2). Moreover, to highlight the synthetic applicability of our protocol, a 4 mmol, gram-scale reaction was set up for the synthesis of **6a**, which was thus produced in 70% yield.

**Scheme 3:**
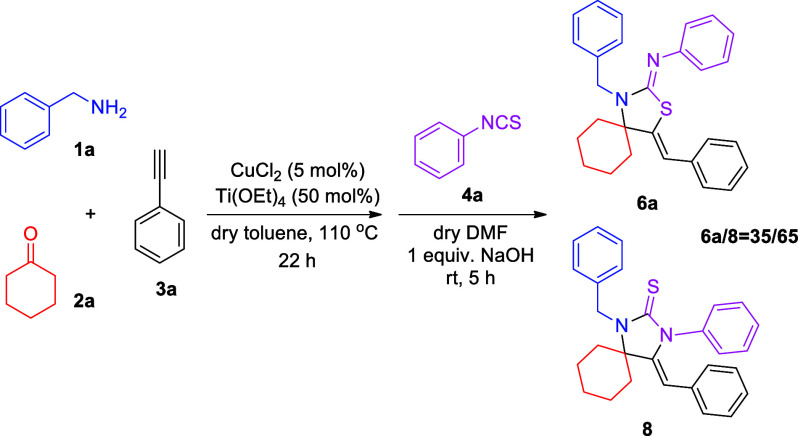
Chemoselectivity Obtained Using a Strong Base in the Second Step of the Overall Transformation

On the basis of our findings during the optimization experiments (Table 2), we were interested in probing the possibility of altering the chemoselectivity of the reaction, toward a 5-exo-dig N-cyclization reaction mode. This has been achieved by Dethe and co-workers, upon reacting preformed, isolated propargylamines with isothiocyanates in the presence of a strong base, i.e., NaOH.^20^ On this basis, we reacted benzylamine, cyclohexanone, and phenylacetylene, using CuCl_2_ as a catalyst in dry toluene, followed by the addition of phenyl isothiocyanate, dry DMF, and 1 equiv of NaOH (Scheme 3). This protocol led to the formation of a mixture of 5-exo-dig N-cyclization- and S-cyclization-derived products **8** and **6a**, respectively, in a 65/35 ratio; however, the reaction also concomitantly generated several byproducts. Increasing the reaction time of the second step, or the amount of NaOH to 2 equiv, did not improve the outcome of the reaction. Moreover, employing stronger bases, such as KOH or *t*-BuOK, resulted in a similar or reduced amount of **8** (64/36 or 40/60 for the **8**/**6a** ratio, respectively). The role of a strong base in directing the selectivity toward N-cyclization is the generation of a negative charge at the nitrogen atom of the thiourea intermediate, after the nitrogen atom of the propargylamine intermediate has nucleophilically attacked the carbon of the isothiocyanate moiety. This negative charge is mainly located at the nitrogen, thus leading to the 5-exo-dig N-cyclization, instead of the cyclization via the sulfur atom.

**Figure 2:**
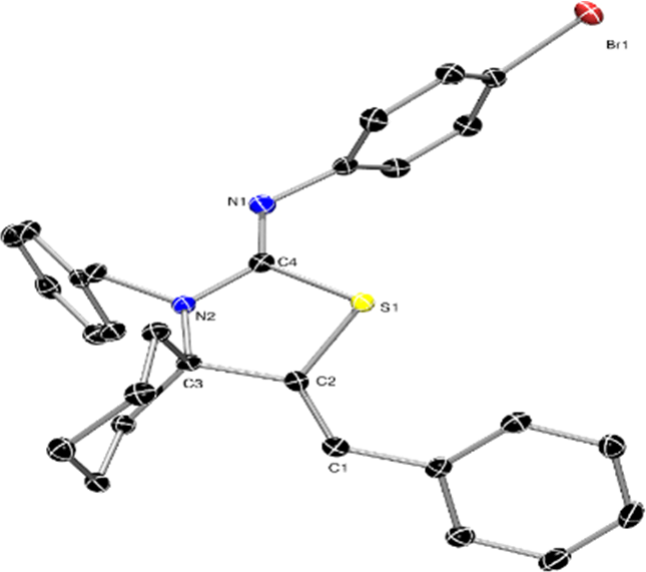
ORTEP-3 diagram of the molecular structure of compound **6t** (CCDC 2322776) displaying 50% ADP. Hydrogen atoms have been omitted for clarity. Selected bond lengths (angstroms) and angles (degrees): S1–C4, 1.7804(17); S1–C2, 1.7860(17); C1–C2, 1.338(2); C2–C3, 1.528(2); N2–C3, 1.494(2); N2–C4, 1.367(2); C4–N1, 1.271(2); Br1–C_*ipso*_, 1.9018(17); C2–S1–C4, 90.90(9); S1–C2–C3, 109.03(12); C2–C3–N2, 102.47(15); N2–C4–S1, 111.12(12); N2–C4–N1, 123.77(14); S1–C4–N1, 125.10(15).

In the case of **6t**, the S-cyclization was also structurally confirmed by single-crystal XRD characterization, showing that the thiazolidin-2-imine is indeed formed. This analysis further confirmed the *Z* conformation of the C=C bond (i.e., C1=C2 in Figure 2). Compound **6t** is isostructural with its *p*-chloro congener reported by Dethe and co-workers,^14^ with bond lengths and angles otherwise unremarkable and warranting no further discussion.

**Scheme 4:**
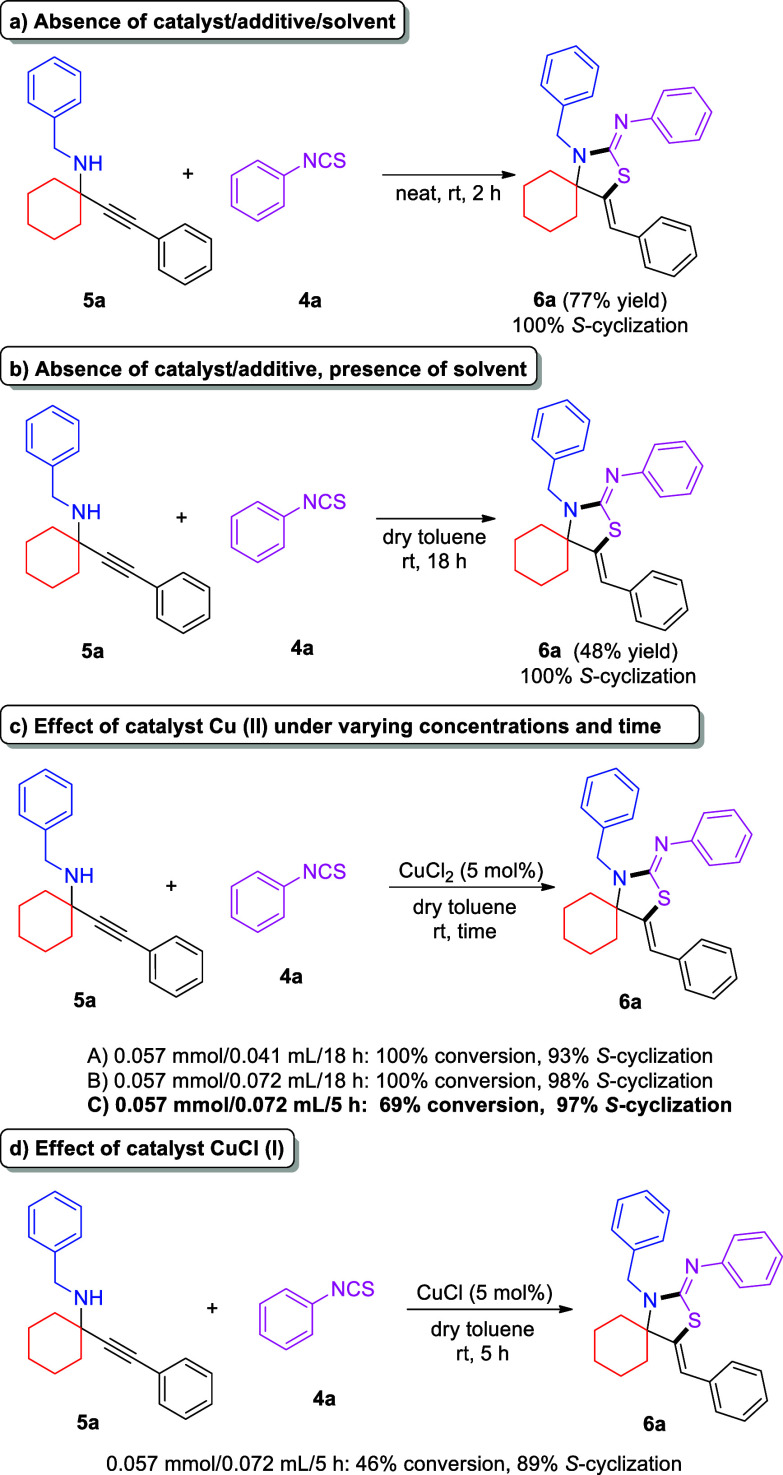
Control Experiments for the Second Step of the Reaction

As was deduced during the optimization experiments, as well as during the substrate reactivity studies, propargylamine is the key intermediate in the formation of thiazolidin-2-imine products **6**. This was proven beyond any doubt when propargylamine **5a** was isolated and then reacted with phenyl isothiocyanate under neat conditions, providing a 77% yield (Scheme 4a). We were particularly interested in studying the second step of the overall transformation, which involves the nucleophilic attack of the propargylamine on the isothiocyanate, followed by an intramolecular cyclization. This cyclization step has not been thoroughly studied thus far. To simulate the overall transformation’s reaction conditions, during cyclization, we added the appropriate amount of dry toluene, which resulted in a decrease in the reaction yield to 48% after 18 h, most probably due to the diluted conditions (Scheme 4b). Our overall transformation also requires the presence of CuCl_2_ to catalyze the first, KA^2^, step. In this regard, a reaction was also set up between **5a** and phenyl isothiocyanate, in the presence of CuCl_2_, leading to the formation of the desired product **6a** in 69% yield after 5 h and 100% yield after 18 h (Scheme 4c). In the same set of experiments, **5a** reacted with phenyl isothiocyanate in varying volumes of dry toluene: With a ratio of 0.057 mmol of **5a**/0.015 mL of solvent or 0.057 mmol of **5a**/0.041 mL of solvent, the reaction was completed after 18 h, whereas at a ratio of 0.057 mmol of **5a**/0.072 mL of solvent, a 69% yield was obtained after 5 h and completion was observed after 18 h (Scheme 4c). These findings suggest that copper(II) accelerates the second step of the overall transformation, and as anticipated, the lower the concentration, the lower the rate of the reaction.

Taking into account the literature precedent, with regard to the activation of alkynes with copper(I), and considering the possibility of copper(I) formation in the reaction mixture, which can be like others done in the presence of thiourea byproducts (once phenyl isothiocyanate is added),^23^ we also set up a benchmark reaction with copper(I). As one can see in Scheme 4d, the reaction of **5a** with phenyl isothiocyanate in the presence of CuCl leads to the formation of the desired product **6a** in 46% conversion and 89% chemoselectivity toward the 5-exo-dig S-cyclization-derived product. This observation suggests that the second step (nucleophilic attack and cyclization) is catalyzed by copper(I), as well. As shown in entry 14 of Table 2, replacing CuCl_2_ with CuCl under the optimized conditions leads to 40% formation of **6a**, with 92% chemoselectivity, favoring the 5-exo-dig S-cyclization mode of reactivity. On the basis of the known capability of Cu(II) to be reduced to Cu(I) in the presence of thioureas,^24^ we can hypothesize that in our case also, CuCl_2_ is reduced by thiourea byproducts (generated after the addition of isothiocyanate derivatives) to CuCl, catalyzing the second step of the overall transformation.

**Scheme 5:**
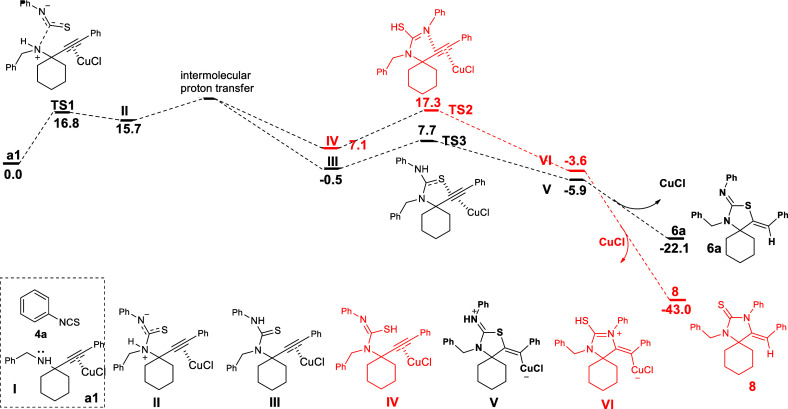
Possible Mechanistic Pathways for the Cu(I)-Catalyzed Cyclization of Propargylamine Thiourea **III** and Its Tautomer, **IV**, Studied via DFT Calculations

To gain additional information for the second step of the overall reaction, density functional theory (DFT) calculations were also carried out in implicit medium simulating toluene as the solvent. Reaction system **a1**, consisting of propargylamine **I** [complexed through its alkyne moiety by Cu(I)] and phenyl isothiocyanate, proceeds initially with a nucleophilic attack of the amine on the carbon of the isothiocyanate group (Scheme 5). Ammonium salt **II** undergoes an intermolecular proton transfer with an activation barrier of ∼7.6 kcal/mol [compared to the higher barriers for intramolecular proton transfer (see Schemes S1 and S2)] to produce thiourea **III**. The nucleophilic addition to the Cu(I)-activated triple bond by S or N, in **III** or **IV**, yields **V** or **VI**, respectively. The 5-exo-dig S-cyclization or 5-exo-dig N-cyclization in the activated alkyne moiety, forming **V** or **VI**, respectively, has an activation barrier of 8.2 or 10.2 kcal/mol, which consequently leads to **6a** or **8**, respectively, in agreement with the selectivity observed toward the thiazolidin-2-imine product. Despite our attempts, CuCl_2_ could not fit into the calculations of Scheme 5, reinforcing the proposition of Cu(I) being the active catalyst of the second step of the overall transformation, where the 5-exo-dig S-cyclization takes place.

**Scheme 6:**
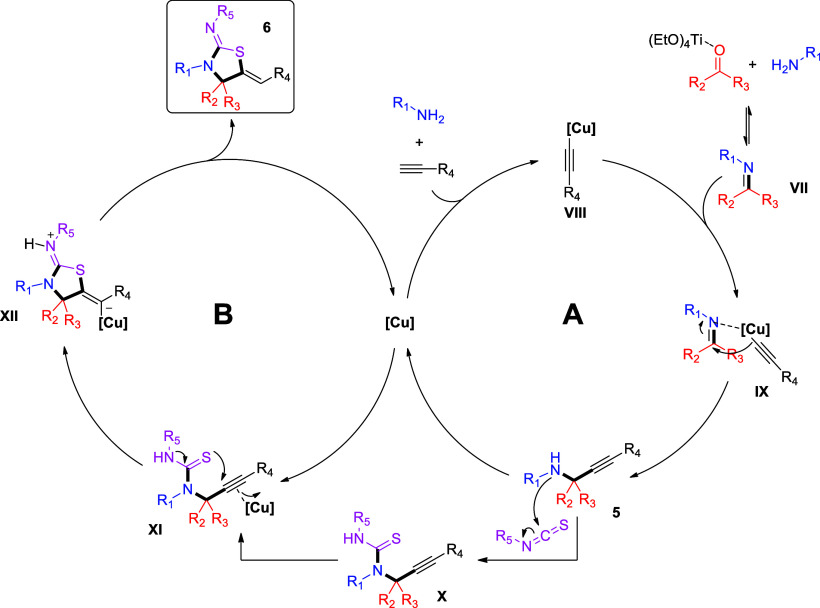
Proposed Mechanism for the One-Pot, Multicomponent Reaction for the Synthesis of Thiazolidin-2-imines

On the basis of our results, as well as the related literature precedent,^9b,12,14,20^ we propose the overall mechanism shown in Scheme 6. Initially, the ketone substrate is activated by Ti(OEt)_4_, followed by the attack of the amine, leading to the formation of a ketimine **VII**. CuCl_2_, with the aid of amine as the base, reacts with the terminal alkyne, toward the formation of copper acetylide species **IX**,^23a,23c,25^ which nucleophilically attacks ketimine intermediate **VII**, leading to the formation of key propargylamine intermediate **5**. To facilitate this nucleophilic attack, an increased reaction temperature (110 °C) is required. After sufficient and/or maximum formation of the propargylamine intermediate, the isothiocyanate substrate is introduced into the reaction mixture and is nucleophilically attacked by the propargylamine nitrogen, toward the formation of thiourea intermediate **X**. Thiourea intermediate **X** is then transformed into the desired thiazolidin-2-imine **6**, via a regioselective and chemoselective 5-exo-dig S-cyclization (hydrothiolation, **XII**). This transformation can proceed without the aid of additives or catalysts (Scheme 4a);^14^ however, it is accelerated by the presence of the *in situ*-formed copper(I) in this one-pot case (Scheme 4c,d), which can be derived by the reduction of CuCl_2_, as discussed above.^23b,24^

## Conclusion

We herein present a one-pot, two-step reaction sequence for the formation of invaluable, synthetically challenging thiazolidin-2-imines, by employing stoichiometric amounts of commercially available starting materials. The desired products are isolated in good to excellent yields. Synthetically useful and biologically relevant moieties are amenable to our transformation, making it appropriate for late-stage functionalization. The first step of the overall reaction involves a KA^2^ coupling, catalyzed by 5% CuCl_2_, toward propargylamine intermediates. Upon introduction of isothiocyanates, propargylamines nucleophilically attack them moving toward the corresponding thiourea intermediates, which, via a regioselective and chemoselective 5-exo-dig S-cyclization, lead to the formation of the desired thiazolidin-2-imines. The copper catalyst partakes in the cycles of both steps, catalyzing the first step toward propargylamines and accelerating the cyclization of the second step. Our DFT calculations suggest that the copper(I)-catalyzed reaction between propargylamines and isothiocyanates proceeds with a lower energy barrier for the pathway leading to S-attack compared to N-attack, resulting in the observed, selective formation of thiazolidin-2-imines. The obtained compounds have the potential for unique biological properties, based on literature precedent and similarities, such as antiproliferative and anticonvulsant drugs, among others.

## Experimental Section

### General Information

All chemicals, starting materials, and catalysts were acquired from commercial sources, and a majority of these were used without further purification, except for cyclohexanone, which was distilled prior to use. All reactions were carried out under an inert atmosphere of argon, in flame-dried, Teflon-sealed, screw-capped pressure tubes or Schlenk tubes, and mixtures were heated in a preheated oil bath. Toluene was dried using standard literature procedures. Dry DMF was purchased from Acros Organics. The course of the reactions was monitored via thin layer chromatography (TLC), using silica gel 60 precoated aluminum sheets (0.2 mm), absorbing at 254 nm (silica gel 60 F254), and/or using a potassium permanganate solution for visualization. All products were isolated by high-pressure gradient column chromatography, using silica gel 60 (230–400 mesh) and mixtures of hexanes with ethyl acetate or hexanes with Et_2_O, as the eluent. NMR spectra were recorded on Bruker Avance-400 MHz or Varian Mercury 200 MHz instruments, using CDCl_3_ as the solvent and its residual solvent peak (at 7.26 ppm for ^1^H and 77.16 ppm for ^13^C) as a reference. NMR spectroscopic data are given in the following order: chemical shift, multiplicity (s, singlet; bs, broad signal; d, doublet; t, triplet; q, quartet; dd, doublet of doublets; dt, doublet of triplets; m, multiplet), coupling constant in hertz, and number of protons. High-resolution mass spectrometry (HRMS) spectra were recorded using a QTOF maxis Impact (Bruker) spectrometer with electrospray ionization (ESI).

### Synthetic Procedure for *N*-Benzyl-1-(phenylethynyl)cyclohexanamine (**5a**)^22^

A flame-dried and argon-purged Schlenk tube, equipped with a stirring bar, was charged with CuCl_2_ (0.067 g, 0.5 mmol), phenylacetylene (0.66 mL, 6 mmol), cyclohexanone (0.518 mL, 6 mmol), and benzylamine (0.655 mL, 6 mmol). The reaction mixture was then heated in a preheated oil bath at 110 °C for 20 h. Then, the mixture was dissolved with CHCl_3_, transferred to a vial, and concentrated under reduced pressure. Purification via column chromatography, using a gradient of 99/1 to 95/5 petroleum ether (P.E.)/EtOAc, afforded the product as a semi-orange oil in 40% yield (0.686 g, 2.37 mmol): ^1^H NMR (400 MHz, CDCl_3_) δ 7.50–7.37 (m, 4H), 7.36–7.29 (m, 5H), 7.26–7.22 (m, 1H), 3.99 (s, 2H), 2.06–1.96 (m, 2H), 1.78–1.48 (m, 7H), 1.36–1.21 (m, 1H).

### General Procedure for the Synthesis of Thiazolidin-2-imines **6**

A Teflon-sealed screw-capped pressure tube or a Schlenk tube, flame-dried and purged with Ar, containing a magnetic stirring bar, was charged with CuCl_2_ (0.02 mmol, 0.1 equiv), a ketone (0.4 mmol, 1 equiv), a terminal alkyne (0.4 mmol, 1 equiv), and a primary amine (0.4 mmol, 1 equiv). The mixture was then stirred with the help of an external magnet for a short time, until all of the solids had been sufficiently solvated. Then, Ti(OEt)_4_ (0.2 mmol, 0.5 equiv) was added. In most cases, upon addition of Ti(OEt)_4_, the reaction mixture turned into a sludge that could not be stirred. Then, 0.5 mL (unless otherwise mentioned) of dry toluene was added, and the reaction mixture was stirred with the help of an external magnet, to allow sufficient mixing of the starting materials, catalyst, and additive. The reaction mixture was then heated in a preheated oil bath, at 110 °C for 22 h. Then, the reaction mixture was cooled to room temperature, the isothiocyanate derivative (0.4 mmol, 1 equiv) added, and the reaction mixture stirred at room temperature for 5 h, unless otherwise mentioned. After completion of the reaction, the mixture was dissolved in chloroform, transferred to a vial, and condensed under reduced pressure. Purification with column chromatography, using a mixture of hexanes with ethyl acetate or hexanes with Et_2_O, as the eluent, afforded the desired products. When the crude mixture was not solid, dry loading the crude mixture into the column proved to be more efficient.

#### (*Z*)-*N*-[(*Z*)-1-Benzyl-4-benzylidene-3-thia-1-azaspiro[4.5]decan-2-ylidene]aniline (**6a**)^14^

Product **6a** was synthesized according to the general procedure, using 0.05 mL of dry toluene. When the reaction was completed, the mixture was dissolved in DCM and passed through a Celite short plug. Purification with column chromatography (99/1 to 98/2 P.E./Et_2_O) led to a yellow–colorless oil in 68% yield (0.272 mmol, 115 mg): ^1^H NMR (400 MHz, CDCl_3_) δ 7.42–7.19 (m, 12H), 7.04 (t, *J* = 7.4 Hz, 1H), 6.98 (s, 1H), 6.94 (d, *J* = 7.8 Hz, 2H), 4.85 (s, 2H), 2.08 (d, *J* = 13.7 Hz, 2H), 1.95–1.66 (m, 7H), 1.40–1.18 (m, 1H); ^13^C{^1^H} NMR (101 MHz, CDCl_3_) δ 155.6, 151.5, 139.9, 139.4, 136.3, 128.9, 128.6, 128.6, 128.4, 127.4, 126.7, 126.7, 123.0, 122.4, 122.4, 70.3, 46.2, 33.5, 24.9, 23.0.

#### (*Z*)-*N*-[(*Z*)-1-Benzyl-4-benzylidene-3-thia-1-azaspiro[4.6]undecan-2-ylidene]aniline (**6b**)

Product **6b** was synthesized according to the general procedure, using 0.25 mL of dry toluene. When the reaction was completed, the mixture was dissolved in DCM and passed through a Celite short pad. Purification with column chromatography (99/1 to 97/3 P.E./Et_2_O) afforded the product as a yellow oil in 61% yield (0.244 mmol, 107 mg): ^1^H NMR (400 MHz, CDCl_3_) δ 7.38 (d, *J* = 7.6 Hz, 2H), 7.36–7.27 (m, 7H), 7.25–7.19 (m, 3H), 7.03 (t, *J* = 7.4 Hz, 1H), 6.93 (d, *J* = 7.8 Hz, 2H), 6.68 (s, 1H), 4.90 (s, 2H), 2.19–2.03 (m, 4H), 1.77–1.54 (m, 8H); ^13^C{^1^H} NMR (101 MHz, CDCl_3_) δ 154.6, 151.7, 140.1, 139.3, 136.2, 129.0, 128.7, 128.6, 128.4, 127.3, 127.0, 126.9, 123.0, 122.4, 120.7, 74.9, 46.5, 38.4, 31.7, 24.5; ESI-HRMS (*m*/*z*) calcd for C_29_H_31_N_2_S (M + H)^+^ 439.2202, found 439.2205.

#### (*Z*)-*N*-[(*Z*)-1-Benzyl-4-benzylidene-5.8-dioxaspiro-3-thia-1-azaspiro[4.5]decan-2-ylidene]aniline (**6c**)

Product **6c** was synthesized according to the general procedure, using 0.35 mL of dry toluene. Once phenyl isothiocyanate was added, the reaction mixture was stirred at 60 °C for 10 h. Purification via column chromatography (99/1 to 75/25 P.E./Et_2_O) afforded the product as a yellowish oil in 89% yield (0.354 mmol, 171 mg): ^1^H NMR (400 MHz, CDCl_3_) δ 7.42–7.19 (m, 12H), 7.04 (t, *J* = 7.4 Hz, 1H), 6.97 (s, 1H), 6.94 (d, *J* = 7.8 Hz, 2H), 4.86 (s, 2H), 3.99 (s, 4H), 2.31 (td, *J* = 13.2, 4.7 Hz, 2H), 2.15–1.97 (m, 4H), 1.89–1.79 (m, 2H); ^13^C{^1^H} NMR (101 MHz, CDCl_3_) δ 155.4, 151.3, 139.3, 139.2, 136.0, 129.0, 128.6, 128.4, 127.5, 126.8, 126.7, 123.1, 122.3, 121.5, 107.6, 69.3, 64.6, 64.5, 46.3, 31.9, 30.9; ESI-HRMS (*m*/*z*) calcd for C_30_H_31_N_2_O_2_S (M + H)^+^ 483.2101, found 483.2103.

#### (*Z*)-*N*-[(*Z*)-3-Benzyl-5-benzylidene-4-methyl-4-propylthiazolidin-2-ylidene]aniline (**6d**)

Product **6d** was synthesized according to the general procedure. Purification twice via column chromatography (99/1 P.E./EtOAc) afforded the product as a yellow solid in 75% yield (0.301 mmol, 124 mg): ^1^H NMR (400 MHz, CDCl_3_) δ 7.46 (d, *J* = 7.6 Hz, 2H), 7.37–7.26 (m, 8H), 7.25–7.16 (m, 2H), 7.06 (t, *J* = 7.4 Hz, 1H), 7.00 (d, *J* = 8.3 Hz, 2H), 6.43 (s, 1H), 5.05 (d, *J* = 16.0 Hz, 1H), 4.55 (d, *J* = 16.0 Hz, 1H), 1.96 (ddd, *J* = 15.5, 12.1, 4.2 Hz, 1H), 1.69 (ddd, *J* = 14.1, 13.2, 4.7 Hz, 1H), 1.49 (s, 3H), 1.41–1.17 (m, 2H), 0.79 (t, *J* = 7.3 Hz, 3H); ^13^C{^1^H} NMR (101 MHz, CDCl_3_) δ 155.6, 151.6, 139.2, 139.0, 136.4, 129.0, 128.7, 128.4, 128.2, 127.6, 127.0, 127.0, 123.1, 122.5, 118.5, 72.2, 46.3, 44.0, 28.6, 17.0, 14.1; ESI-HRMS (*m*/*z*) calcd for C_27_H_29_N_2_S (M + H)^+^ 413.2046, found 413.2064.

#### (*Z*)-*N*-[(*Z*)-3-Benzyl-5-benzylidene-4-hexyl-4-methylthiazolidin-2-ylidene]aniline (**6e**)

Product **6e** was synthesized according to the general procedure, with the first step implemented at 110 °C for 44 h. Purification twice via column chromatography (99/1 to 98/2 P.E./Et_2_O) afforded the product as a yellow oil in 52% yield (0.209 mmol, 95 mg): ^1^H NMR (400 MHz, CDCl_3_) δ 7.47 (d, *J* = 7.1 Hz, 2H), 7.38–7.28 (m, 8H), 7.21 (m, 2H), 7.08 (t, *J* = 7.4 Hz, 1H), 7.01 (d, *J* = 7.3 Hz, 2H), 6.44 (s, 1H), 5.03 (d, *J* = 16.0 Hz, 1H), 4.59 (d, *J* = 16.0 Hz, 1H), 1.97 (ddd, *J* = 13.3, 12.1, 3.3 Hz, 1H), 1.78–1.66 (m, 1H), 1.50 (s, 3H), 1.39–1.09 (m, 8H), 0.87 (t, *J* = 7.0 Hz, 3H); ^13^C{^1^H} NMR (101 MHz, CDCl_3_) δ 155.6, 151.7, 139.3, 139.0, 136.4, 129.0, 128.7, 128.4, 128.2, 127.6, 127.0, 123.1, 122.5, 118.5, 72.2, 46.3, 41.6, 31.8, 29.3, 28.7, 23.6, 22.7, 14.2; ESI-HRMS (*m*/*z*) calcd for C_30_H_35_N_2_S (M + H)^+^ 455.2515, found 455.2519.

#### (*Z*)-*N*-[(*Z*)-1-Benzyl-4-(4-methylbenzylidene)-3-thia-1-azaspiro[4.5]decan-2-ylidene]aniline (**6f**)

Product **6f** was synthesized according to the general procedure, using 0.3 mL of dry toluene. Purification via column chromatography (P.E. to 99/1 P.E./Et_2_O) afforded the product as a pale yellow oil in 71% yield (0.285 mmol, 125 mg): ^1^H NMR (400 MHz, CDCl_3_) δ 7.40–7.29 (m, 4H), 7.29–7.17 (m, 5H), 7.14 (d, *J* = 8.5 Hz, 2H), 7.02 (t, *J* = 6.8 Hz, 1H), 6.96–6.89 (m, 3H), 4.83 (s, 2H), 2.33 (s, 3H), 2.07 (d, *J* = 13.4 Hz, 2H), 1.94–1.65 (m, 7H), 1.36–1.18 (m, 1H); ^13^C{^1^H} NMR (101 MHz, CDCl_3_) δ 155.8, 151.5, 139.4, 138.8, 137.3, 133.5, 129.3, 128.9, 128.5, 128.4, 126.7, 123.0, 122.4, 122.4, 70.2, 46.2, 33.5, 24.9, 23.1, 21.3; ESI-HRMS (*m*/*z*) calcd for C_29_H_31_N_2_S (M + H)^+^ 439.2202, found 439.2221.

#### (*Z*)-*N*-[(*Z*)-1-Benzyl-4-(4-methoxybenzylidene)-3-thia-1-azaspiro[4.5]decan-2-ylidene]aniline (**6g**)

Product **6g** was synthesized according to the general procedure, using 0.3 mL of dry toluene. Purification via column chromatography (P.E. to 98/2 P.E./Et_2_O) afforded the product as a semiyellow oil in 70% yield (0.279 mmol, 127 mg): ^1^H NMR (400 MHz, CDCl_3_) δ 7.35 (d, *J* = 7.5 Hz, 2H), 7.32–7.27 (m, 2H), 7.25–7.17 (m, 5H), 7.01 (t, *J* = 7.4 Hz, 1H), 6.94–6.88 (m, 3H), 6.85 (d, *J* = 8.6 Hz, 2H), 4.82 (s, 2H), 3.77 (s, 3H), 2.04 (d, *J* = 13.2 Hz, 2H), 1.91–1.64 (m, 7H), 1.31–1.23 (m, 1H); ^13^C{^1^H} NMR (101 MHz, CDCl_3_) δ 158.8, 155.8, 151.5, 139.4, 137.5, 129.9, 129.0, 128.9, 128.4, 126.7, 122.9, 122.4, 122.0, 114.0, 70.2, 55.4, 46.2, 33.4, 24.9, 23.1; ESI-HRMS (*m*/*z*) calcd for C_29_H_31_N_2_OS (M + H)^+^ 455.2150, found 455.2179.

#### (Z)-*N*-[(Z)-1-Benzyl-4-(4-chlorobenzylidene)-3-thia-1-azaspiro[4.5]decan-2-ylidene]aniline (**6h**)

Product **6h** was synthesized according to the general procedure. Purification via column chromatography (100/0 to 99/1 P.E./Et_2_O) afforded the product as a yellow-white solid in 90% yield (0.359 mmol, 165 mg): ^1^H NMR (400 MHz, CDCl_3_) δ 7.40–7.16 (m, 11H), 7.04 (t, *J* = 7.4 Hz, 1H), 6.97–6.86 (m, 3H), 4.85 (bs, 2H), 2.05 (d, *J* = 13.5 Hz, 2H), 1.93–1.68 (m, 7H), 1.29–1.23 (m, 1H); ^13^C{^1^H} NMR (101 MHz, CDCl_3_) δ 155.0, 151.3, 140.8, 139.2, 134.8, 133.0, 129.8, 129.0, 128.7, 128.4, 126.8, 126.7, 123.1, 122.3, 121.1, 70.3, 46.2, 33.4, 24.8, 23.0; ESI-HRMS (*m*/*z*) calcd for C_28_H_28_ClN_2_S (M + H)^+^ 459.1656, found 459.1656.

#### (*Z*)-*N*-{(*Z*)-1-Benzyl-4-[4-(trifluoromethyl)benzylidene]-3-thia-1-azaspiro[4.5]decan-2-ylidene]aniline (**6i**)

Product **6i** was synthesized according to the general procedure, using 0.3 mL of dry toluene. Purification via column chromatography (99/1 hexane/Et_2_O) afforded the product as a white solid in 55% yield (0.219 mmol, 108 mg): ^1^H NMR (400 MHz, CDCl_3_) δ 7.60 (d, *J* = 8.1 Hz, 2H), 7.46–7.32 (m, 6H), 7.32–7.23 (m, 3H), 7.07 (t, *J* = 7.4 Hz, 1H), 7.00 (s, 1H), 6.94 (d, *J* = 7.3 Hz, 2H), 4.87 (s, 2H), 2.09 (d, *J* = 13.5 Hz, 2H), 1.99–1.66 (m, 7H), 1.38–1.27 (m, 1H); ^13^C{^1^H} NMR (101 MHz, CDCl_3_) δ 154.8, 151.3, 143.0, 139.9, 139.9, 139.2, 129.1 (q, *J* = 32.4 Hz), 129.0, 128.8, 128.5, 126.9, 126.7, 125.5 (q, *J* = 3.8 Hz), 123.3, 122.8, 122.3, 120.9, 120.2, 70.4, 46.3, 33.5, 24.8, 23.0; ^19^F{^1^H} NMR (376 MHz, CDCl_3_) δ −62.5; ESI-HRMS (*m*/*z*) calcd for C_29_H_28_F_3_N_2_S (M + H)^+^ 493.1920, found 493.1934.

#### (*Z*)-*N*-[(*Z*)-1-Benzyl-4-(3-methylbenzylidene)-3-thia-1-azaspiro[4.5]decan-2-ylidene]aniline (**6j**)

Product **6j** was synthesized according to the general procedure. Purification via column chromatography (99/1 P.E./EtOAc) afforded the product as a yellow-white solid in 62% yield (0.249 mmol, 109 mg): ^1^H NMR (400 MHz, CDCl_3_) δ 7.43–7.18 (m, 9H), 7.17–7.09 (m, 2H), 7.08–6.99 (m, 2H), 6.98–6.90 (m, 3H), 4.85 (s, 2H), 2.35 (s, 3H), 2.08 (d, *J* = 13.2 Hz, 2H), 1.96–1.66 (m, 7H), 1.37–1.19 (m, 1H); ^13^C{^1^H} NMR (101 MHz, CDCl_3_) δ 155.6, 151.4, 139.6, 139.4, 138.2, 136.3, 129.5, 128.9, 128.5, 128.4, 128.2, 126.7, 126.7, 125.4, 123.0, 122.6, 122.4, 70.2, 46.2, 33.5, 24.9, 23.0, 21.6; ESI-HRMS (*m*/*z*) calcd for C_29_H_31_N_2_S (M + H)^+^ 439.2202, found 439.2204.

#### (*Z*)-*N*-[(*Z*)-1-Benzyl-4-heptylidene-3-thia-1-azaspiro[4.5]decan-2-ylidene]aniline (**6k**)^14^

Product **6k** was synthesized according to the general procedure, using 0.25 mL of dry toluene. The first step of the reaction was heating the mixture at 110 °C for 44 h. Purification twice via column chromatography (99/1 P.E./EtOAc) afforded the product as a yellow oil in 34% yield (0.135 mmol, 58 mg): ^1^H NMR (200 MHz, CDCl_3_) δ 7.42–7.18 (m, 7H), 7.02 (t, *J* = 7.4 Hz, 1H), 6.93 (d, *J* = 8.6 Hz, 2H), 5.86 (t, *J* = 7.0 Hz, 1H), 4.77 (s, 2H), 2.01 (q, *J* = 7.8, 7.3 Hz, 2H), 1.90 (d, *J* = 12.4 Hz, 2H), 1.82–1.51 (m, 8H), 1.45–1.19 (m, 8H), 0.87 (t, *J* = 6.5 Hz, 3H); ^13^C{^1^H} NMR (101 MHz, CDCl_3_) δ 156.1, 152.0, 139.6, 138.3, 128.9, 128.4, 126.8, 126.7, 123.1, 123.0, 122.5, 68.9, 46.1, 33.4, 31.8, 31.7, 29.2, 29.0, 24.9, 22.8, 22.7, 14.2; ESI-HRMS (*m*/*z*) calcd for C_28_H_37_N_2_S (M + H)^+^ 433.2672, found 433.2677.

#### (*Z*)-*N*-[(*Z*)-4-Benzylidene-1-(4-methylbenzyl)-3-thia-1-azaspiro[4.5]decan-2-ylidene]aniline (**6l**)

Product **6l** was synthesized according to the general procedure. Purification via column chromatography (98/2 to 96/4 P.E./EtOAc) afforded the product as a semiyellow solid in 70% yield (0.28 mmol, 123 mg): ^1^H NMR (400 MHz, CDCl_3_) δ 7.42–7.21 (m, 9H), 7.19–7.12 (m, 2H), 7.06 (t, *J* = 7.5 Hz, 1H), 7.02–6.91 (m, 3H), 4.85 (s, 2H), 2.37 (s, 3H), 2.10 (d, *J* = 13.5 Hz, 2H), 1.99–1.70 (m, 7H), 1.40–1.25 (m, 1H); ^13^C{^1^H} NMR (101 MHz, CDCl_3_) δ 155.6, 151.4, 139.9, 136.3, 136.3, 136.2, 129.1, 128.9, 128.6, 127.4, 126.6, 123.0, 122.4, 122.4, 70.3, 46.0, 33.5, 24.9, 23.0, 21.2; ESI-HRMS (*m*/*z*) calcd for C_29_H_31_N_2_S (M + H)^+^ 439.2202, found 439.2204.

#### (*Z*)-*N*-[(*Z*)-4-Benzylidene-1-(4-fluorobenzyl)-3-thia-1-azaspiro[4.5]decan-2-ylidene]aniline (**6m**)

Product **6m** was synthesized according to the general procedure. Purification via column chromatography (99/1 P.E./Et_2_O) afforded the product as a white solid in 56% yield (0.224 mmol, 99 mg): ^1^H NMR (400 MHz, CDCl_3_) δ 7.41–7.22 (m, 9H), 7.10–6.99 (m, 4H), 6.94 (d, *J* = 7.7 Hz, 2H), 4.82 (s, 2H), 2.10 (d, *J* = 12.8 Hz, 2H), 1.96–1.69 (m, 7H), 1.40–1.23 (m, 1H); ^13^C{^1^H} NMR (101 MHz, CDCl_3_) δ 161.9 (d, *J* = 244.2 Hz), 155.7, 151.3, 139.7, 136.3, 135.1 (d, *J* = 3.2 Hz), 129.0, 128.6, 128.4, 128.3, 127.4, 123.1, 122.6, 122.3, 115.2 (d, *J* = 21.4 Hz), 70.3, 45.6, 33.5, 24.9, 23.0; ^19^F{^1^H} NMR (376 MHz, CDCl_3_) δ −116.55; ESI-HRMS (*m*/*z*) calcd for C_28_H_28_FN_2_S (M + H)^+^ 443.1952, found 443.1941.

#### (*Z*)-*N*-[(*Z*)-4-Benzylidene-1-(4-methoxybenzyl)-3-thia-1-azaspiro[4.5]decan-2-ylidene]aniline (**6n**)

Product **6n** was synthesized according to the general procedure. Purification via column chromatography (99/1 to 98/2 P.E./Et_2_O) afforded the product as a white-yellow solid in 46% yield (0.185 mmol, 84 mg): ^1^H NMR (400 MHz, CDCl_3_) δ 7.37–7.17 (m, 9H), 7.03 (t, *J* = 7.4 Hz, 1H), 6.99–6.88 (m, 3H), 6.88–6.81 (m, 2H), 4.78 (bs, 2H), 3.79 (s, 3H), 2.05 (d, *J* = 13.4 Hz, 2H), 1.92–1.66 (m, 7H), 1.31–1.21 (m, 1H); ^13^C{^1^H} NMR (101 MHz, CDCl_3_) δ 158.5, 155.6, 151.5, 151.5, 139.9, 136.4, 131.5, 128.9, 128.6, 128.0, 127.4, 123.0, 122.4, 122.4, 113.9, 70.3, 55.4, 45.7, 33.5, 24.9, 23.1; ESI-HRMS (*m*/*z*) calcd for C_29_H_31_N_2_OS (M + H)^+^ 455.2152, found 455.2159.

#### (*Z*)-*N*-[(*Z*)-4-Benzylidene-1-octyl-3-thia-1-azaspiro[4.5]decan-2-ylidene]aniline (**6o**)

Product **6o** was synthesized according to the general procedure. Purification via column chromatography (99/1 P.E./Et_2_O) afforded the product as an orange solid in 73% yield (0.291 mmol, 130 mg): ^1^H NMR (200 MHz, CDCl_3_) δ 7.38–7.11 (m, 7H), 7.10–6.82 (m, 4H), 3.39 (t, *J* = 8.0 Hz, 2H), 2.05 (d, *J* = 11.1 Hz, 2H), 1.93–1.53 (m, 9H), 1.47–1.09 (m, 11H), 0.85 (t, *J* = 6.0 Hz, 3H); ^13^C{^1^H} NMR (101 MHz, CDCl_3_) δ 154.8, 151.9, 140.4, 136.5, 128.9, 128.5, 128.5, 127.2, 122.8, 122.4, 121.9, 70.1, 43.7, 33.4, 32.0, 29.9, 29.6, 29.4, 27.3, 24.9, 23.0, 22.8, 14.2; ESI-HRMS (*m*/*z*) calcd for C_29_H_38_N_2_S (M + H)^+^ 447.2828, found 447.2814.

#### 5-[(2*Z*,4*Z*)-4-Benzylidene-2-(phenylimino)-3-thia-1-azaspiro[4.5]decan-1-yl]pentan-1-ol (**6p**)

Product **6p** was synthesized according to the general procedure. Purification via column chromatography (99/1 to 1/1 P.E./EtOAc) afforded the product as a yellow oil in 57% yield (0.228 mmol, 96 mg): ^1^H NMR (200 MHz, CDCl_3_) δ 7.40–7.09 (m, 7H), 7.02 (t, *J* = 7.3 Hz, 1H), 6.96–6.82 (m, 3H), 3.65 (t, *J* = 6.2 Hz, 2H), 3.43 (dd, *J*_1_ = *J*_2_ = 7.6 Hz, 2H), 2.07 (d, *J* = 10.2 Hz, 1H), 1.96–1.53 (m, 12H), 1.53–1.09 (m, 3H); ^13^C{^1^H} NMR (101 MHz, CDCl_3_) δ 155.4, 151.7, 140.1, 136.4, 129.0, 128.5, 127.2, 123.0, 122.5, 122.1, 70.2, 62.7, 43.6, 33.4, 32.5, 29.3, 24.9, 23.4, 23.0; ESI-HRMS (*m*/*z*) calcd for C_26_H_33_N_2_OS (M + H)^+^ 421.2308, found 421.2298.

#### (*Z*)-*N*-[(*Z*)-1-Benzyl-4-benzylidene-3-thia-1-azaspiro[4.5]decan-2-ylidene]-4-methylaniline (**6q**)

Product **6q** was synthesized according to the general procedure, in which an additional 0.1 mL of dry toluene was added when *p*-tolyl isothiocyanate was added and the reaction mixture was stirred at rt for 9 h. Purification via column chromatography (99/1 to 98/2 P.E./EtOAc) afforded the product as a semiyellow solid in 51% yield (0.203 mmol, 89 mg): ^1^H NMR (400 MHz, CDCl_3_) δ 7.41–7.27 (m, 8H), 7.25–7.18 (m, 2H), 7.05 (d, *J* = 8.0 Hz, 2H), 6.95 (s, 1H), 6.80 (d, *J* = 8.2 Hz, 2H), 4.82 (s, 2H), 2.30 (s, 3H), 2.05 (d, *J* = 13.9 Hz, 2H), 1.96–1.62 (m, 7H), 1.33–1.21 (m, 1H); ^13^C{^1^H} NMR (101 MHz, CDCl_3_) δ 155.5, 148.9, 140.0, 139.5, 136.4, 132.3, 129.6, 128.6, 128.6, 128.4, 127.3, 126.7, 126.7, 122.3, 122.1, 70.2, 46.2, 33.5, 24.9, 23.1, 21.0; ESI-HRMS (*m*/*z*) calcd for C_29_H_31_N_2_S (M + H)^+^ 439.2202, found 439.2213.

#### (*Z*)-*N*-[(*Z*)-1-Benzyl-4-benzylidene-3-thia-1-azaspiro[4.5]decan-2-ylidene]-4-methoxyaniline (**6r**)

Product **6r** was synthesized according to the general procedure. Once *p*-methoxyphenyl isothiocyanate was added, the reaction mixture was stirred at 60 °C for 9 h. Purification via column chromatography (99/1 to 97/3 P.E./EtOAc) afforded the product as an orange solid in 81% yield (0.323 mmol, 147 mg): ^1^H NMR (400 MHz, CDCl_3_) δ 7.37–7.28 (m, 8H), 7.25–7.18 (m, 2H), 6.96 (s, 1H), 6.85 (d, *J* = 8.9 Hz, 2H), 6.80 (d, *J* = 9.0 Hz, 2H), 4.82 (s, 2H), 3.78 (s, 3H), 2.05 (d, *J* = 13.3 Hz, 2H), 1.94–1.68 (m, 7H), 1.32–1.24 (m, 1H); ^13^C{^1^H} NMR (101 MHz, CDCl_3_) δ 156.0, 155.8, 144.9, 140.0, 139.5, 136.4, 128.6, 128.6, 128.4, 128.4, 127.4, 126.7, 123.2, 122.3, 114.3, 70.3, 55.6, 46.2, 33.5, 24.9, 23.1; ESI-HRMS (*m*/*z*) calcd for C_29_H_31_N_2_OS (M + H)^+^ 455.2152, found 455.2148.

#### (*Z*)-*N*-[(*Z*)-1-Benzyl-4-benzylidene-3-thia-1-azaspiro[4.5]decan-2-ylidene]-4-chloroaniline (**6s**)^14^

Product **6s** was synthesized according to the general procedure. Once *p*-chlorophenyl isothiocyanate was added, the reaction mixture was left at rt for 9 h. Purification via column chromatography (99/1 to 98/2 P.E./EtOAc) afforded the product as a white solid in 60% yield (0.24 mmol, 110 mg): ^1^H NMR (400 MHz, CDCl_3_) δ 7.44–7.16 (m, 12H), 7.00 (s, 1H), 6.92–6.80 (m, 2H), 4.83 (s, 2H), 2.08 (d, *J* = 13.3 Hz, 2H), 1.97–1.67 (m, 7H), 1.38–1.19 (m, 1H); ^13^C{^1^H} NMR (101 MHz, CDCl_3_) δ 156.1, 150.1, 139.4, 139.1, 136.2, 129.0, 128.6, 128.6, 128.5, 128.1, 127.5, 126.8, 126.6, 123.7, 122.7, 70.5, 46.2, 33.5, 24.8, 23.0.

#### (*Z*)-*N*-[(*Z*)-1-Benzyl-4-benzylidene-3-thia-1-azaspiro[4.5]decan-2-ylidene]-4-bromoaniline (**6t**)

Product **6t** was synthesized according to the general procedure. Once *p*-chlorophenyl isothiocyanate was added, the reaction mixture was left at 60 °C for 5 h. Purification via column chromatography (99/1 to 97/3 P.E./EtOAc) afforded the product as a white solid in 54% yield (0.216 mmol, 109 mg): ^1^H NMR (400 MHz, CDCl_3_) δ 7.40–7.20 (m, 12H), 6.99 (s, 1H), 6.81 (d, *J* = 8.4 Hz, 2H), 4.85 (s, 2H), 2.06 (d, *J* = 14.8 Hz, 2H), 1.94–1.64 (m, 7H), 1.33–1.24 (m, 1H); ^13^C{^1^H} NMR (101 MHz, CDCl_3_): δ 156.3, 150.2, 139.2, 138.9, 136.1, 131.9, 128.7, 128.6, 128.5, 127.6, 126.8, 126.6, 124.3, 122.9, 116.0, 70.6, 46.2, 33.5, 24.8, 23.0; ESI-HRMS (*m*/*z*) calcd for C_28_H_28_BrN_2_S (M + H)^+^ 503.1151, found 503.1151.

#### (*Z*)-*N*-[(*Z*)-1-Benzyl-4-benzylidene-3-thia-1-azaspiro[4.5]decan-2-ylidene]-4-(trifluoromethyl)aniline (**6u**)

Product **6u** was synthesized according to the general procedure. Purification via column chromatography (99/1 to 98/2 P.E./EtOAc) afforded the product as a yellow solid in 71% yield (0.284 mmol, 140 mg): ^1^H NMR (400 MHz, CDCl_3_) δ 7.48 (d, *J* = 8.2 Hz, 2H), 7.41–7.18 (m, 10H), 7.00 (s, 1H), 6.97 (d, *J* = 8.1 Hz, 2H), 4.82 (s, 2H), 2.07 (d, *J* = 13.4 Hz, 2H), 1.97–1.65 (m, 7H), 1.32–1.22 (m, 1H); ^13^C{^1^H} NMR (101 MHz, CDCl_3_) δ 156.1, 154.5, 139.1, 139.0, 136.1, 128.7, 128.6, 128.5, 127.6, 126.9, 126.6, 126.18 (q, *J* = 3.7 Hz), 124.75 (q, *J* = 32.3 Hz), 123.5, 123.0, 122.5, 70.5, 46.3, 33.5, 24.8, 23.0; ^19^F{^1^H} NMR (376 MHz, CDCl_3_) δ −61.58; ESI-HRMS (*m*/*z*) calcd for C_29_H_28_F_3_N_2_S (M + H)^+^ 493.1920, found 493.1918.

#### (*Z*)-*N*-[(*Z*)-1-Benzyl-4-benzylidene-3-thia-1-azaspiro[4.5]decan-2-ylidene]hexan-1-amine (**6v**)

Product **6v** was synthesized according to the general procedure. Once 1-hexyl isothiocyanate was added, the reaction mixture was stirred at 60 °C for 5 h. Purification twice via column chromatography (99/1 to 94/6 P.E./EtOAc) afforded the product as a yellow oil in 68% yield (0.273 mmol, 118 mg): ^1^H NMR (400 MHz, CDCl_3_) δ 7.47–7.38 (m, 4H), 7.33–7.25 (m, 5H), 7.23–7.16 (m, 1H), 6.97 (s, 1H), 4.68 (s, 2H), 3.25 (t, *J* = 7.0 Hz, 2H), 2.00 (d, *J* = 12.0 Hz, 2H), 1.87–1.69 (m, 7H), 1.59–1.46 (m, 2H), 1.36–1.19 (m, 7H), 0.87 (t, *J* = 6.8 Hz, 3H); ^13^C{^1^H} NMR (101 MHz, CDCl_3_) δ 154.3, 140.2, 140.1, 136.7, 128.7, 128.5, 128.2, 127.3, 126.7, 126.5, 121.9, 69.4, 54.5, 46.0, 33.2, 31.8, 31.7, 27.1, 25.0, 23.2, 22.8, 14.2; ESI-HRMS (*m*/*z*) calcd for C_28_H_36_N_2_SNa (M + Na)^+^ 455.2491, found 455.2489.

#### Methyl 2-{(*Z*)-[(*Z*)-1-Benzyl-4-benzylidene-3-thia-1-azaspiro[4.5]decan-2-ylidene]amino}acetate (**6w**)

Product **6w** was synthesized according to the general procedure. Once methyl 2-isothiocyanatoacetate was added, the reaction mixture was stirred at 60 °C for 5 h. Purification via column chromatography (99/1 to 91/9 P.E./EtOAc) afforded the product as a pale yellow oil in 76% yield (0.304 mmol, 128 mg): ^1^H NMR (400 MHz, CDCl_3_) δ 7.45–7.36 (m, 4H), 7.36–7.25 (m, 5H), 7.20 (t, *J* = 6.8 Hz, 1H), 6.99 (s, 1H), 4.75 (s, 2H), 4.11 (s, 2H), 3.70 (s, 3H), 2.00 (d, *J* = 13.8 Hz, 2H), 1.88–1.66 (m, 7H), 1.30–1.24 (m, 1H); ^13^C{^1^H} NMR (101 MHz, CDCl_3_) δ 171.8, 158.9, 139.4, 138.9, 136.4, 128.6, 128.6, 128.3, 127.5, 126.7, 126.6, 122.7, 70.4, 55.2, 51.9, 46.0, 33.3, 24.9, 23.0; ESI-HRMS (*m*/*z*) calcd for C_25_H_29_N_2_O_2_S (M + H)^+^ 421.1944, found 421.1947.

#### (*Z*)-*N*-[(*Z*)-1-Benzyl-4-(4-chlorobenzylidene)-5.8-dioxaspiro-3-thia-1-azaspiro[4.5]decan-2-ylidene]aniline (**6x**)

Product **6x** was synthesized according to the general procedure. Once phenyl isothiocyanate was added, the reaction mixture was stirred at 60 °C for 10 h. Purification via column chromatography (98/2 to 80/20 P.E./EtOAc) afforded the product as a white solid in 79% yield (0.315 mmol, 163 mg): ^1^H NMR (400 MHz, CDCl_3_) δ 7.39–7.18 (m, 11H), 7.04 (t, *J* = 7.4 Hz, 1H), 6.94–6.86 (m, 3H), 4.84 (s, 2H), 3.98 (s, 4H), 2.29 (td, *J* = 13.5, 4.9 Hz, 2H), 2.11–1.93 (m, 4H), 1.89–1.78 (m, 2H); ^13^C{^1^H} NMR (101 MHz, CDCl_3_) δ 154.9, 151.3, 140.4, 139.2, 134.6, 133.2, 129.9, 129.0, 128.8, 128.5, 126.8, 126.7, 123.3, 122.3, 120.2, 107.5, 69.3, 64.6, 64.6, 46.3, 31.9, 31.0; ESI-HRMS (*m*/*z*) calcd for C_30_H_30_ClN_2_O_2_S (M + H)^+^ 517.1711, found 517.1720.

#### (*Z*)-*N*-[(*Z*)-1-Benzyl-4-(4-methoxybenzylidene)-5.8-dioxaspiro-3-thia-1-azaspiro[4.5]decan-2-ylidene]aniline (**6y**)

Product **6y** was synthesized according to the general procedure. Once phenyl isothiocyanate was added, the reaction mixture was stirred at 60 °C for 8 h. Purification via column chromatography (98/2 to 1/1 P.E./EtOAc) afforded the product as a white-yellow solid in 63% yield (0.252 mmol, 129 mg): ^1^H NMR (400 MHz, CDCl_3_) δ 7.39–7.16 (m, 9H), 7.02 (t, *J* = 7.4 Hz, 1H), 6.93–6.88 (m, 3H), 6.86 (d, *J* = 8.3 Hz, 2H), 4.82 (s, 2H), 3.98 (s, 4H), 3.80 (s, 3H), 2.34–2.19 (m, 2H), 2.13–1.95 (m, 4H), 1.88–1.74 (m, 2H); ^13^C{^1^H} NMR (101 MHz, CDCl_3_) δ 158.9, 155.6, 151.4, 139.3, 136.9, 130.0, 128.9, 128.7, 128.4, 126.7, 126.7, 123.1, 122.3, 121.1, 114.0, 107.7, 69.2, 64.6, 64.5, 55.4, 46.2, 31.9, 30.9; ESI-HRMS (*m*/*z*) calcd for C_31_H_33_N_2_O_3_S (M + H)^+^ 513.2206, found 513.2207.

#### (*Z*)-*N*-[(*Z*)-4-Benzylidene-1-(4-fluorobenzyl)-5.8-dioxaspiro-3-thia-1-azaspiro[4.5]decan-2-ylidene]aniline (**6z**)

Product **6z** was synthesized according to the general procedure. Once phenyl isothiocyanate was added, the reaction mixture was stirred at rt for 8 h. Purification via column chromatography (97/3 to 91/9 P.E./EtOAc) afforded the product as an orange solid in 72% yield (0.288 mmol, 144 mg): ^1^H NMR (400 MHz, CDCl_3_) δ 7.37–7.19 (m, 9H), 7.06–6.97 (m, 3H), 6.96 (s, 1H), 6.90 (d, *J* = 8.4 Hz, 2H), 4.79 (s, 2H), 3.99 (s, 4H), 2.27 (td, *J* = 14.5, 5.0 Hz, 2H), 2.14–1.95 (m, 4H), 1.91–1.78 (m, 2H); ^13^C{^1^H} NMR (101 MHz, CDCl_3_) δ 161.9 (d, *J* = 244.2 Hz), 155.5, 151.2, 139.2, 136.0, 134.9 (d, *J* = 3.0 Hz), 129.2, 128.6, 128.4, 128.3, 127.6, 123.2, 122.3, 121.6, 115.2 (d, *J* = 21.4 Hz), 107.6, 69.3, 64.6, 64.5, 45.7, 31.9, 31.0; ^19^F{^1^H} NMR (376 MHz, CDCl_3_) δ −116.56; ESI-HRMS (*m*/*z*) calcd for C_30_H_30_FN_2_O_2_S (M + H)^+^ 501.2007, found 501.2017.

#### 5-[(2*Z*,4*Z*)-4-Benzylidene-2-(phenylimino)-5.8-dioxaspiro-3-thia-1-azaspiro[4.5]decan-1-yl]pentan-1-ol (**6aa**)

Product **6aa** was synthesized according to the general procedure. Once phenyl isothiocyanate was added, the reaction mixture was stirred at 60 °C for 8 h. Purification via column chromatography (95/5 to 33/67 P.E./EtOAc) afforded the product as an orange solid in 77% yield (0.310 mmol, 148 mg): ^1^H NMR (400 MHz, CDCl_3_) δ 7.35–7.17 (m, 7H), 7.03 (t, *J* = 7.4 Hz, 1H), 6.96–6.88 (m, 3H), 4.01 (s, 4H), 3.65 (t, *J* = 6.4 Hz, 2H), 3.46 (t, *J* = 7.8 Hz, 2H), 2.26 (td, *J* = 14.4, 5.0 Hz, 2H), 2.14–1.99 (m, 4H), 1.87 (dd, *J* = 14.1, 4.6 Hz, 2H), 1.80–1.68 (m, 2H), 1.69–1.57 (m, 2H), 1.53–1.41 (m, 2H); ^13^C{^1^H} NMR (101 MHz, CDCl_3_) δ 155.5, 151.2, 139.4, 136.0, 128.9, 128.5, 128.5, 127.4, 123.2, 122.5, 121.2, 107.5, 69.3, 64.5, 64.5, 62.4, 43.6, 32.3, 31.8, 30.8, 29.2, 23.3; ESI-HRMS (*m*/*z*) calcd for C_28_H_35_N_2_O_3_S (M + H)^+^ 479.2363, found 479.2366.

#### (*Z*)-*N*-[(*Z*)-4-(4-Chlorobenzylidene)-1-octyl-3-thia-1-azaspiro[4.5]decan-2-ylidene]aniline (**6ab**)

Product **6ab** was synthesized according to the general procedure. Once phenyl isothiocyanate was added, the reaction mixture was stirred at rt for 8 h. Purification via column chromatography (P.E. to 99/1 P.E./EtOAc) afforded the product as a yellow oil in 67% yield (0.268 mmol, 129 mg): ^1^H NMR (400 MHz, CDCl_3_) δ 7.31–7.22 (m, 4H), 7.20 (d, *J* = 8.2 Hz, 2H), 7.03 (t, *J* = 7.4 Hz, 1H), 6.92 (d, *J* = 7.3 Hz, 2H), 6.88 (s, 1H), 3.42 (dd, *J* = 8.9, 6.8 Hz, 2H), 2.12–1.98 (m, 2H), 1.91–1.65 (m, 9H), 1.43–1.22 (m, 12H), 0.88 (t, *J* = 7.0 Hz, 3H); ^13^C{^1^H} NMR (101 MHz, CDCl_3_) δ 154.4, 151.8, 141.4, 135.0, 132.9, 129.8, 129.0, 128.7, 123.0, 122.4, 120.7, 70.2, 43.8, 33.4, 32.0, 29.9, 29.6, 29.5, 27.4, 24.9, 23.0, 22.8, 14.2; ESI-HRMS (*m*/*z*) calcd for C_29_H_38_ClN_2_S (M + H)^+^ 481.2439, found 481.2441.

### Scale-up Reaction Conditions for Thiazolidin-2-imine **6a**

Following our optimized conditions for the synthesis of thiazolidine-2-imines, a Teflon-sealed screw-capped pressure tube, flame-dried and purged with Ar, containing a magnetic stirring bar, was charged with CuCl_2_ (0.027 g, 0.2 mmol), cyclohexanone (0.414 mL, 4 mmol), phenylacetylene (0.439 mL, 4 mmol), and benzylamine (0.437 mL, 4 mmol). The mixture was then stirred with the help of an external magnet for a short time, until all of the solids were sufficiently solvated. Then, Ti(OEt)_4_ (0.42 mL, 2 mmol) was added. Then, 3 mL of dry toluene was added, and the reaction mixture was stirred with the help of an external magnet, to allow sufficient mixing of the starting materials, catalyst, and additive. The reaction mixture was then heated in a preheated oil bath at 110 °C for 22 h. Then, the reaction mixture was cooled to room temperature, phenyl isothiocyanate (0.478 mL, 4 mmol) added, and the reaction mixture stirred at room temperature for 6 h. After completion of the reaction, the mixture was dissolved in chloroform, transferred to a vial, and condensed under reduced pressure. Purification via column chromatography (P.E. to 98/2 P.E./Et_2_O) afforded the product as a yellowish-colorless oil in 70% yield (1.189 g, 2.8 mmol).

### Computational Methods

The Kohn–Sham formulation of density functional theory was employed.^26^ The meta-hybrid density functional M06^27^ has been used with the extended double-ζ quality Def2-SVPP basis set for all of the static calculations.^28^ This combination of a density functional and a basis set has been found to provide good performance in homogeneous gold catalysis.^29^ All geometry optimizations have been carried out using tight convergence criteria and a pruned grid for numerical integration with 99 radial shells and 590 angular points per shell. In some challenging cases, this grid was enlarged to 175 radial shells and 974 points per shell for first-row atoms and 250 shells and 974 points per shell for heavier elements. These challenging optimizations are usually associated with very soft vibrational modes (usually internal rotations). Analysis of the normal modes obtained via diagonalization of the Hessian matrix was used to confirm the topological nature of each stationary point. The wave function stability for each optimized structure has also been checked. Solvation effects have been taken into account variationally throughout the optimization procedures via the polarizable continuum model (PCM)^30^ using parameters for toluene and taking advantage of the smooth switching function developed by York and Karplus.^31^ All of the calculations performed in this work have been carried out with Gaussian 09.^32^

## Data Availability

The data underlying this study are available in the published article and its Supporting Information.

## Supporting Information Available

^1^H, ^13^C{^1^H}, and ^19^F{^1^H} NMR spectra for all compounds **6**, Cartesian coordinates for all of the structures computed in this work, reaction profiles including discarded, noncompetitive paths and solvent effects, and crystallographic data for **6t** (PDF)
