# Supplementary material for: Copper-Catalyzed One-Pot Synthesis of Thiazolidin-2-imines
Source: J Org Chem. 2024 May 10;89(11):7727–40. doi: 10.1021/acs.joc.4c00394 (PMC11190980; doi:10.1021/acs.joc.4c00394)
Supplement: Supplementary file 1 — jo4c00394_si_001.pdf [file jo4c00394_si_001.pdf]

Supporting Information

for

Copper-Catalyzed One-Pot Synthesis of Thiazolidin-2-Imines

by

Leandros P. Zorba,[a] Ioannis Stylianakis,[b] Nikolaos Tsoureas,[c] Antonios Kolocouris,[b] Georgios C. Vougioukalakis[a,*]

[a]Laboratory of Organic Chemistry, Department of Chemistry, National and Kapodistrian University of Athens, Panepistimiopolis, 15771 Athens, Greece
[b]Laboratory of Medicinal Chemistry, Section of Pharmaceutical Chemistry, Department of Pharmacy, National and Kapodistrian University of Athens, Panepistimioupolis-Zografou, 15771 Athens, Greece
[c]Laboratory of Inorganic Chemistry, Department of Chemistry, National and Kapodistrian University of Athens, Panepistimioupolis, 15771 Athens, Greece

E-mail: vougiouk@ chem.uoa.gr

Table of contents

1. NMR spectra for thiazolidin-2-imines 6………………………………...S2-S33

2. Supporting schemes for DFT calculations concerning the intramolecular *vs* intermolecular proton transfer…………………………………………..........S34

3. Cartesian coordinates of the structures of Scheme 5……………………S35-S44

4. X-ray crystallographic data for 6t……………………………………………S45

5. References……………………………………………………………………S46

1. NMR spectra for thiazolidin-2-imines 6

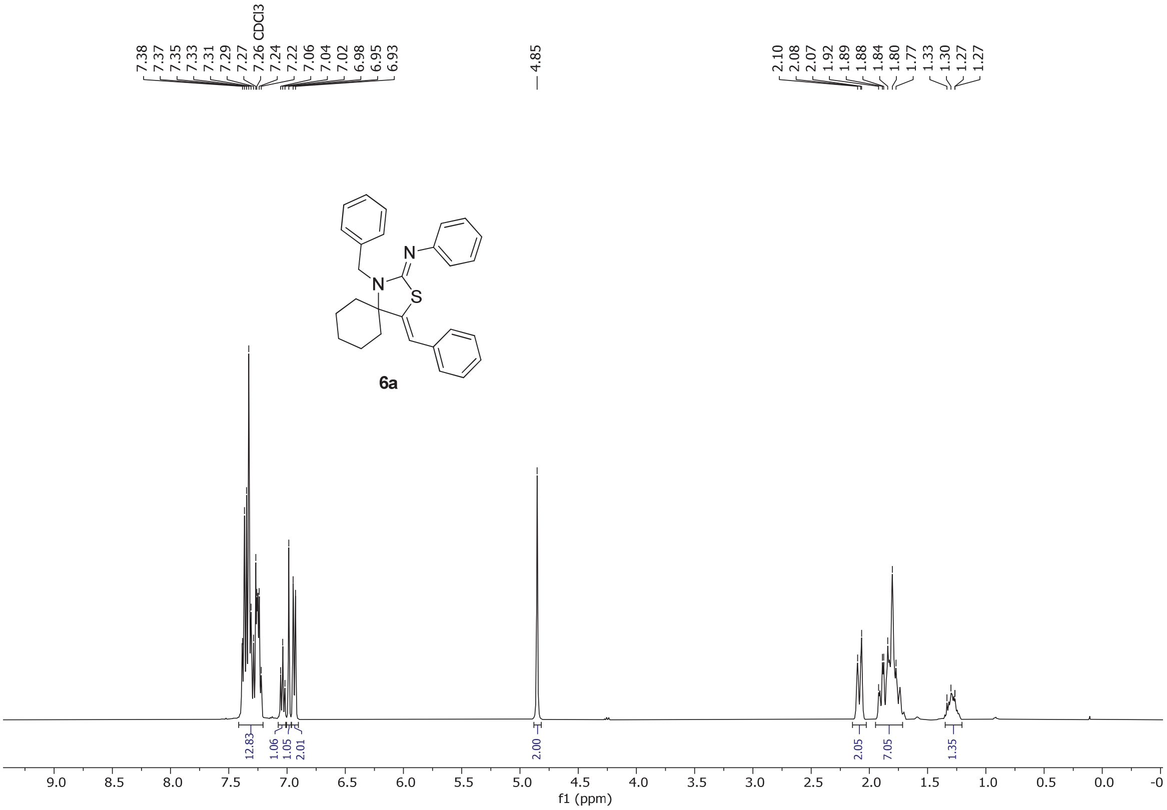

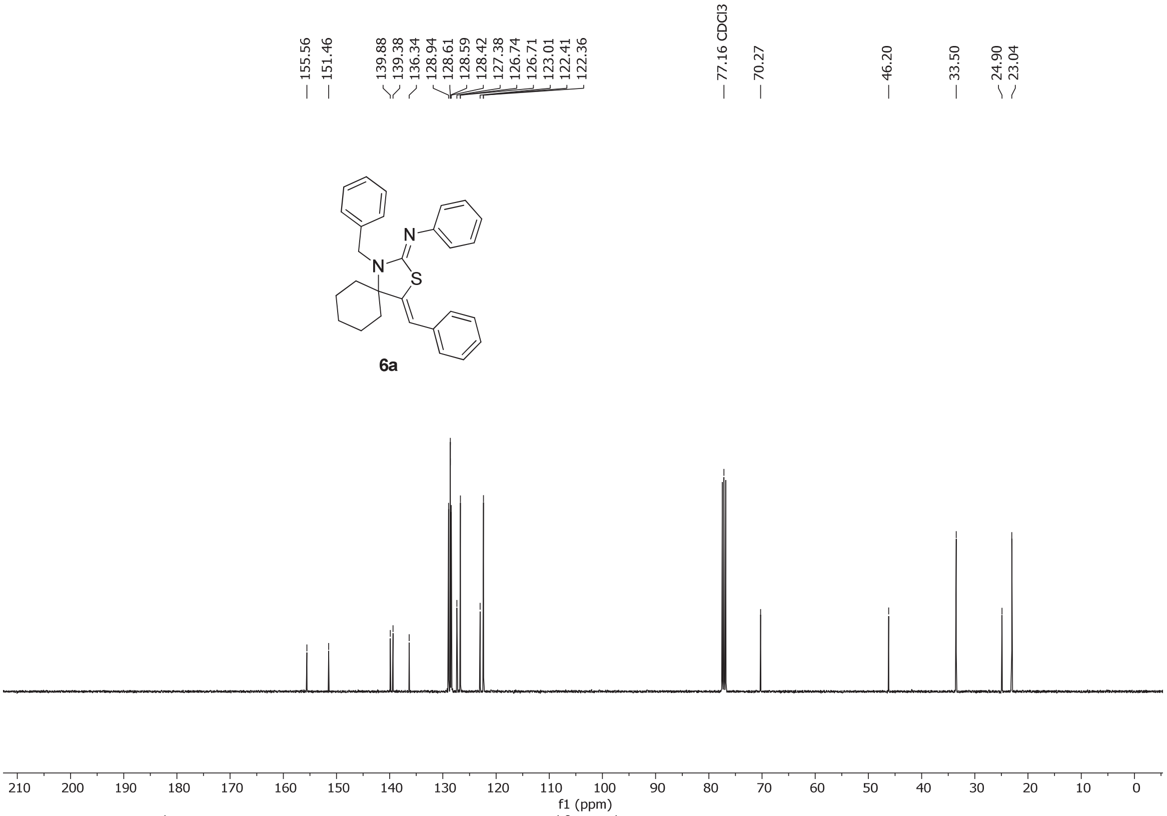

Figure S1: ^{1}H-NMR (400 MHz, up) and ^{13}C{^{1}H}-NMR (101 MHz, bottom) spectra for **6a** in $CDCl_3$.

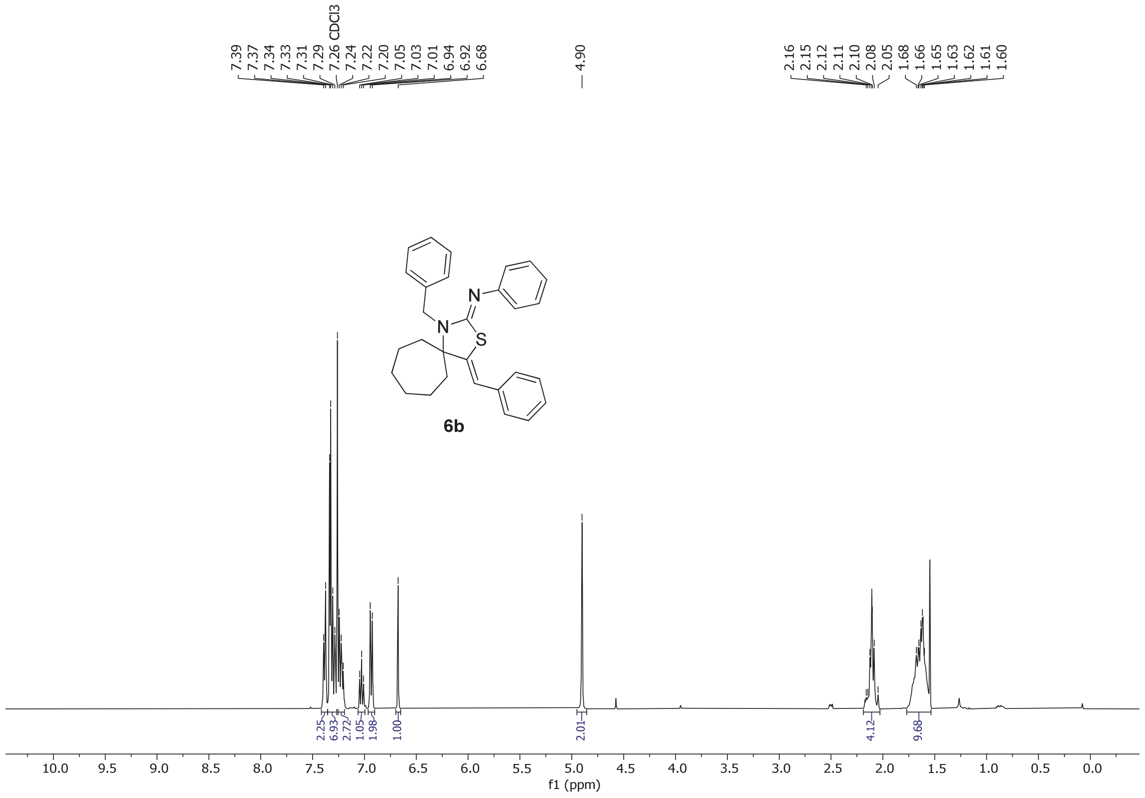

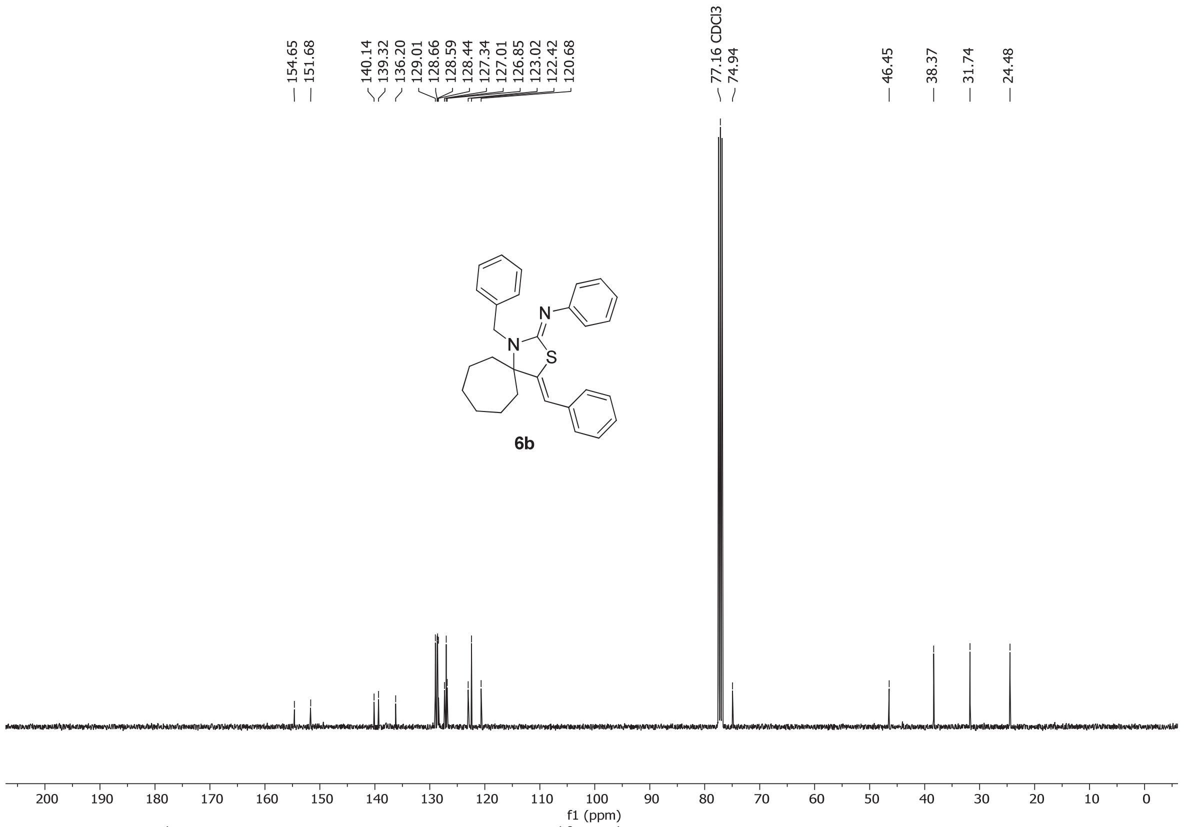

Figure S2: ^{1}H-NMR (400 MHz, up) and $^{13}C\{^{1}H\}$-NMR (101 MHz, bottom) spectra for **6b** in $CDCl_3$.

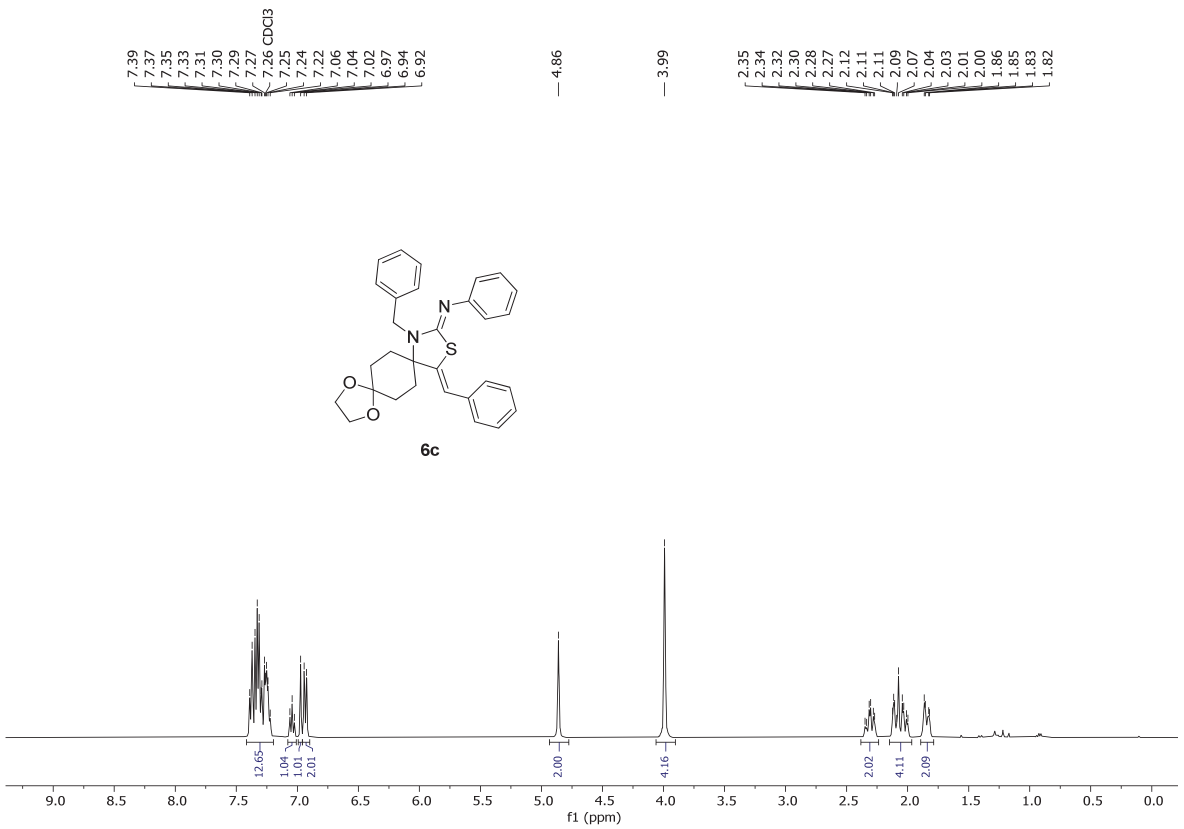

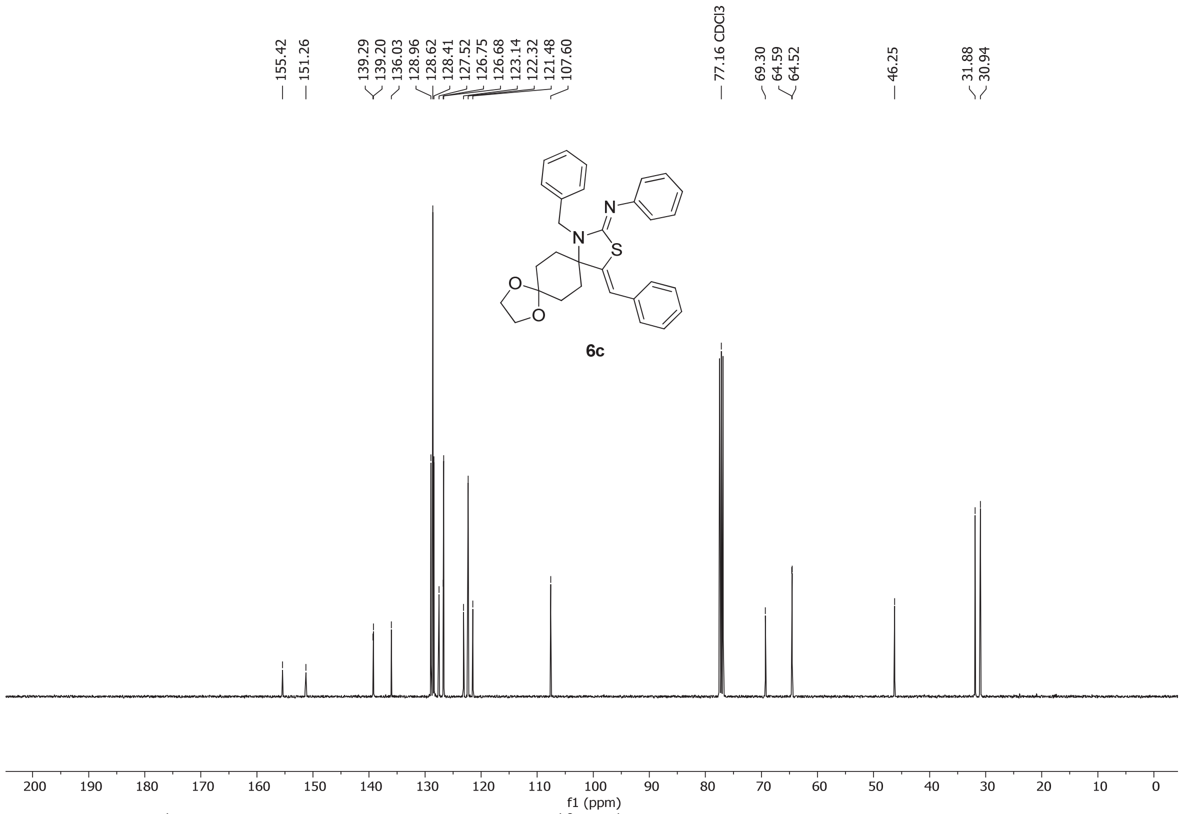

Figure S3: ^{1}H-NMR (400 MHz, up) and $^{13}C\{^{1}H\}$-NMR (101 MHz, bottom) spectra for **6c** in $CDCl_3$.

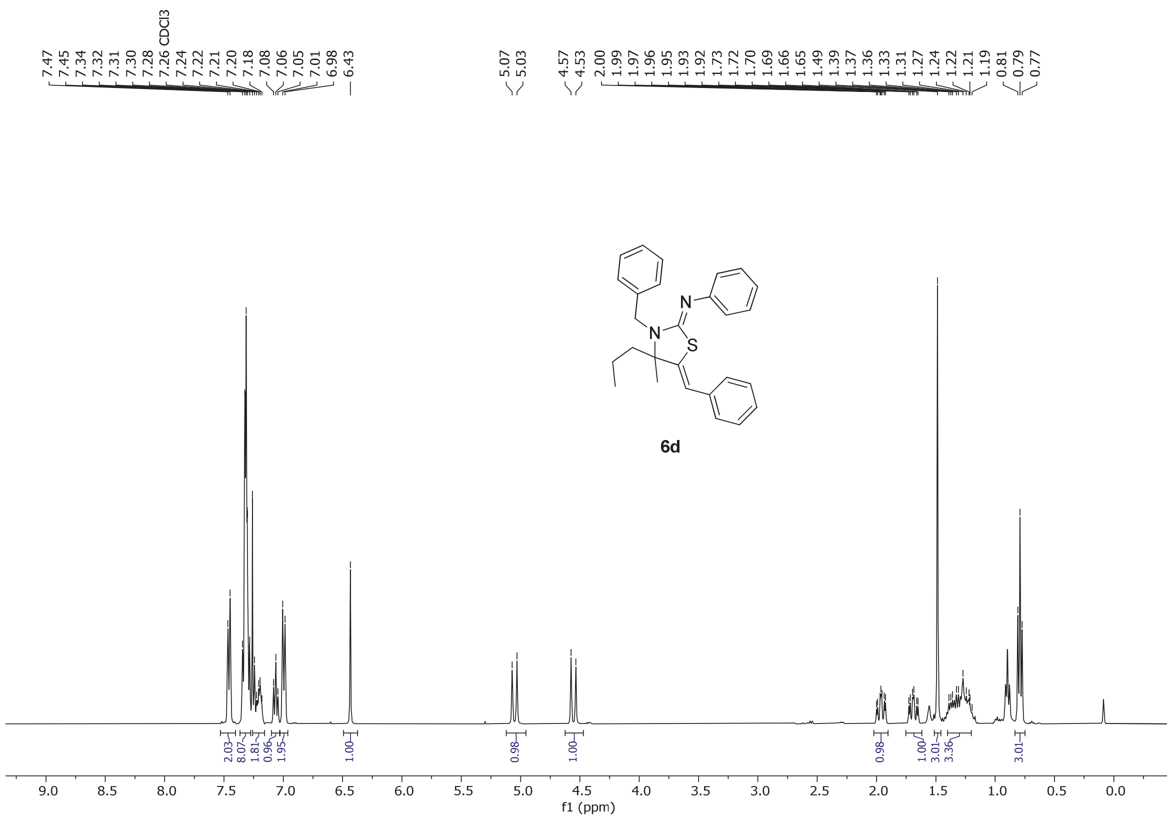

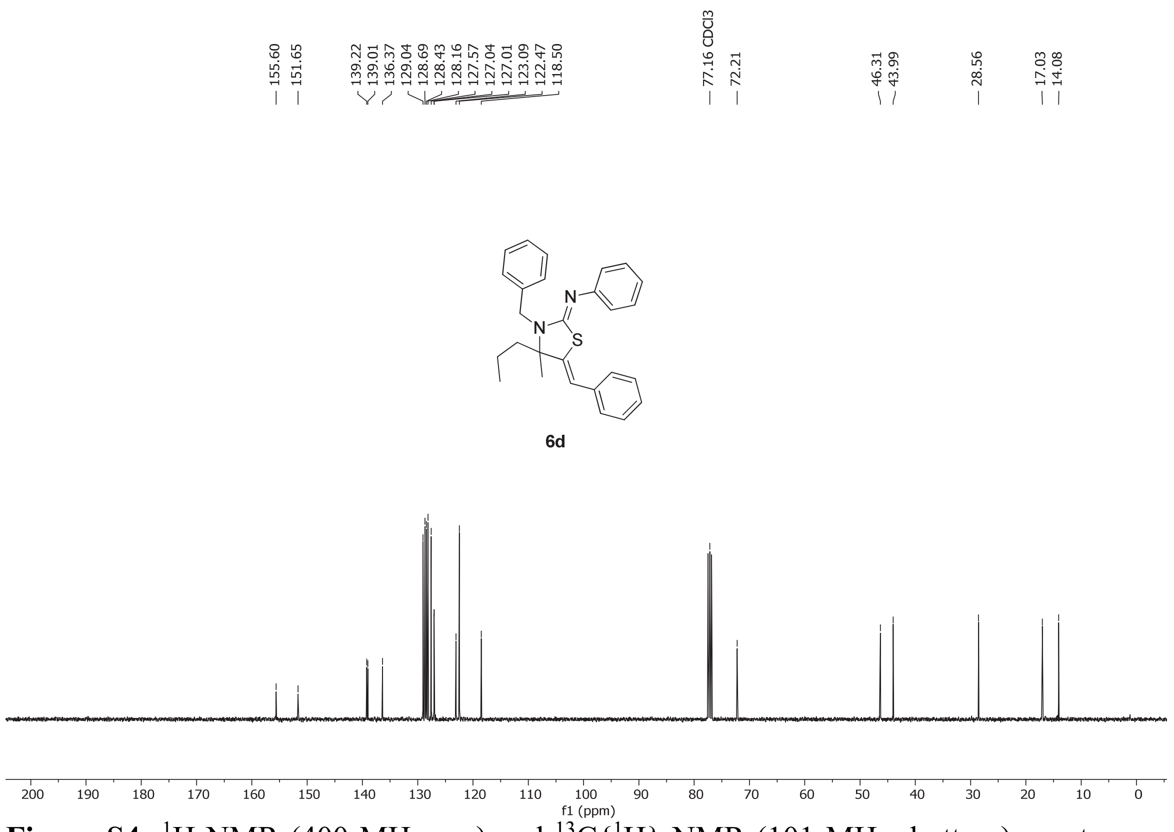

Figure S4: ^{1}H-NMR (400 MHz, up) and ^{13}C{^{1}H}-NMR (101 MHz, bottom) spectra for **6d** in $CDCl_3$.

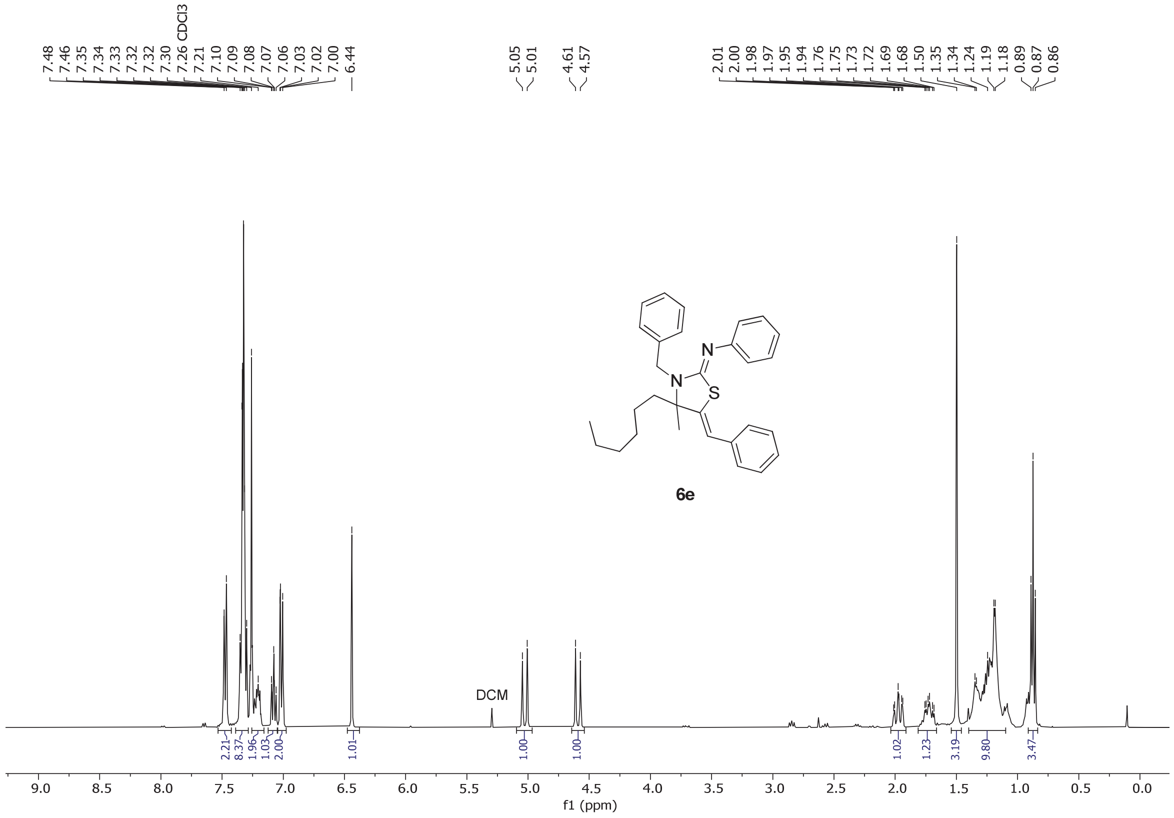

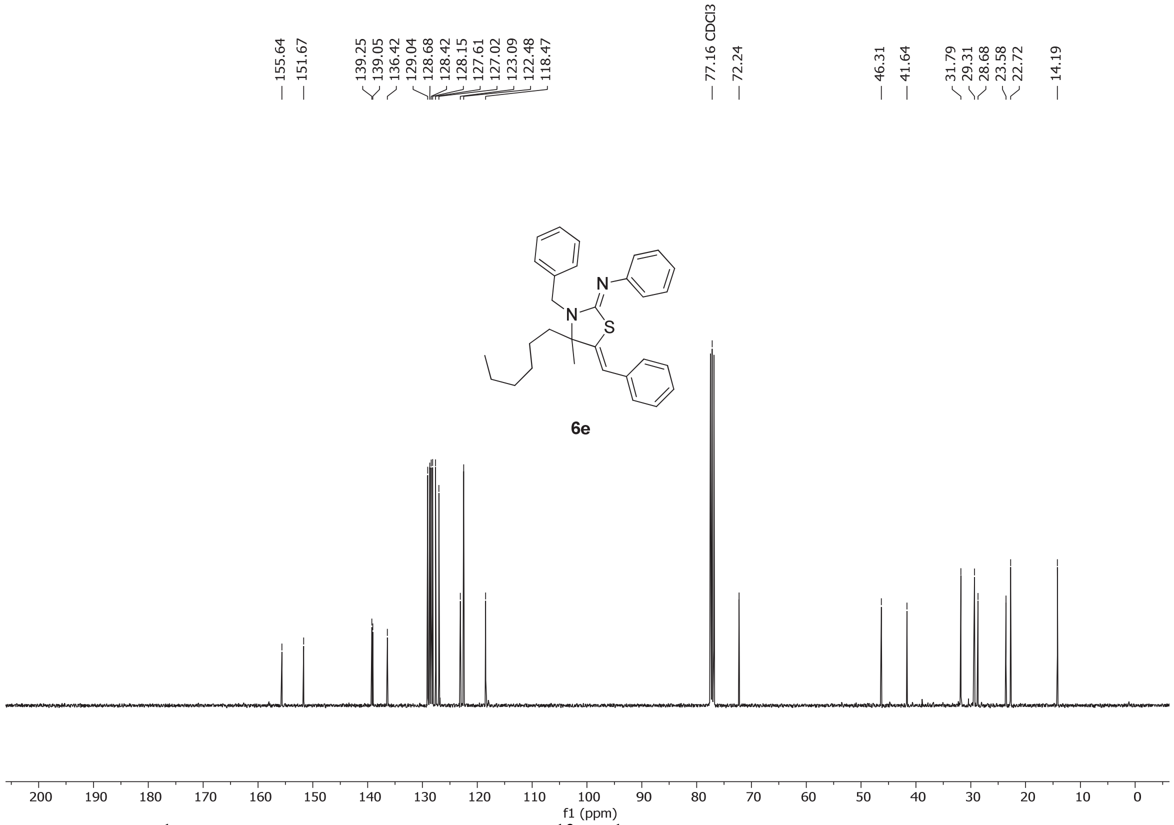

Figure S5: ^{1}H-NMR (400 MHz, up) and $^{13}C\{^{1}H\}$-NMR (101 MHz, bottom) spectra for **6e** in $CDCl_3$.

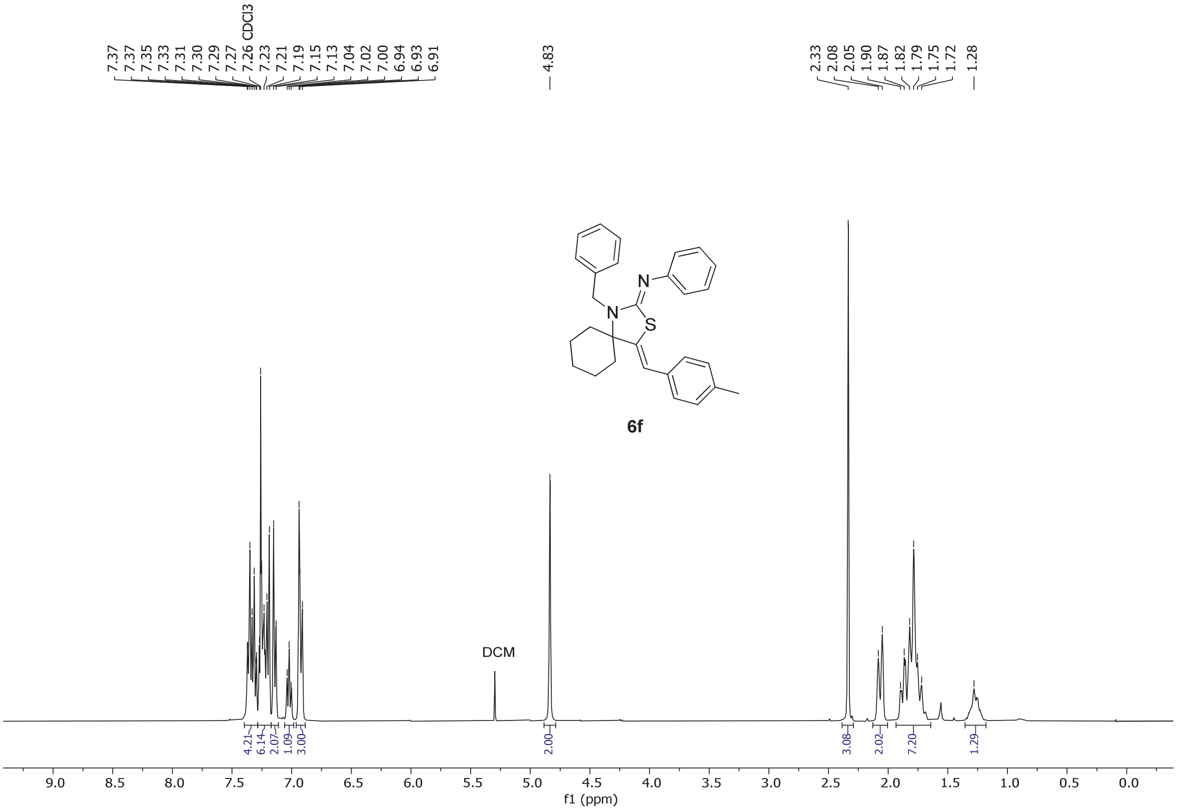

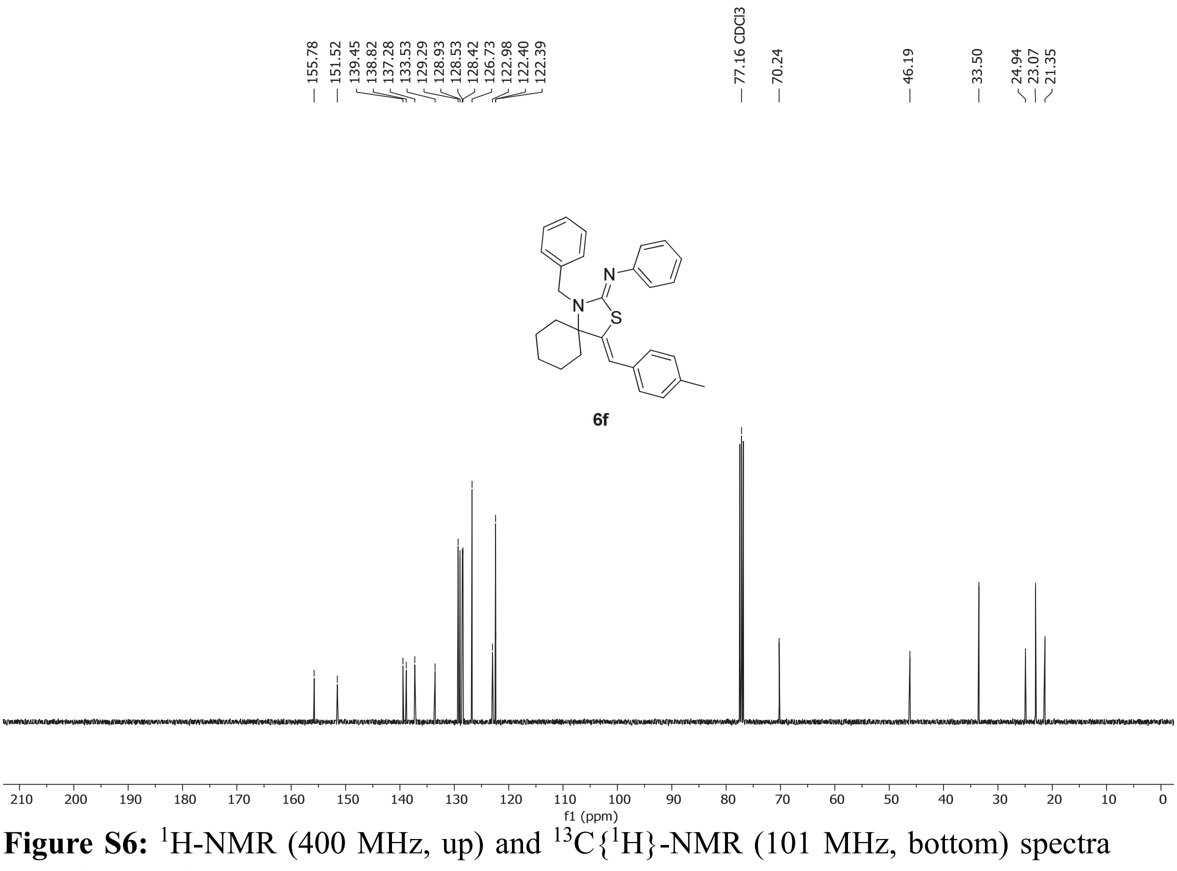

Figure S6: ^{1}H-NMR (400 MHz, up) and ^{13}C{^{1}H}-NMR (101 MHz, bottom) spectra for **6f** in $CDCl_3$.

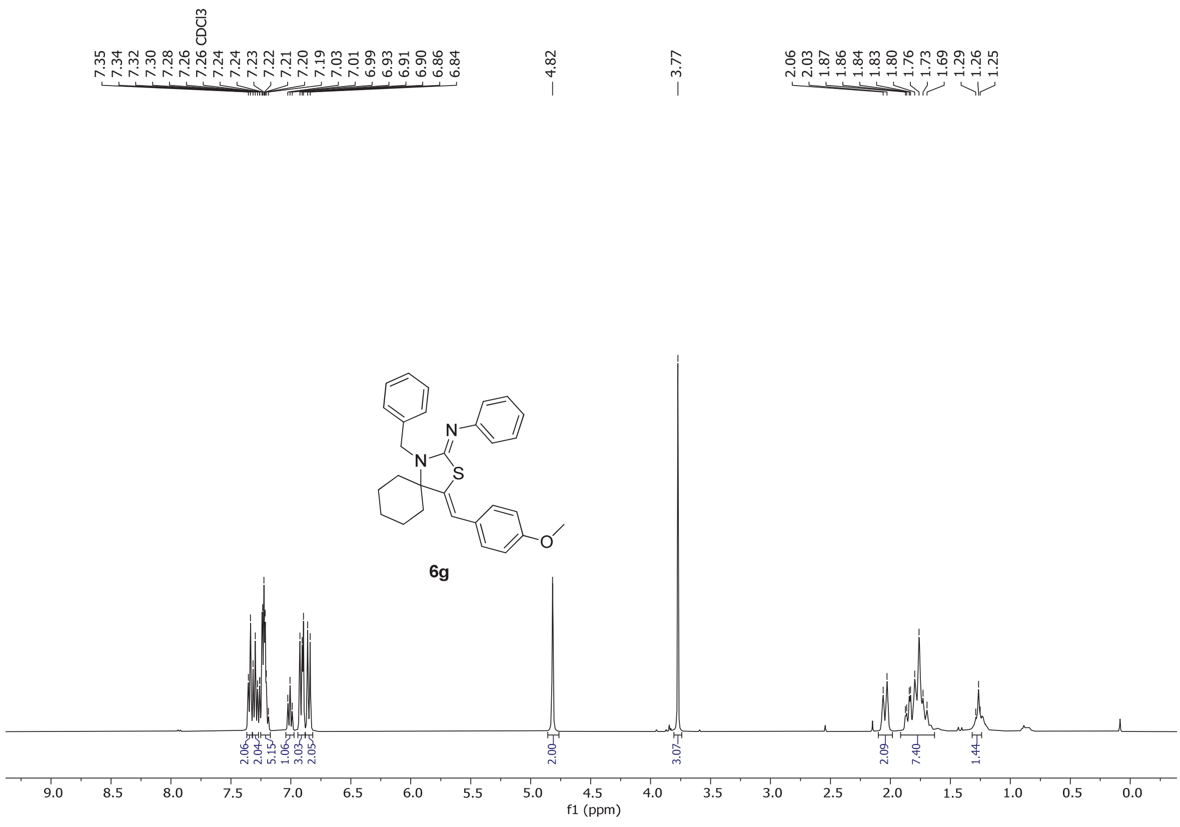

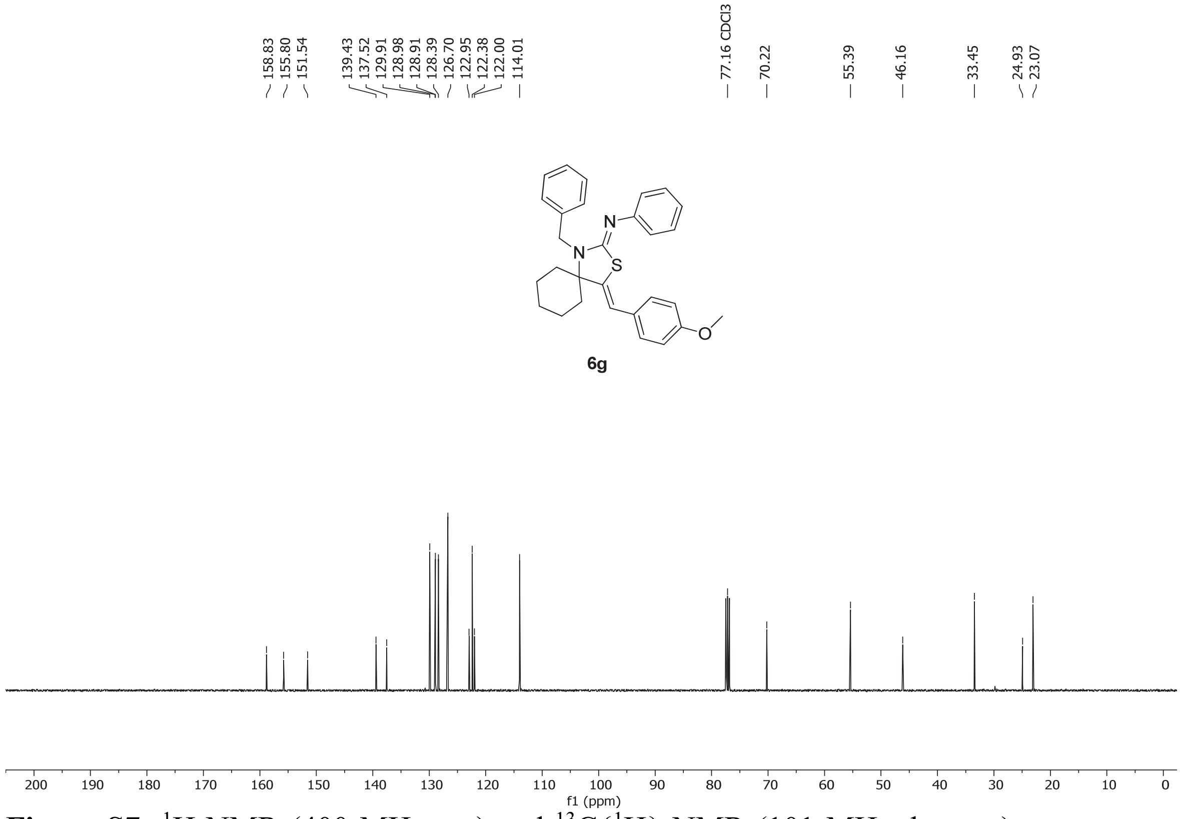

Figure S7: ^{1}H-NMR (400 MHz, up) and ^{13}C{^{1}H}-NMR (101 MHz, bottom) spectra for **6g** in $CDCl_3$.

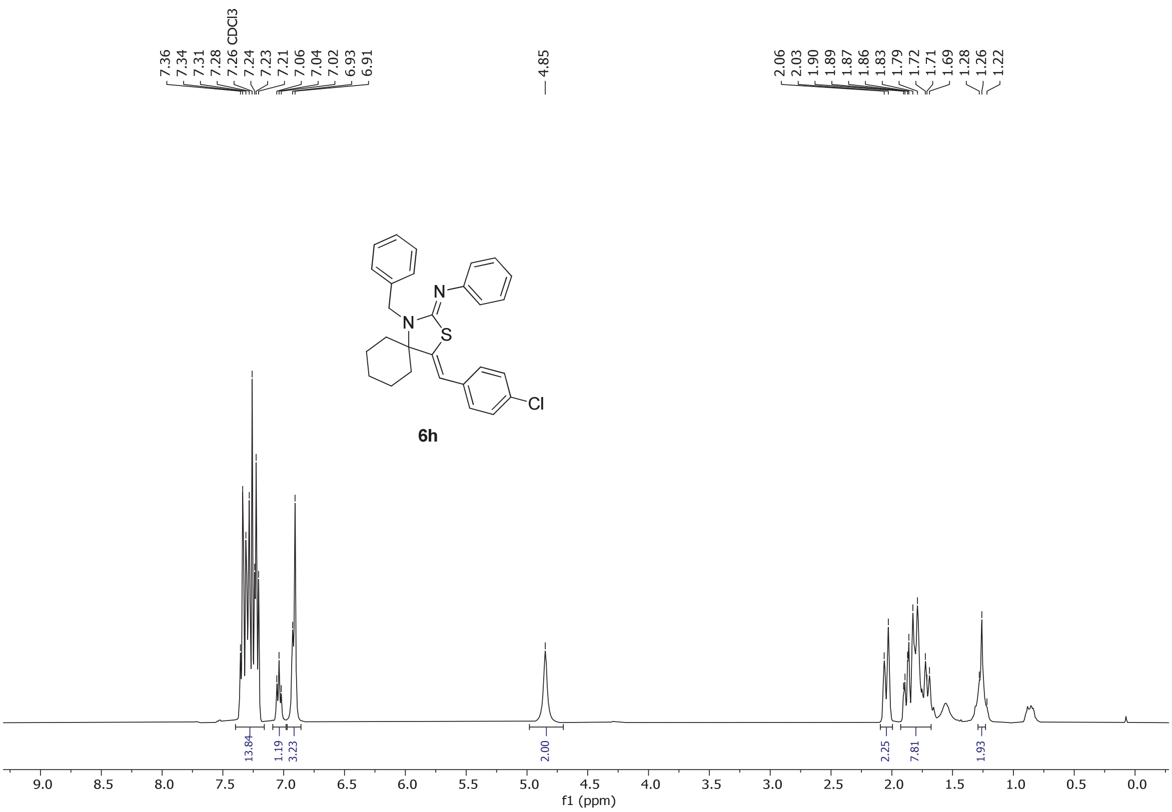

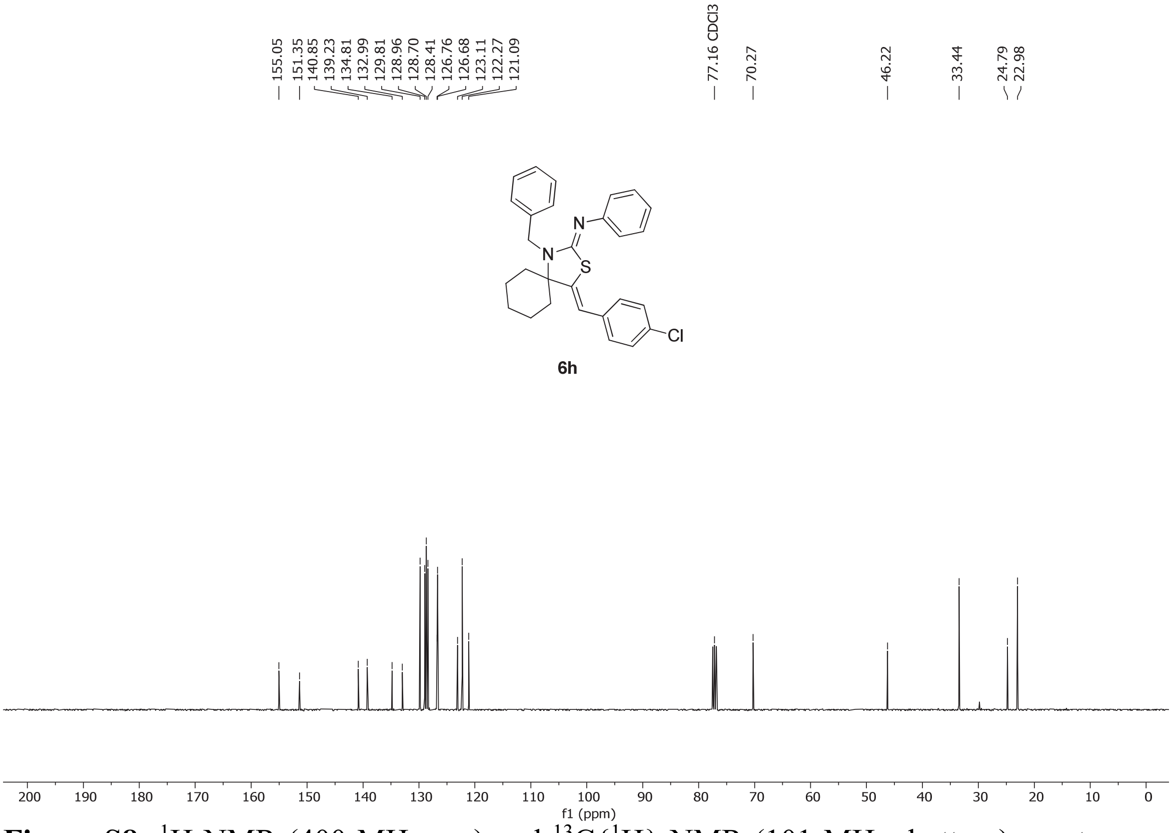

Figure S8: ^{1}H-NMR (400 MHz, up) and ^{13}C{^{1}H}-NMR (101 MHz, bottom) spectra for **6h** in $CDCl_3$.

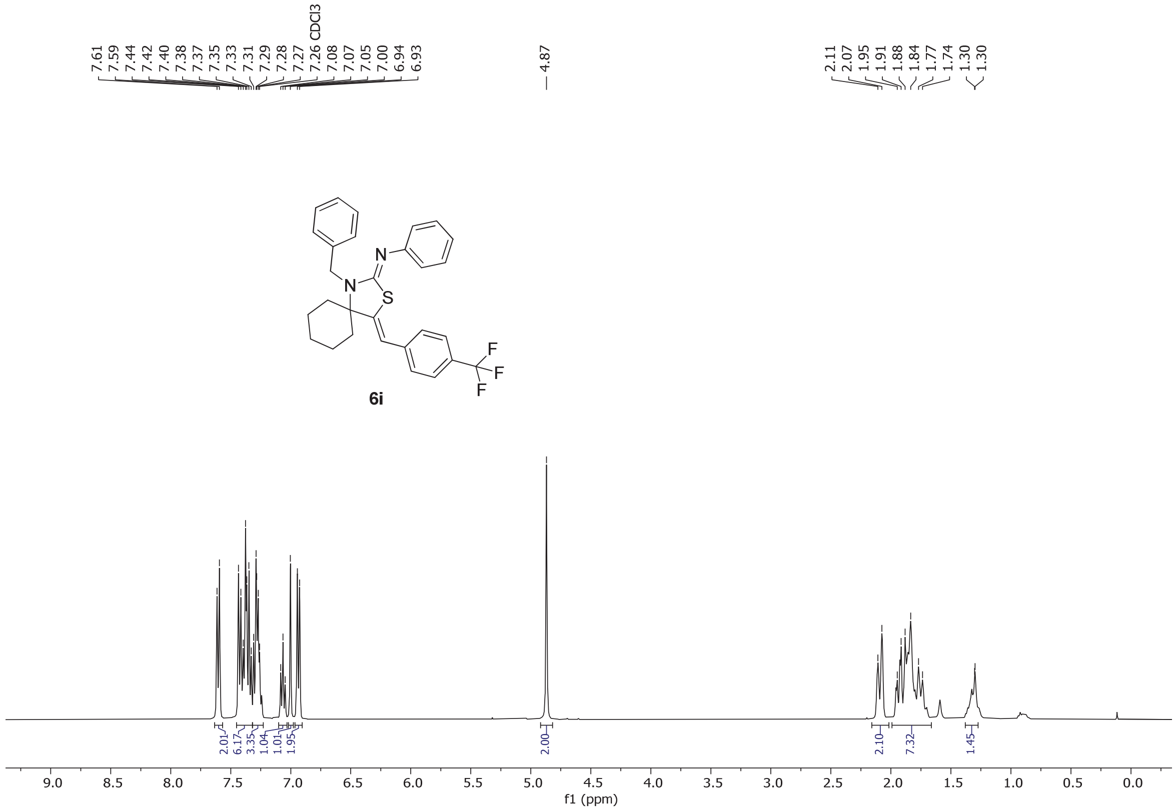
7.61
7.59
7.44
7.42
7.40
7.38
7.37
7.35
7.33
7.31
7.29
7.28
7.27
7.26 CDCl3
7.08
7.07
7.05
7.00
6.94
6.93
4.87
2.11
2.07
1.95
1.91
1.88
1.84
1.77
1.74
1.30
1.30
6i
2.01
6.17
3.35
1.04
1.01
1.95
2.00
2.10
7.32
1.45
f1 (ppm)

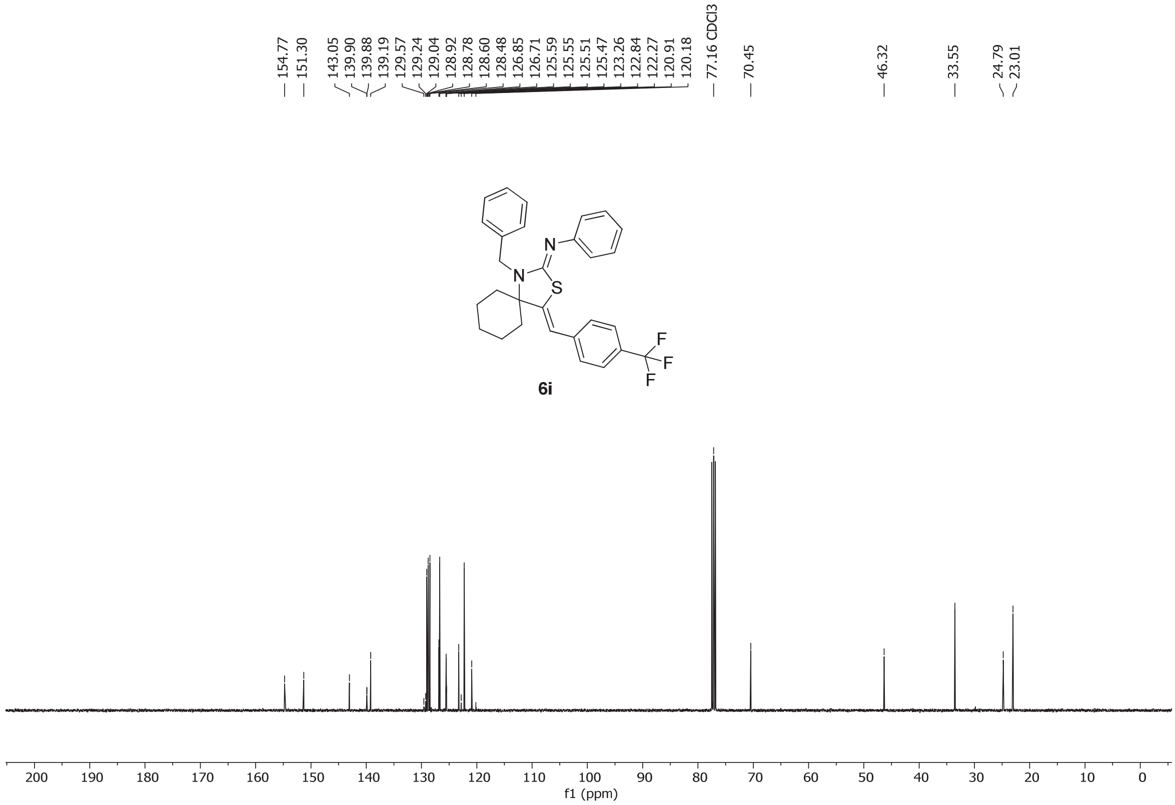
154.77
151.30
143.05
139.90
139.88
139.19
129.57
129.24
129.04
128.92
128.78
128.60
128.48
126.85
126.71
125.59
125.55
125.51
125.47
123.26
122.84
122.27
120.91
120.18
77.16 CDCl3
70.45
46.32
33.55
24.79
23.01
6i
f1 (ppm)

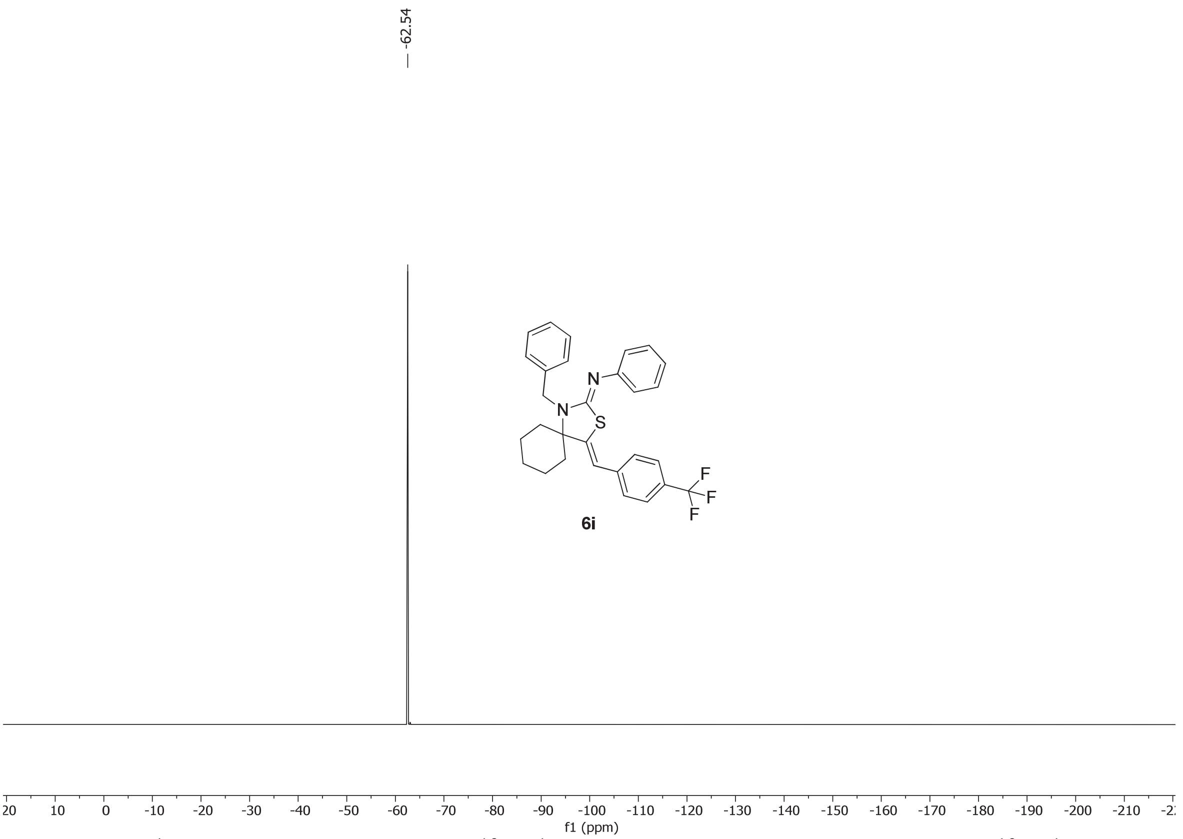

Figure S9: ^{1}H-NMR (400 MHz, up),^{13}C{^{1}H}-NMR (101 MHz, middle), and ^{19}F{^{1}H}-NMR (376 MHz, bottom) spectra for **6i** in $CDCl_3$.

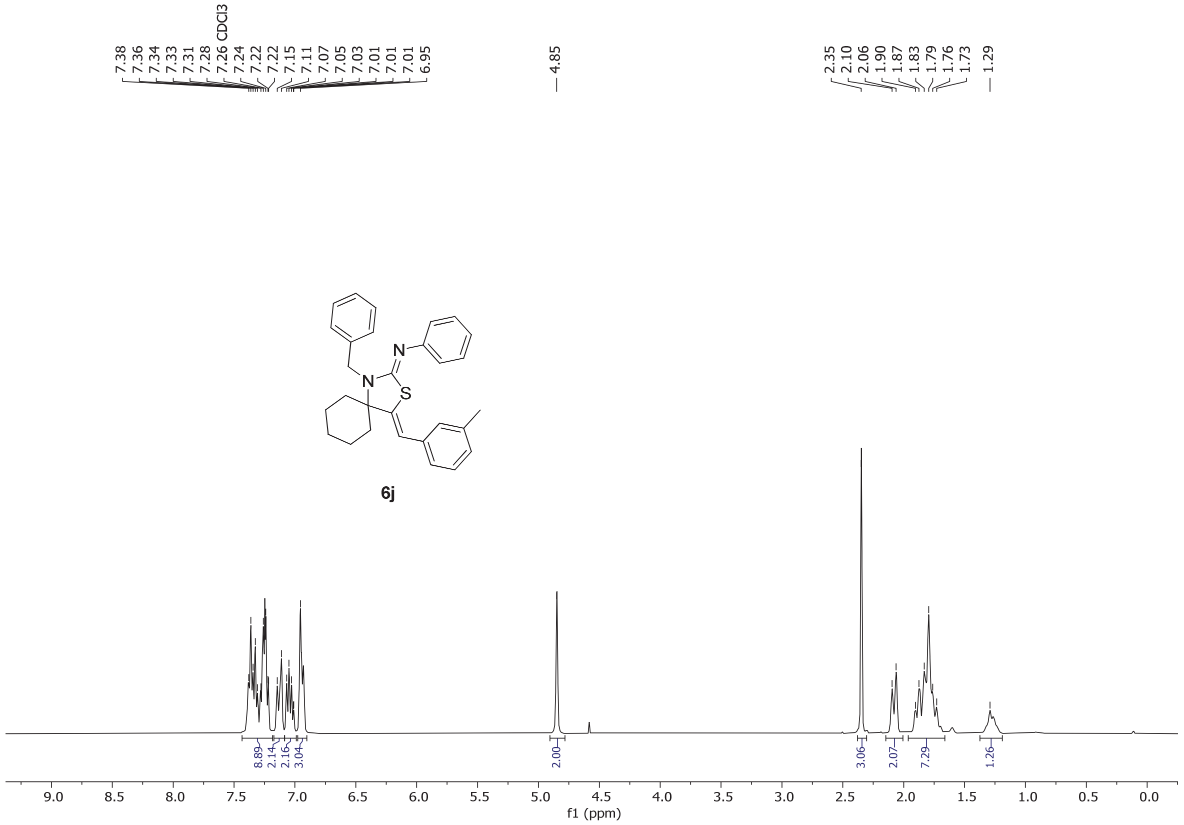

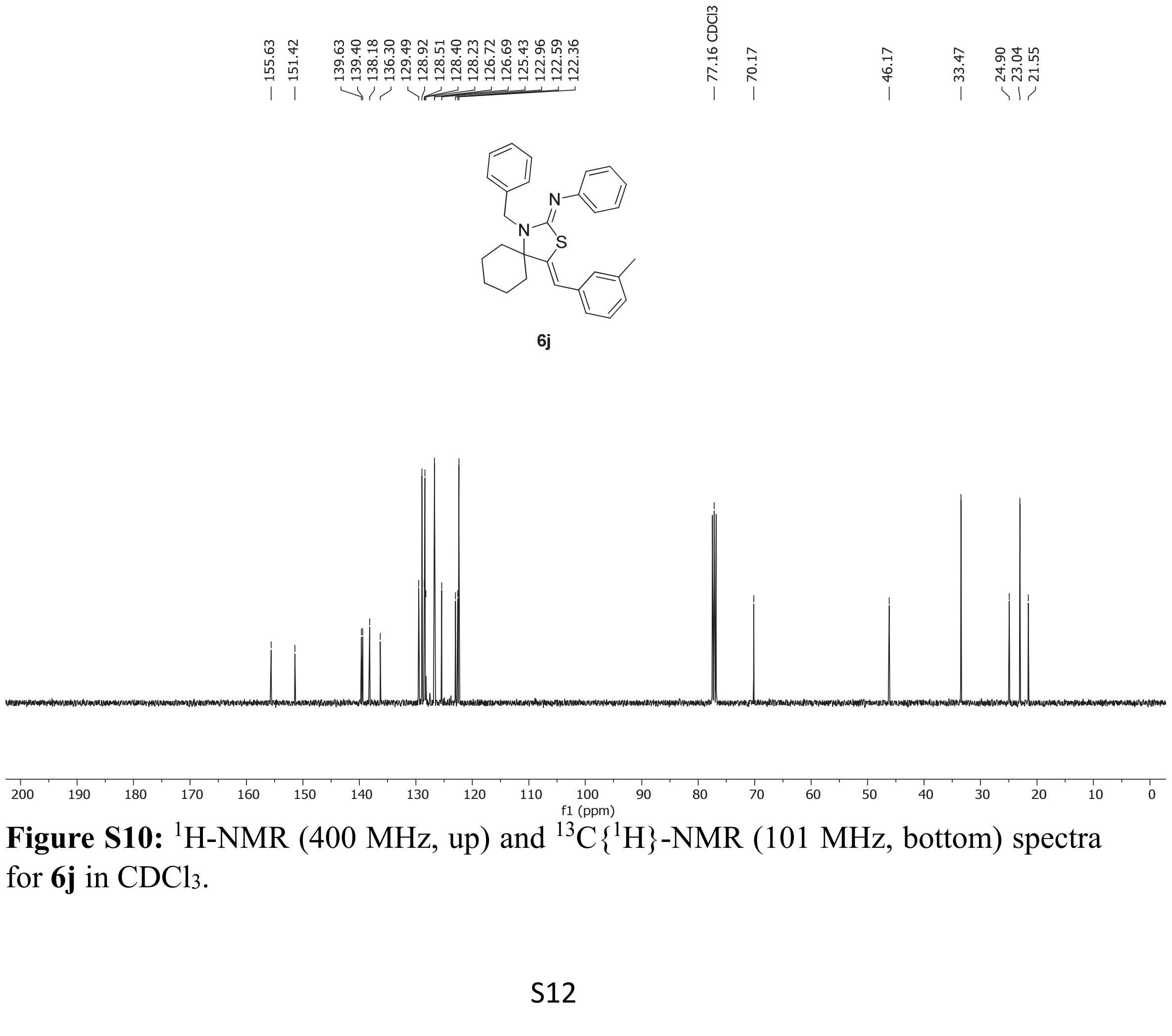

Figure S10: ^{1}H-NMR (400 MHz, up) and ^{13}C{^{1}H}-NMR (101 MHz, bottom) spectra for **6j** in $CDCl_3$.

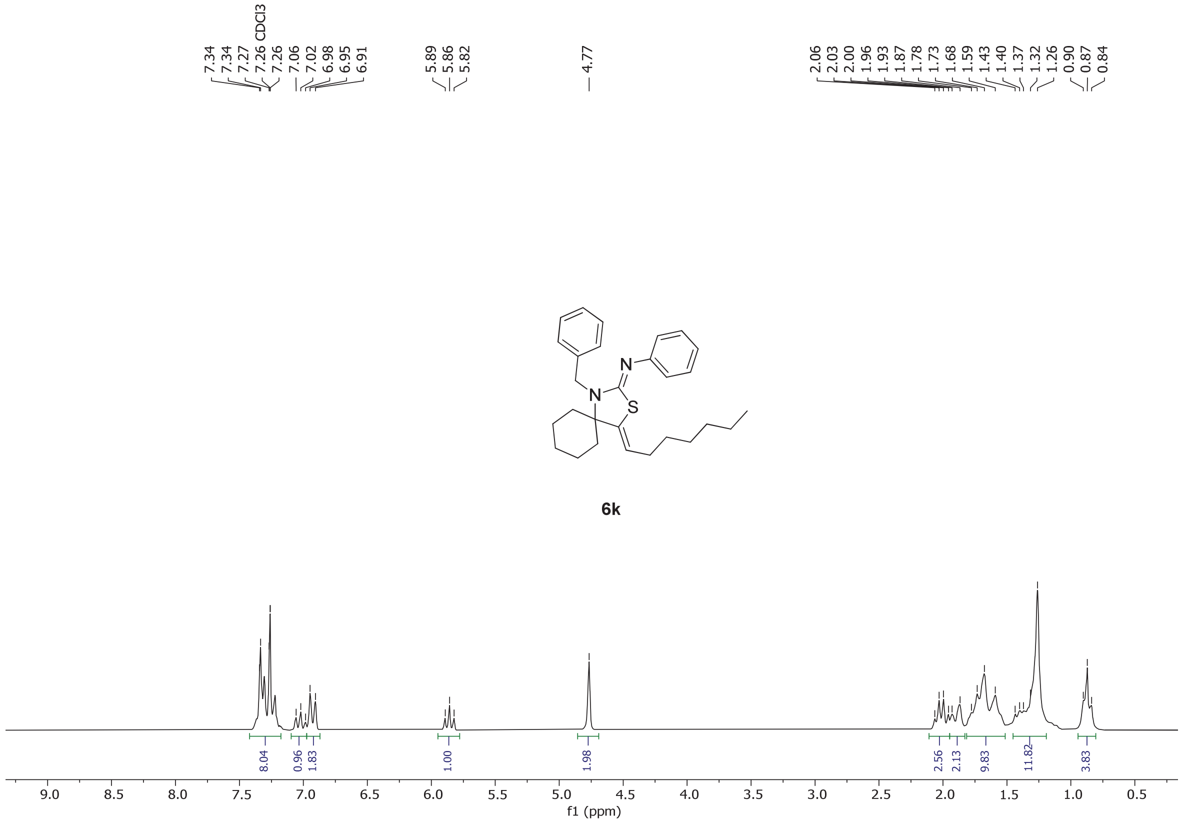

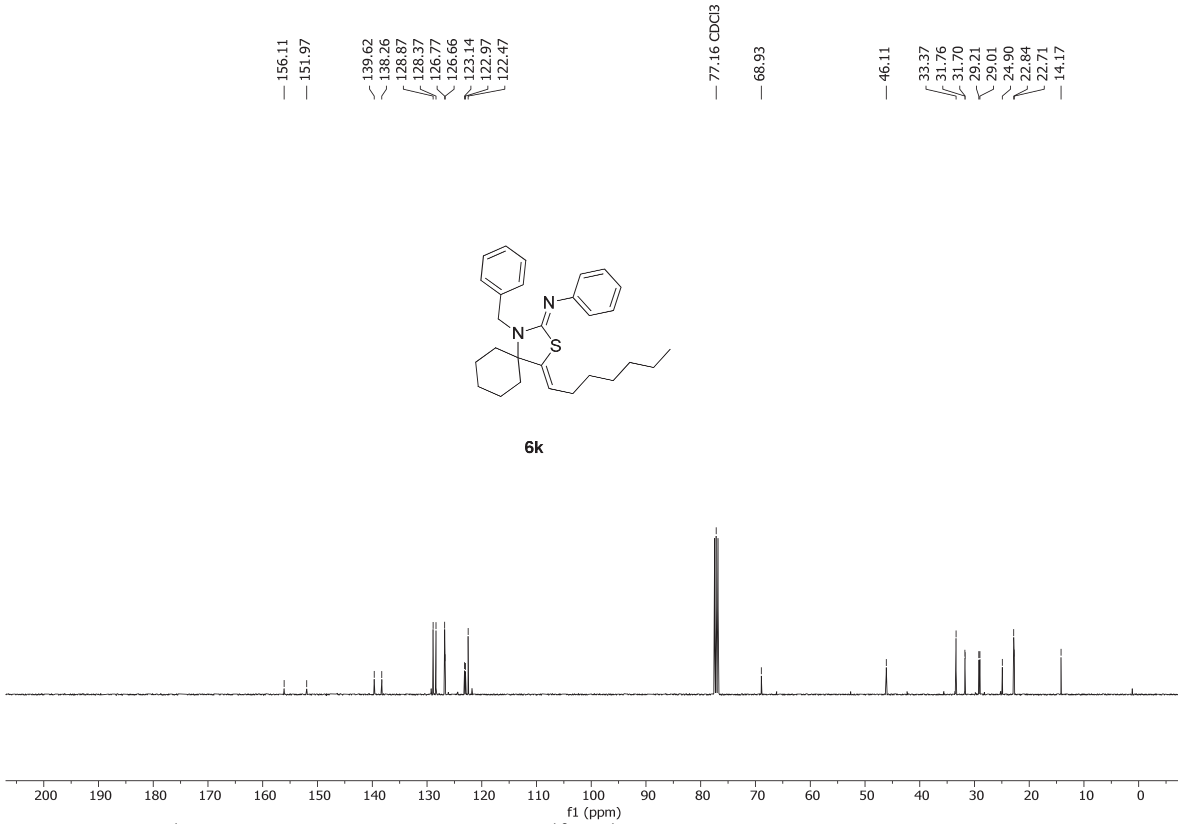

Figure S11: ^{1}H-NMR (200 MHz, up) and ^{13}C{^{1}H}-NMR (101 MHz, bottom) spectra for **6k** in $CDCl_3$.

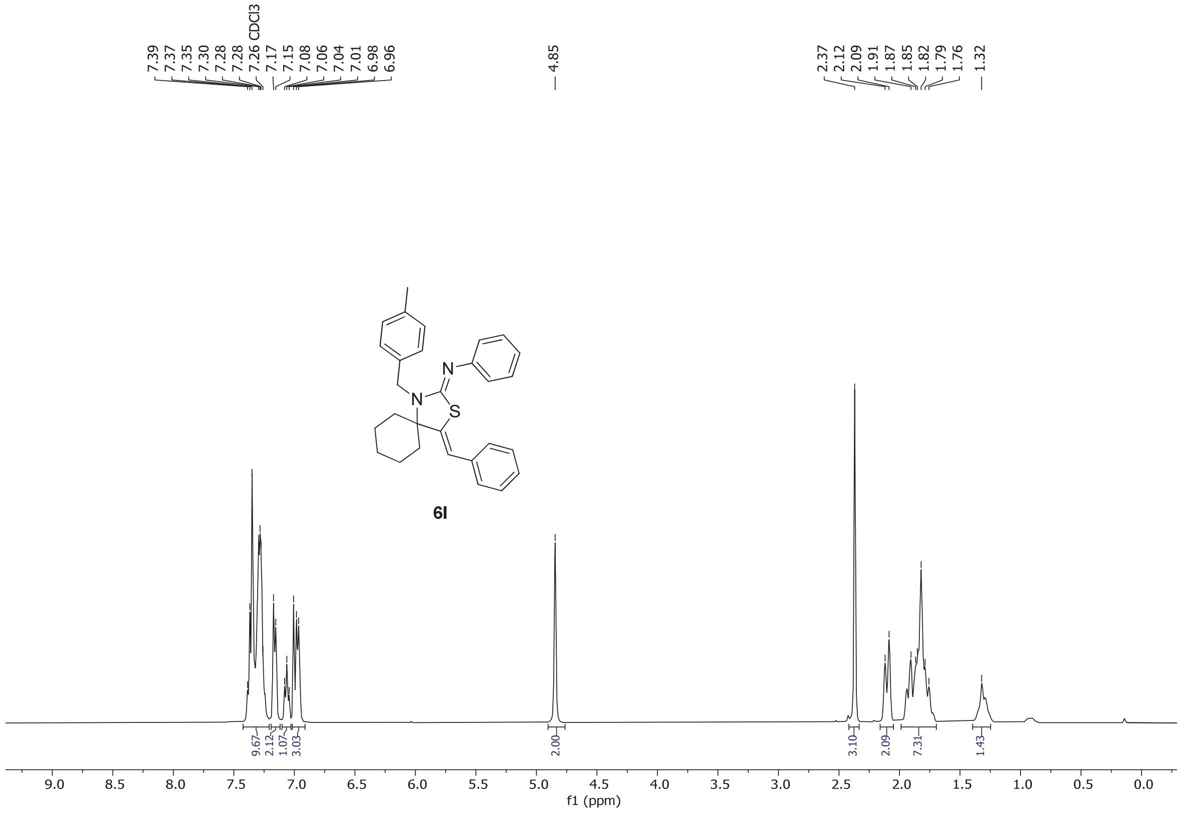

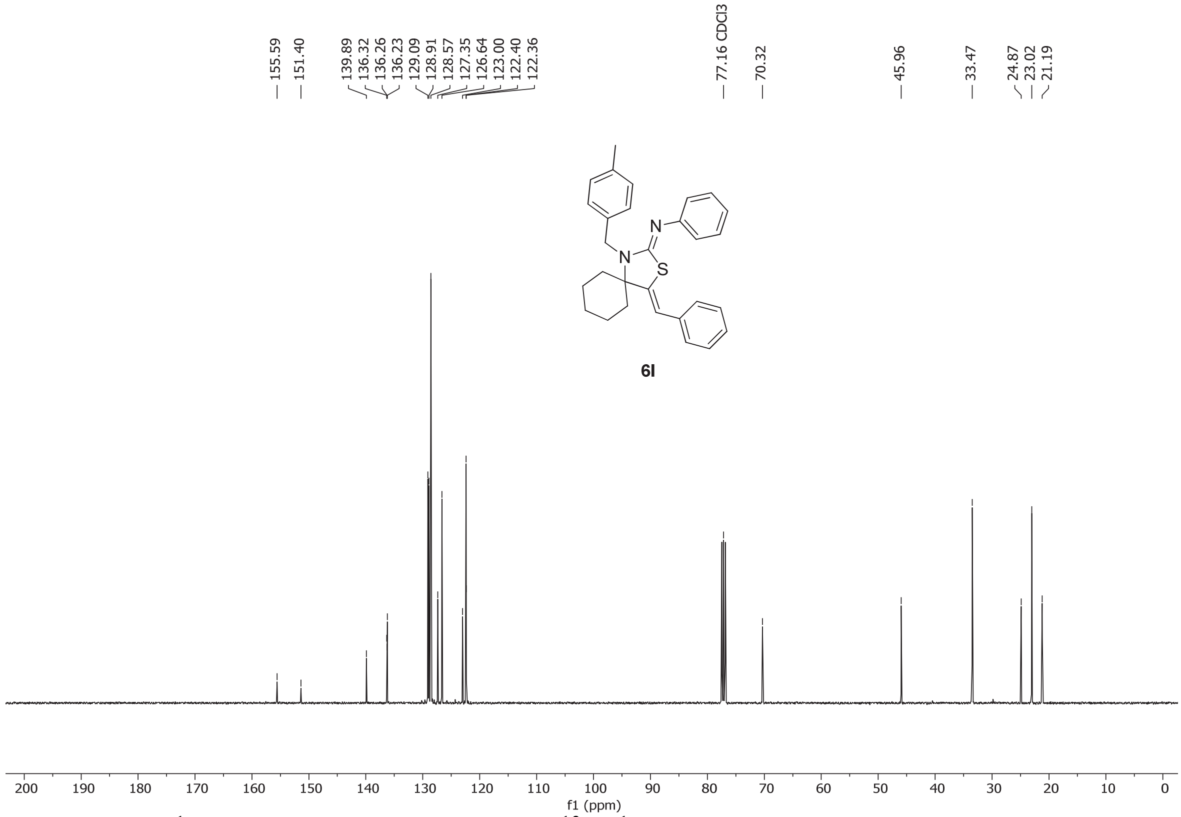

Figure S12: ^{1}H-NMR (400 MHz, up) and ^{13}C{^{1}H}-NMR (101 MHz, bottom) spectra for **6l** in $CDCl_3$.

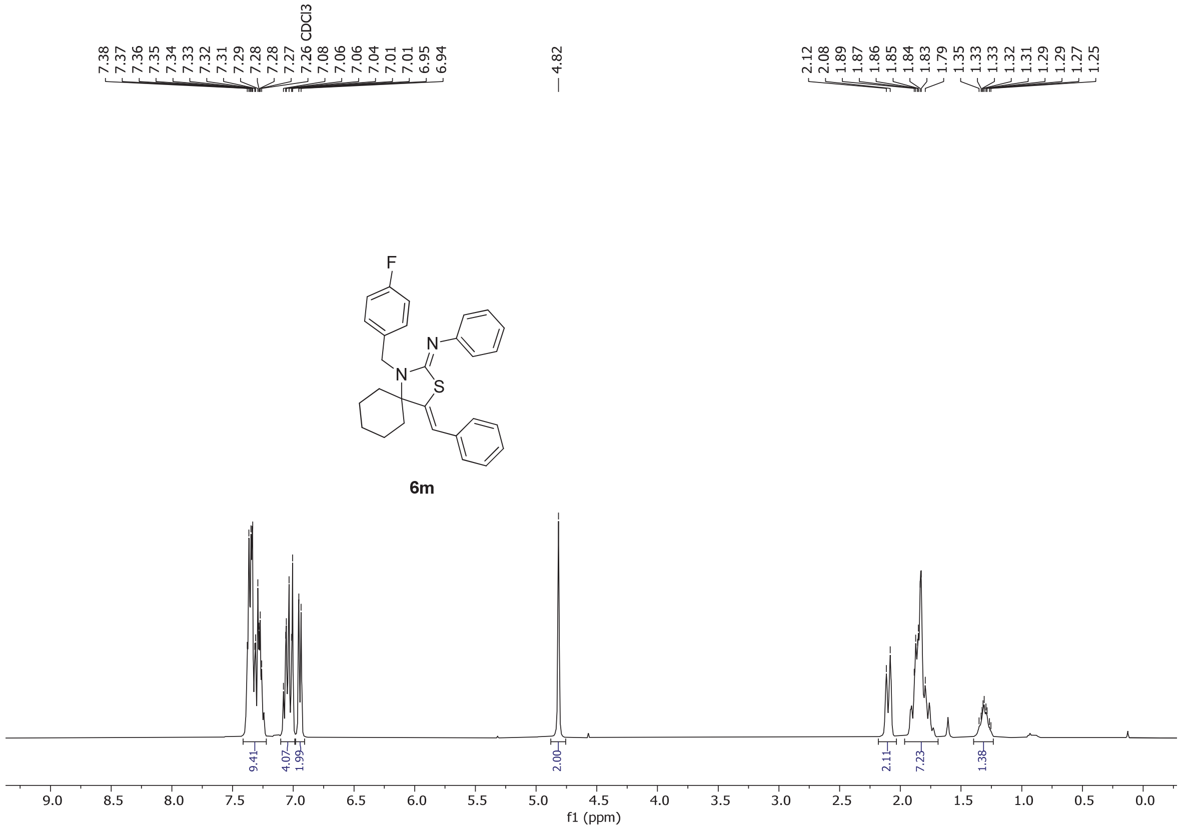
7.38 7.37 7.36 7.35 7.34 7.33 7.32 7.31 7.29 7.28 7.28 7.27 7.26 CDCl3 7.08 7.06 7.06 7.04 7.01 6.95 6.94
4.82
2.12 2.08 1.89 1.87 1.86 1.85 1.84 1.83 1.79 1.35 1.33 1.33 1.32 1.31 1.29 1.29 1.27 1.25
F
N
S
6m
9.41
4.07
1.99
2.00
2.11
7.23
1.38
f1 (ppm)

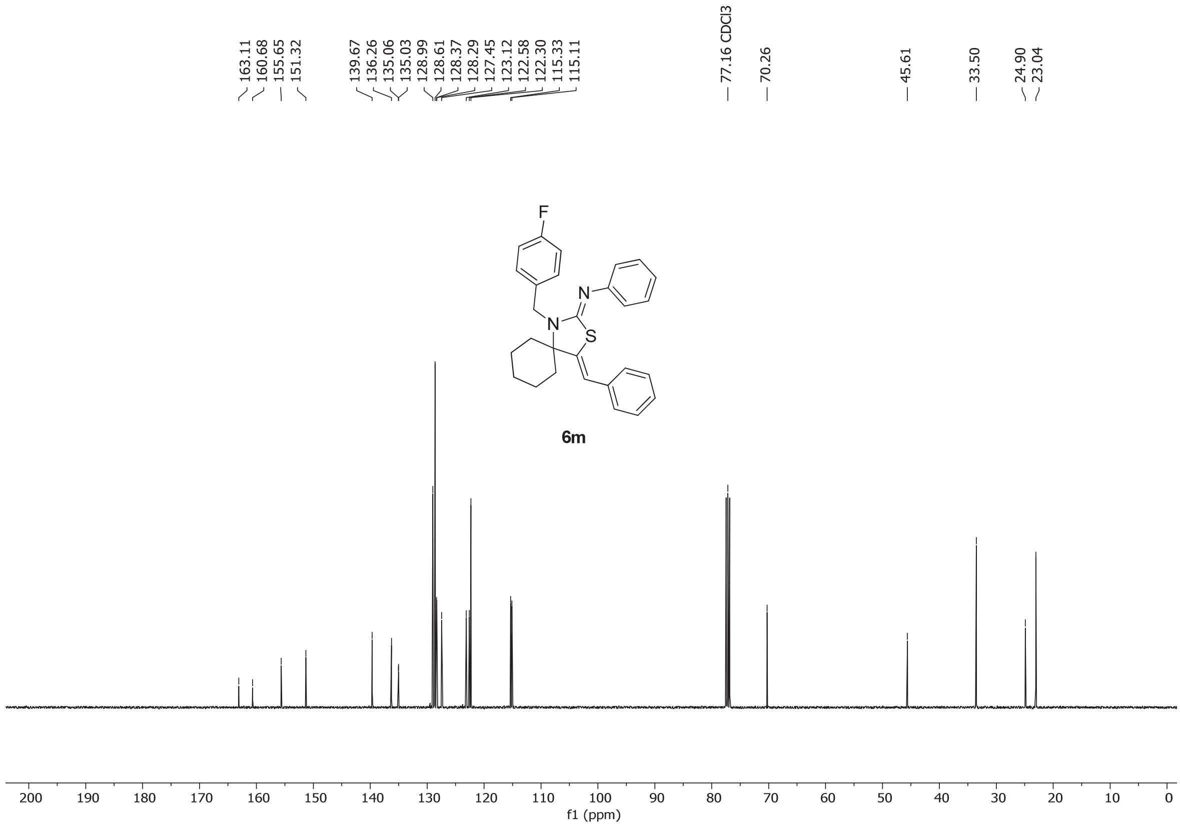
163.11 160.68 155.65 151.32
139.67 136.26 135.06 135.03 128.99 128.61 128.37 128.29 127.45 123.12 122.58 122.30 115.33 115.11
77.16 CDCl3
70.26
45.61
33.50
24.90 23.04
F
N
S
6m
f1 (ppm)

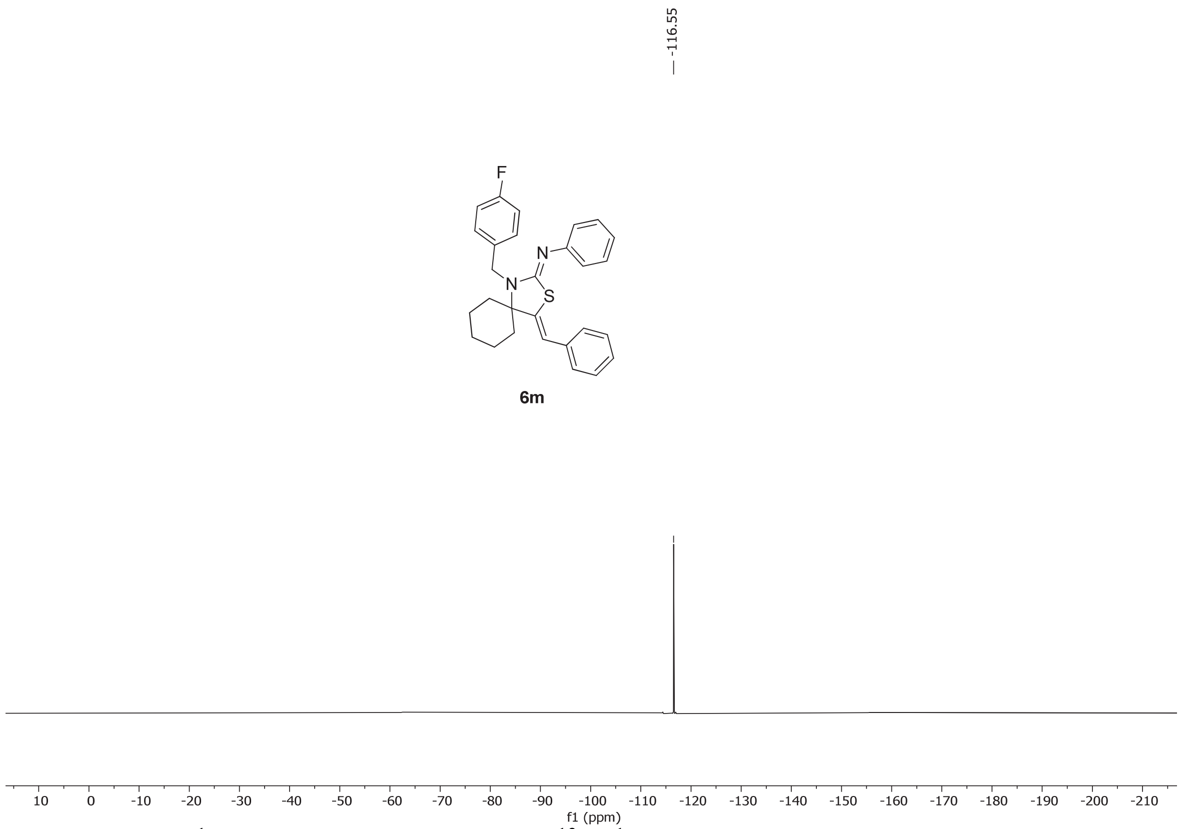

Figure S13: ^{1}H-NMR (400 MHz, up),^{13}C{^{1}H}-NMR (101 MHz, middle), and ^{19}F{^{1}H}-NMR (376 MHz, bottom) spectra for **6m** in $CDCl_3$.

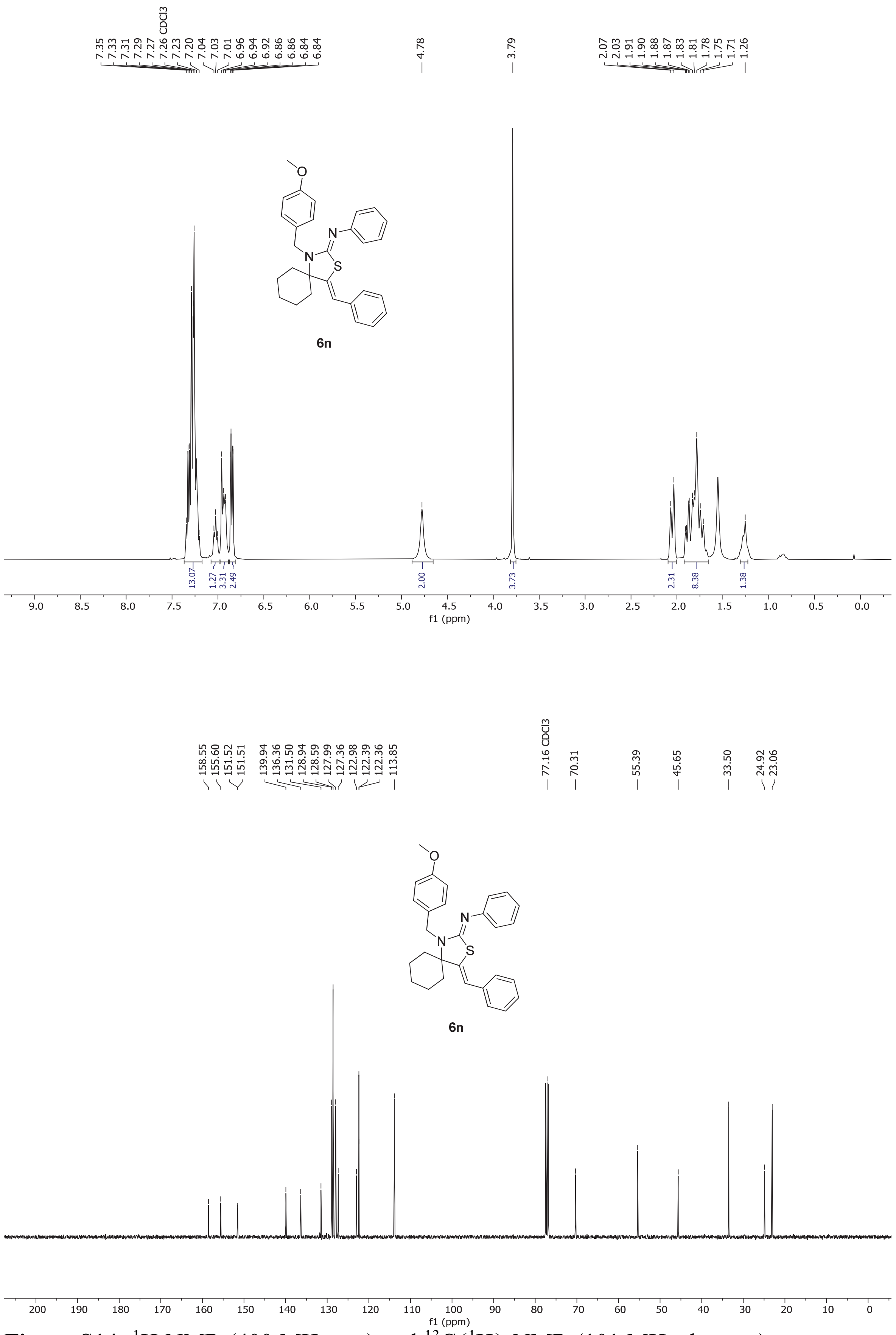

Figure S14: ^{1}H-NMR (400 MHz, up) and ^{13}C{^{1}H}-NMR (101 MHz, bottom) spectra for **6n** in $CDCl_3$.

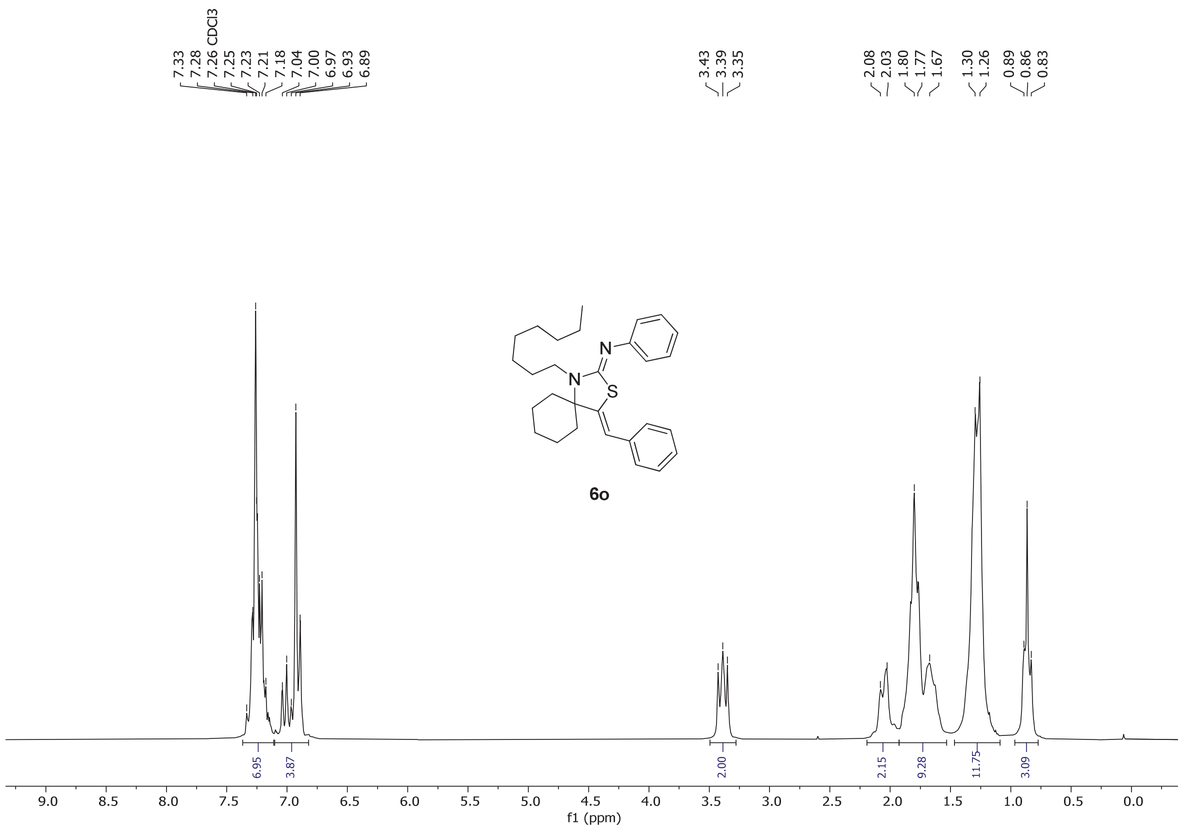

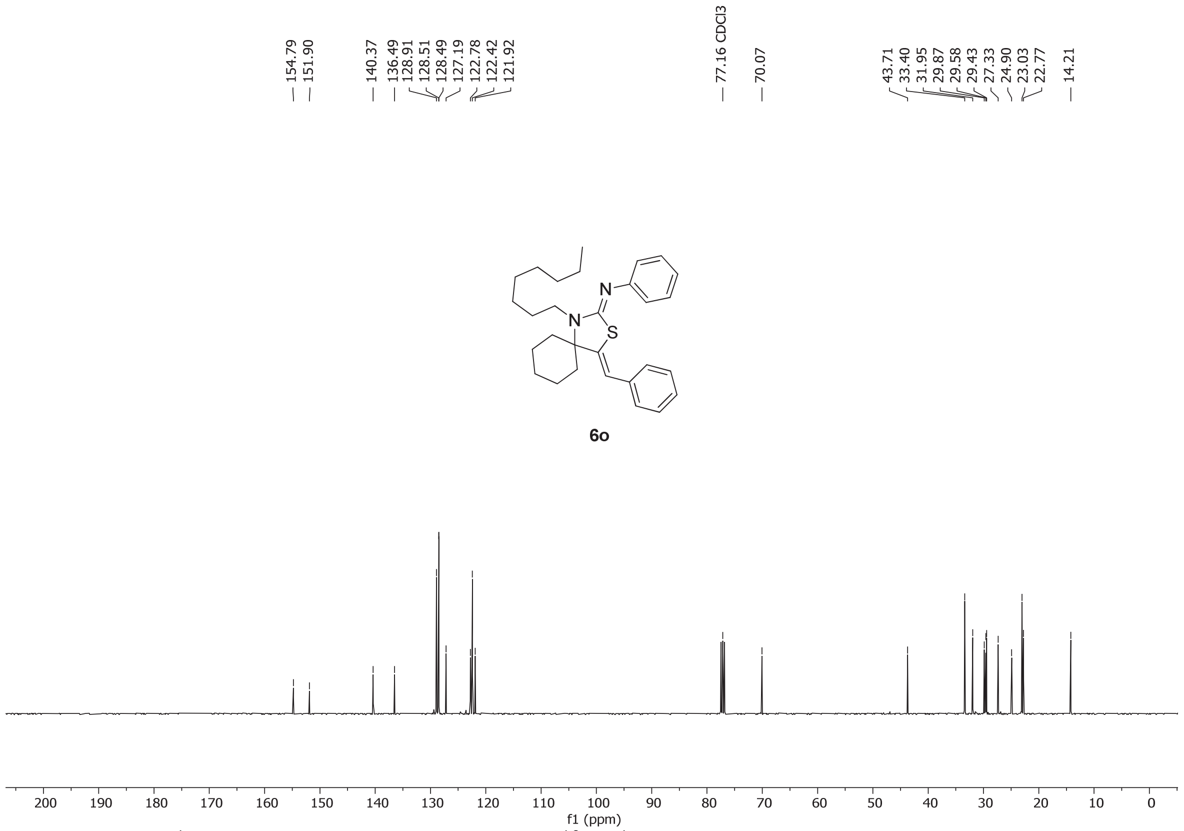

Figure S15: ^{1}H-NMR (200 MHz, up) and ^{13}C{^{1}H}-NMR (101 MHz, bottom) spectra for **6o** in $CDCl_3$.

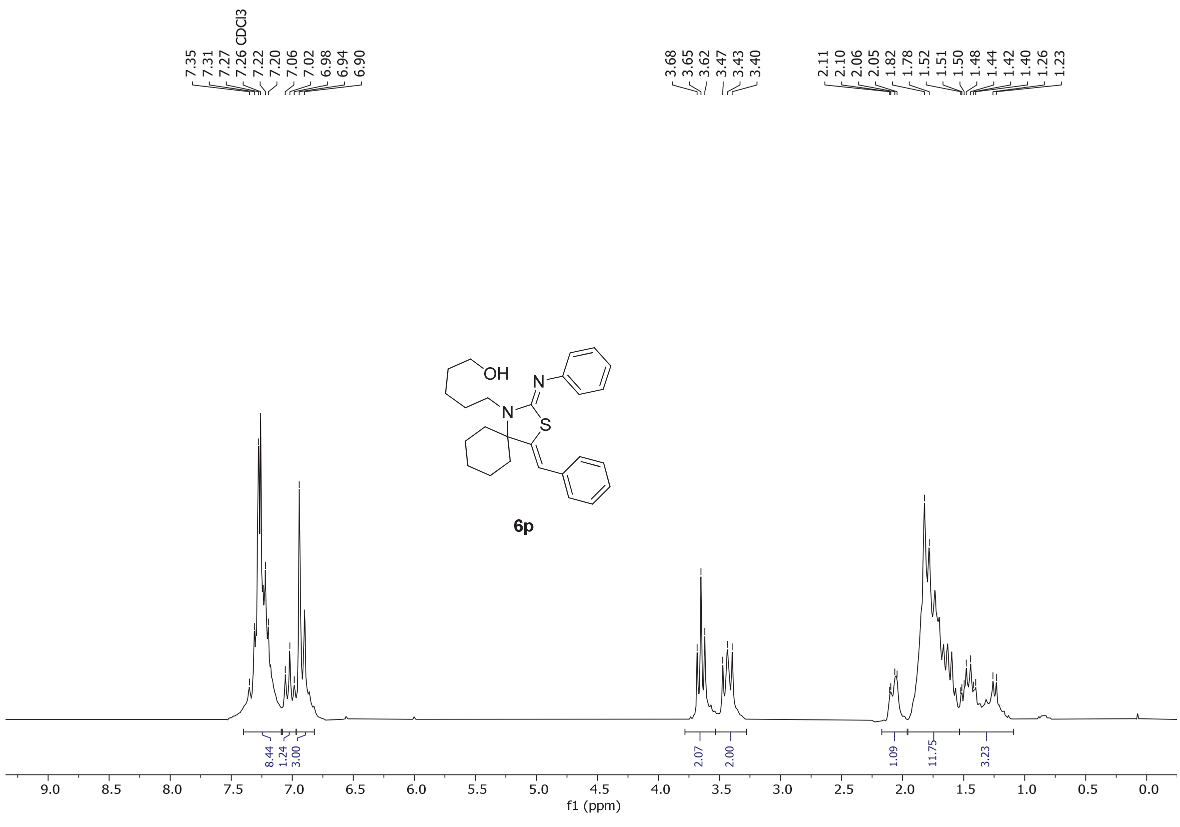

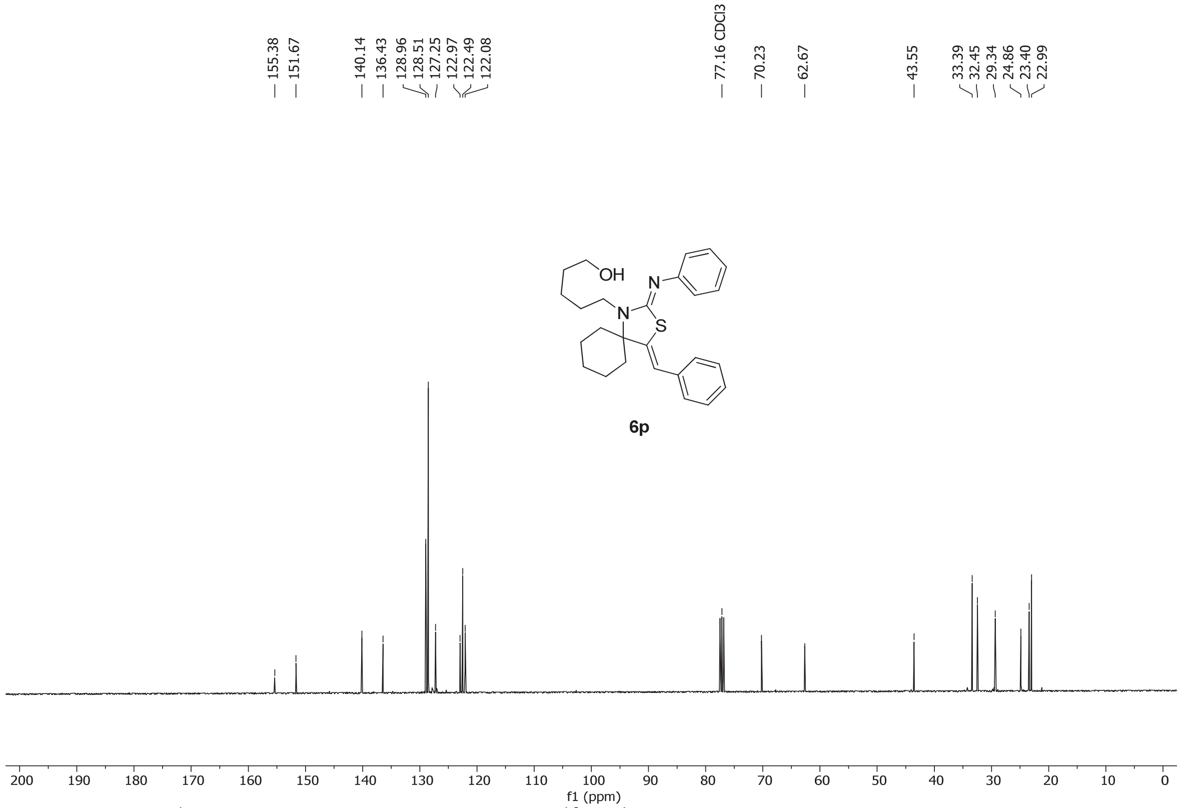

Figure S16: ^{1}H-NMR (200 MHz, up) and ^{13}C{^{1}H}-NMR (101 MHz, bottom) spectra for **6p** in $CDCl_3$.

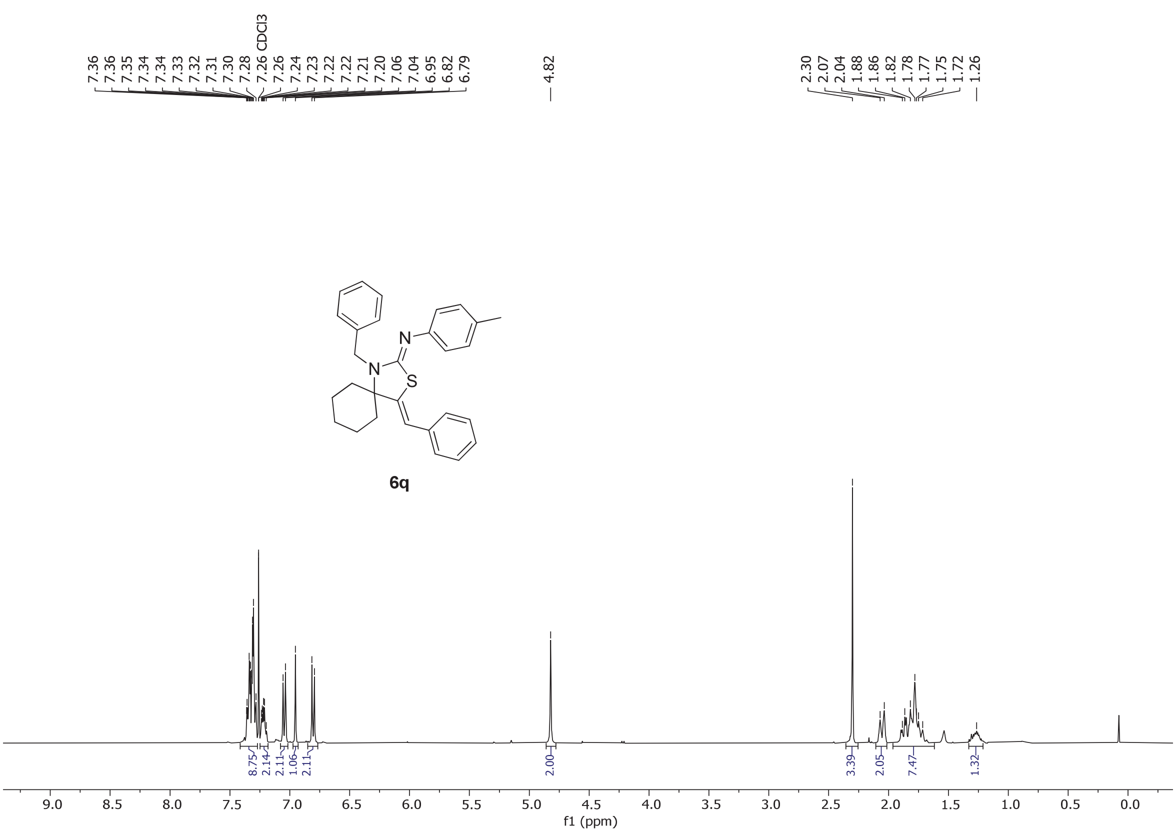

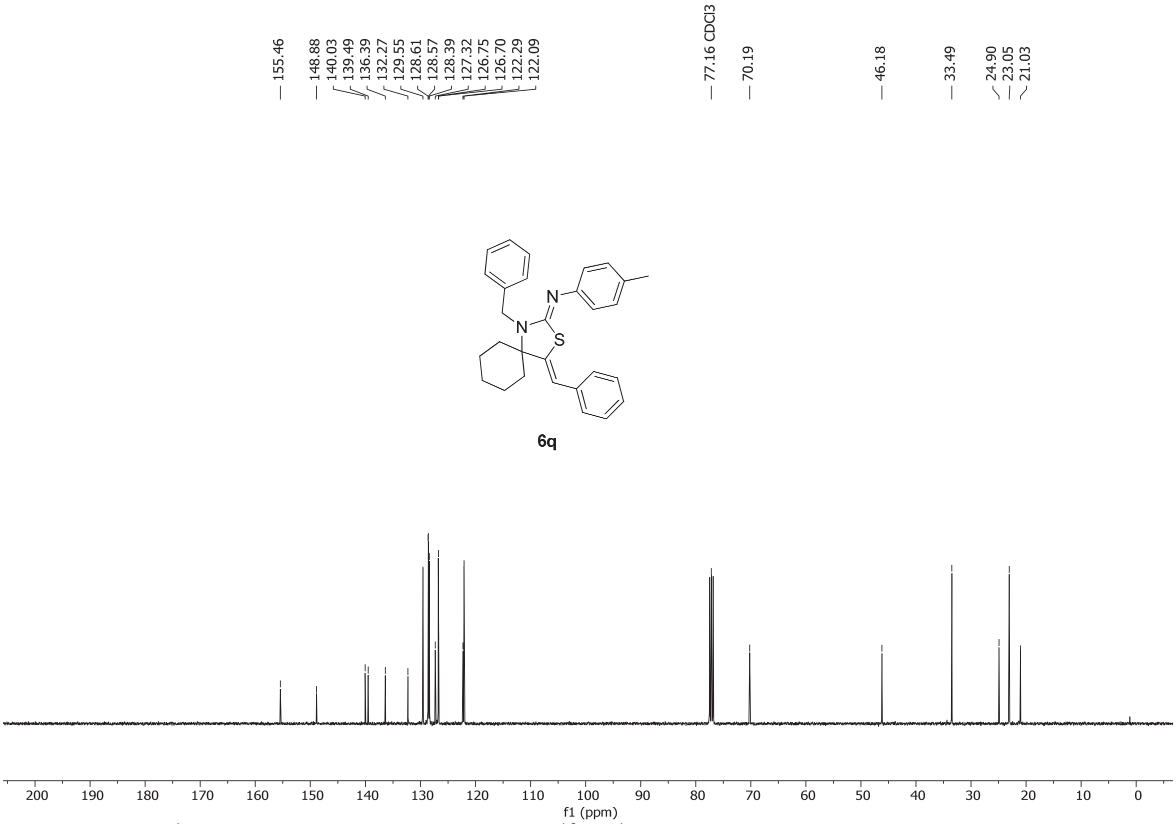

Figure S17: ^{1}H-NMR (400 MHz, up) and ^{13}C{^{1}H}-NMR (101 MHz, bottom) spectra for **6q** in $CDCl_3$.

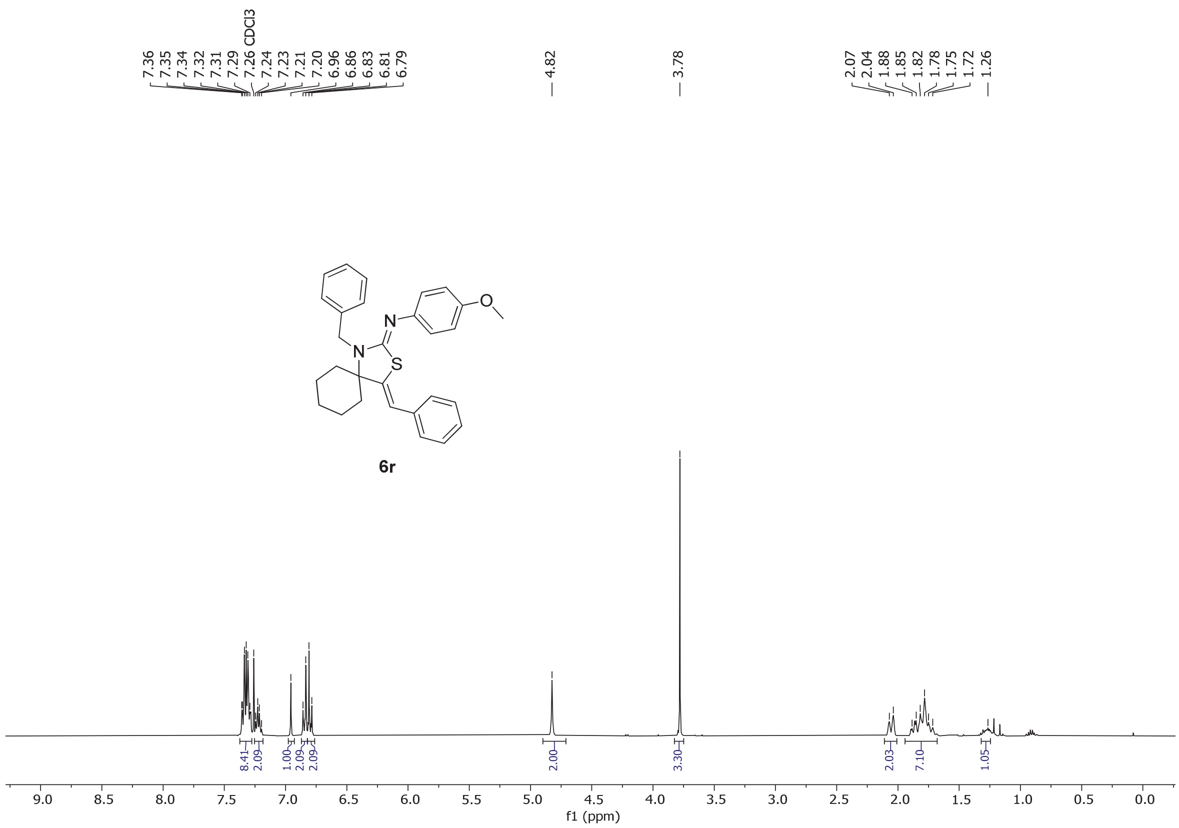

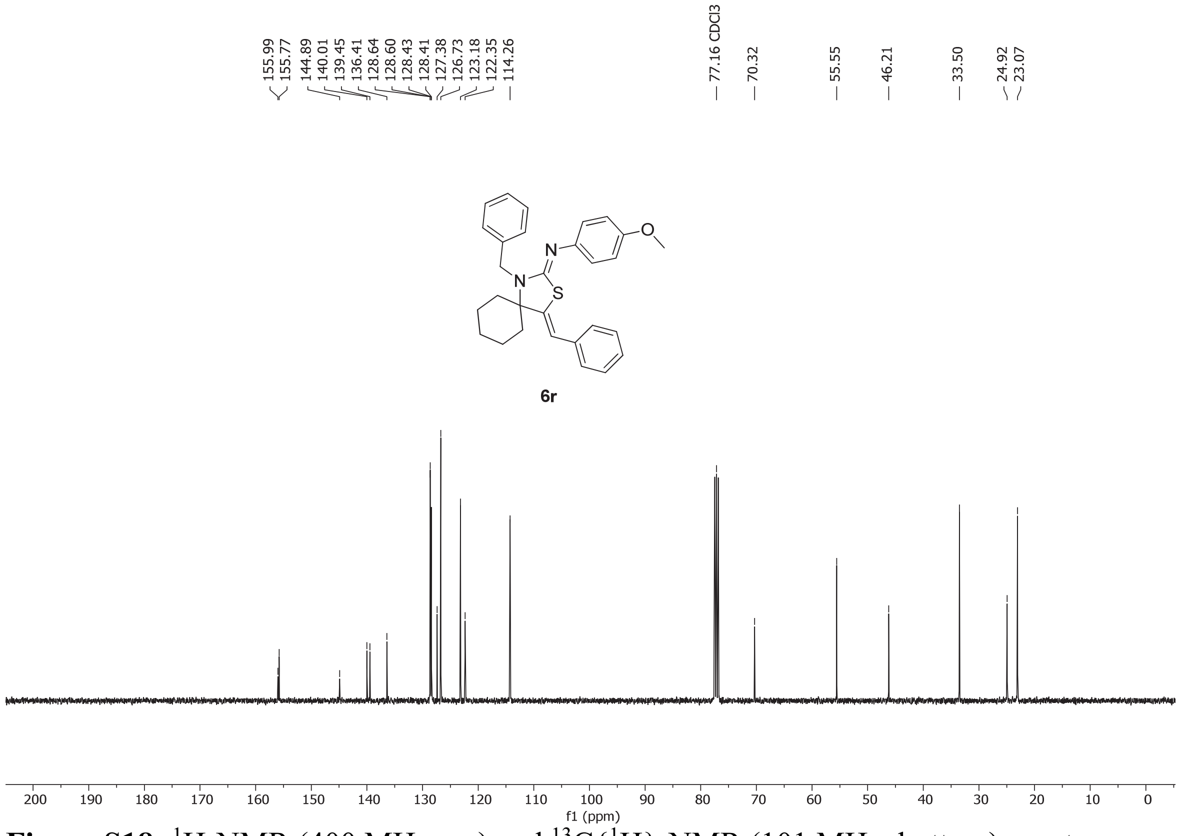

Figure S18: ^{1}H-NMR (400 MHz, up) and $^{13}C\{^{1}H\}$-NMR (101 MHz, bottom) spectra for **6r** in $CDCl_3$.

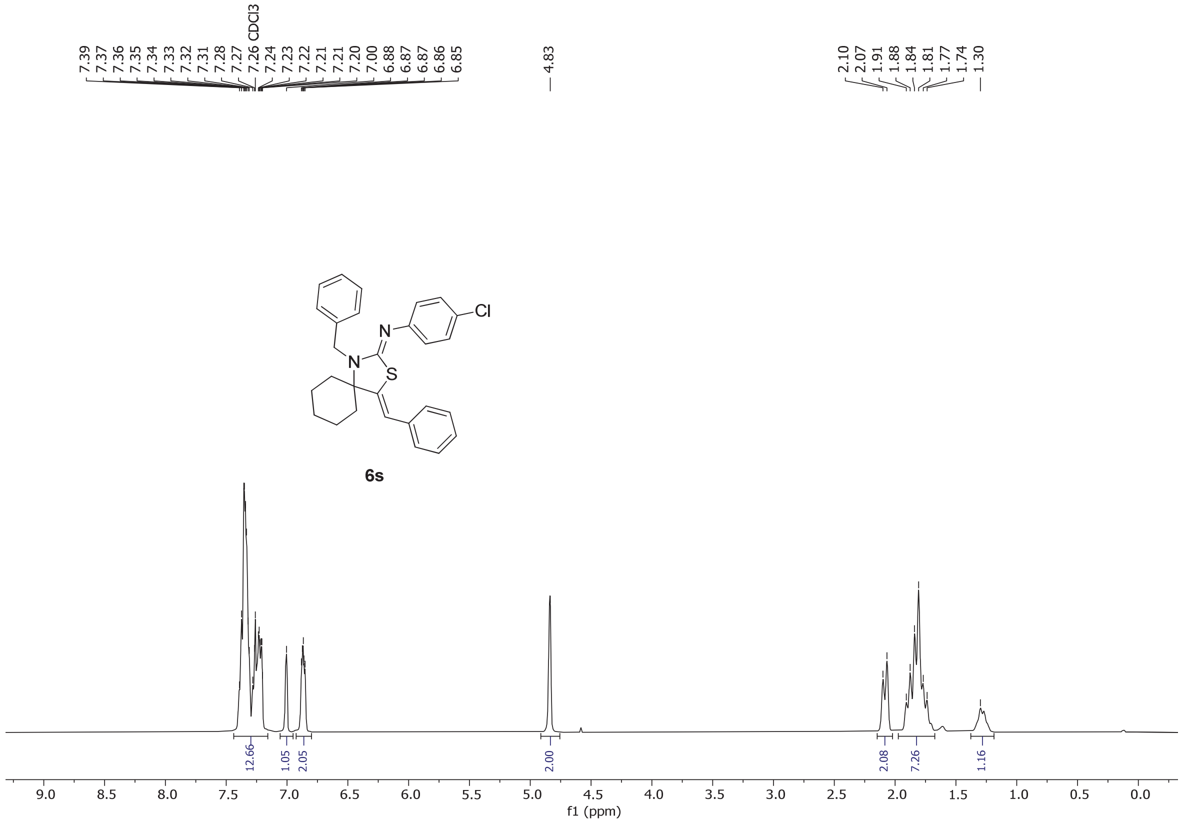

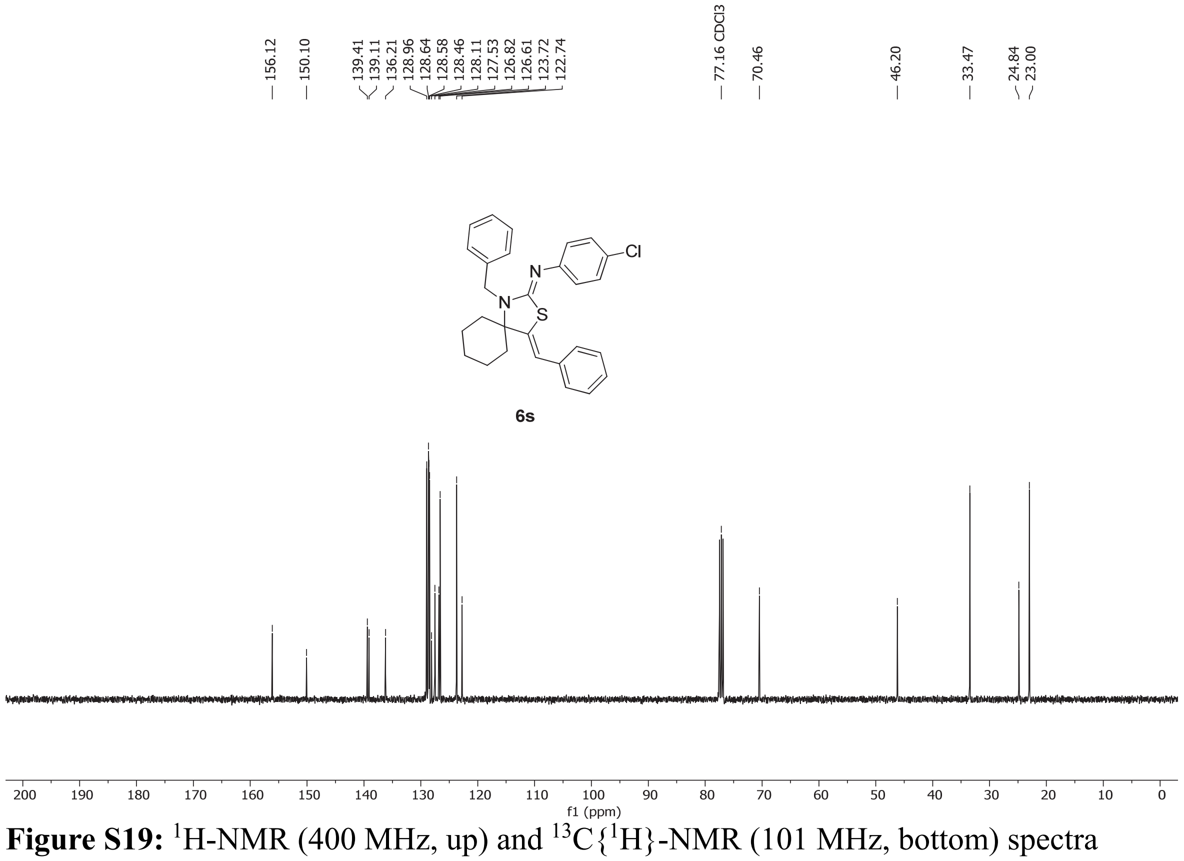

Figure S19: ^{1}H-NMR (400 MHz, up) and ^{13}C{^{1}H}-NMR (101 MHz, bottom) spectra for **6s** in $CDCl_3$.

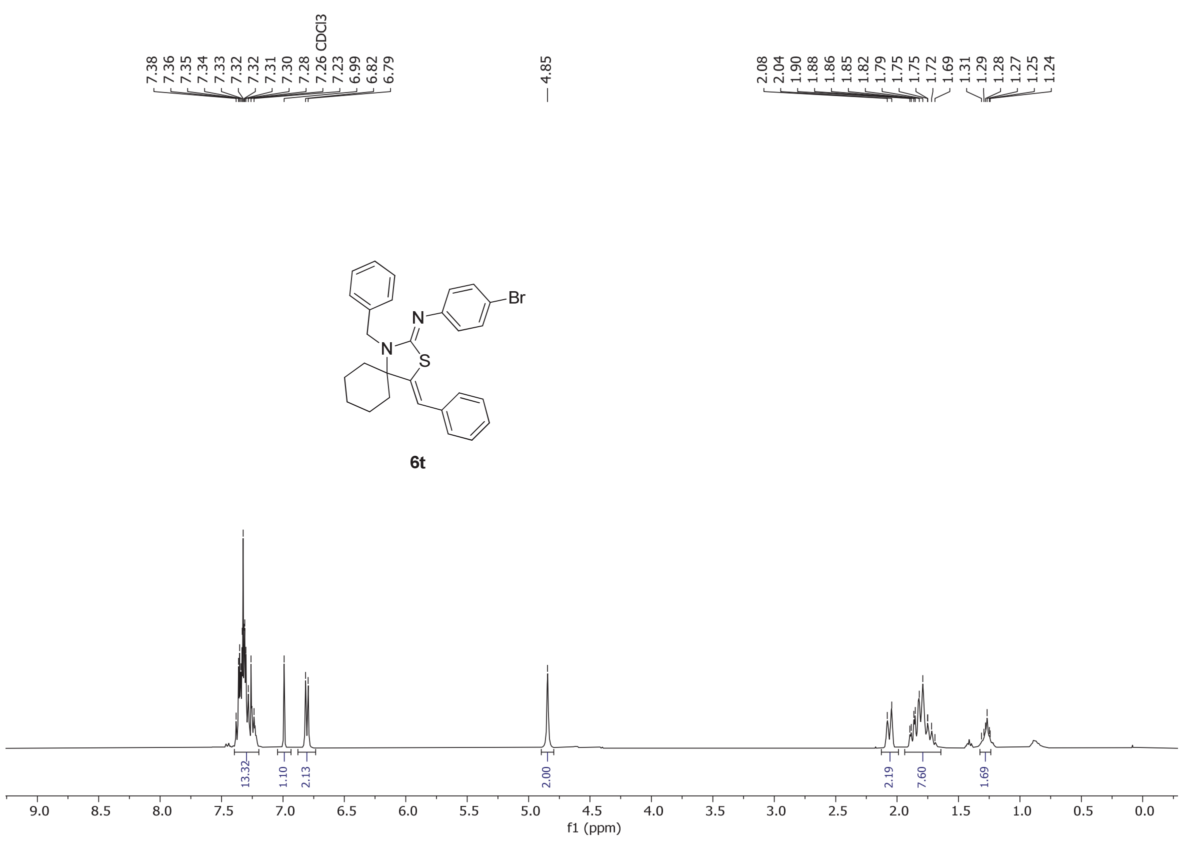

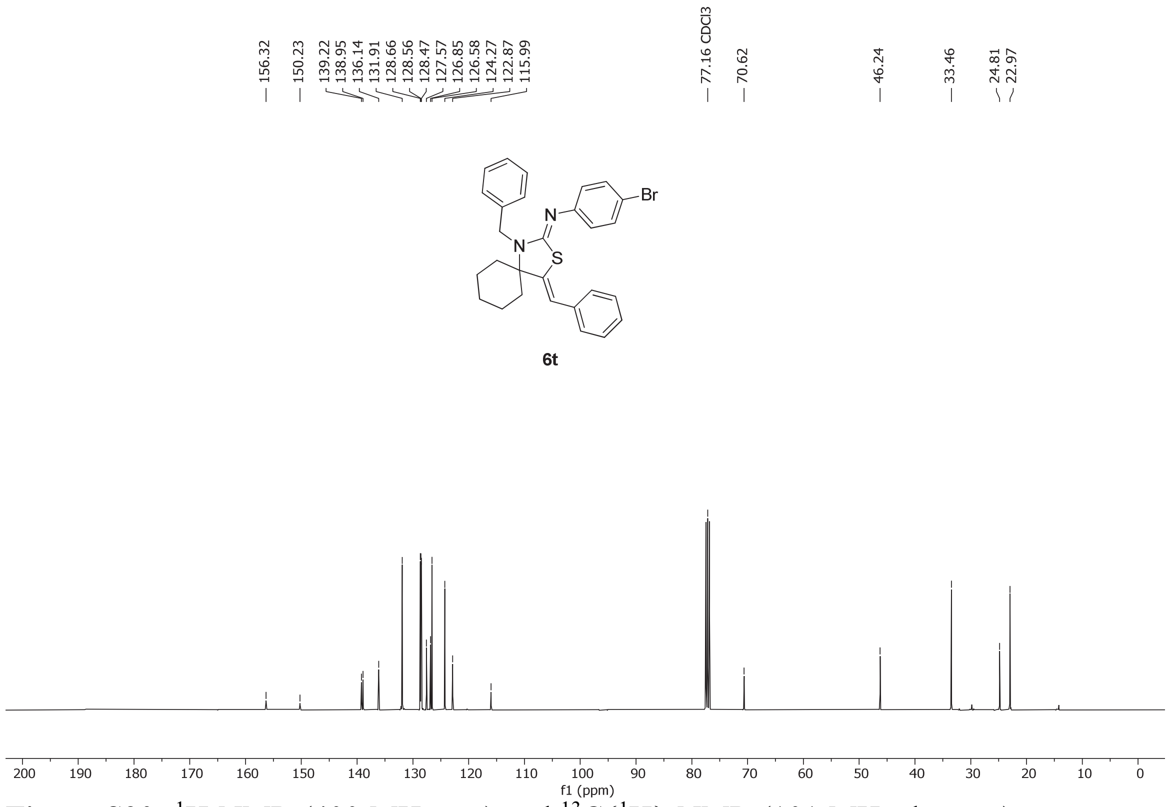

Figure S20: ^{1}H-NMR (400 MHz, up) and $^{13}C\{^{1}H\}$-NMR (101 MHz, bottom) spectra for **6t** in $CDCl_3$.

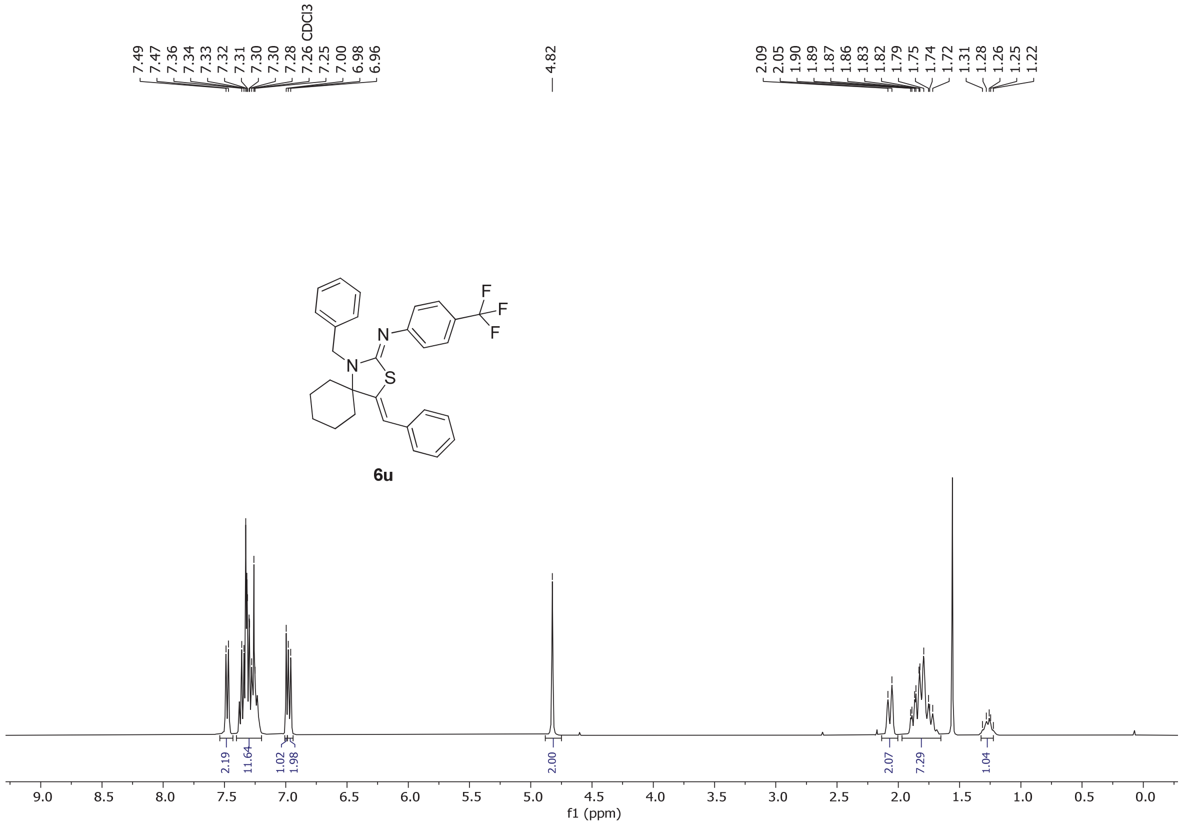

7.49 7.47 7.36 7.34 7.33 7.32 7.31 7.30 7.30 7.28 7.26 CDCl3 7.25 7.00 6.98 6.96
4.82
2.09 2.05 1.90 1.89 1.87 1.86 1.83 1.82 1.79 1.75 1.74 1.72 1.31 1.28 1.26 1.25 1.22
F F F N N S
6u
2.19 11.64 1.02 1.98 2.00 2.07 7.29 1.04
9.0 8.5 8.0 7.5 7.0 6.5 6.0 5.5 5.0 4.5 4.0 3.5 3.0 2.5 2.0 1.5 1.0 0.5 0.0
f1 (ppm)

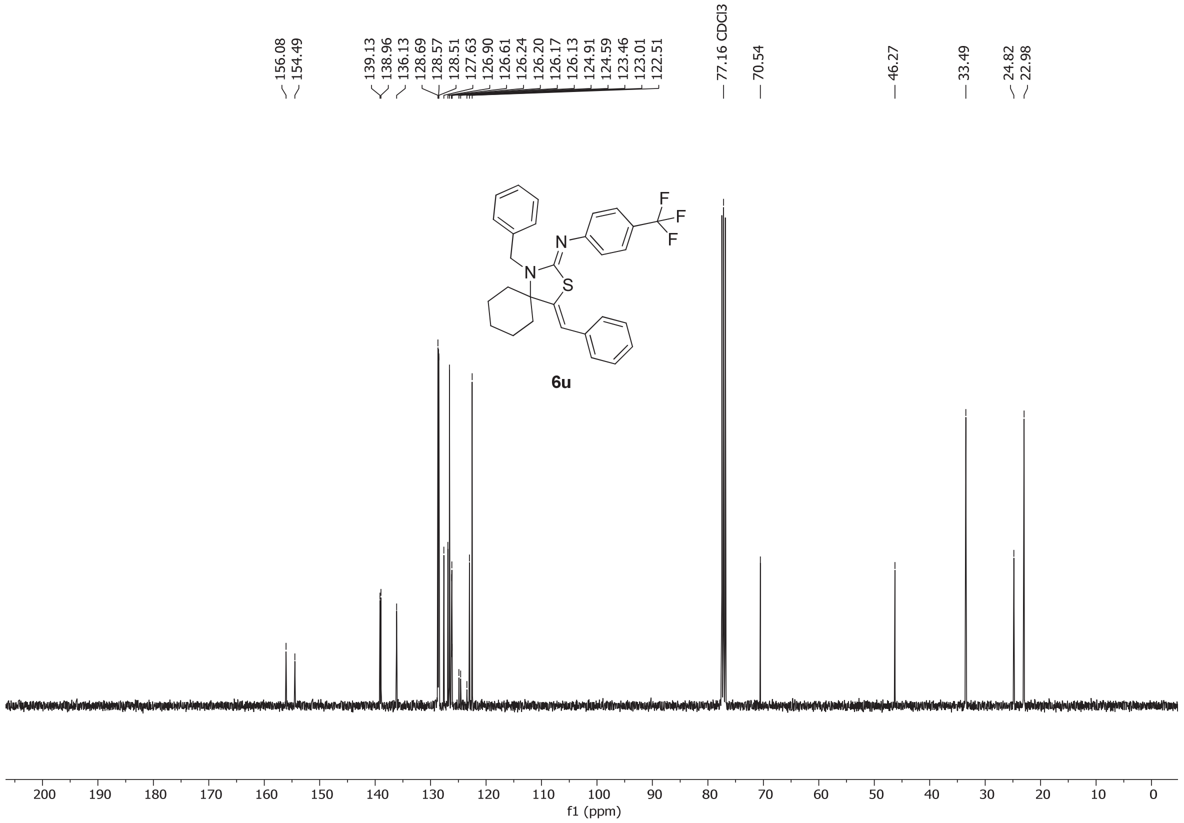

156.08 154.49
139.13 138.96 136.13 128.69 128.57 128.51 127.63 126.90 126.61 126.24 126.20 126.17 126.13 124.91 124.59 123.46 123.01 122.51
77.16 CDCl3
70.54
46.27
33.49
24.82 22.98
F F F N N S
6u
200 190 180 170 160 150 140 130 120 110 100 90 80 70 60 50 40 30 20 10 0
f1 (ppm)

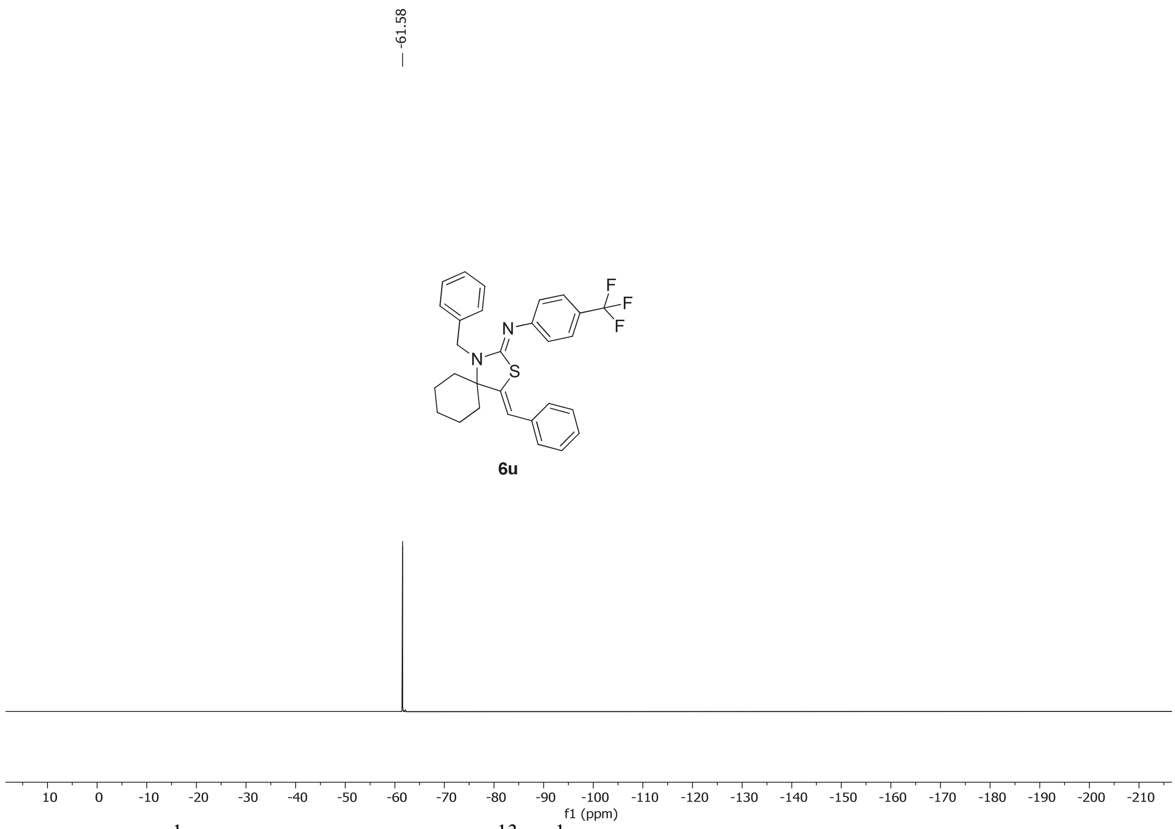

Figure S21: ^{1}H-NMR (400 MHz, up),^{13}C{^{1}H}-NMR (101 MHz, middle), and ^{19}F{^{1}H}-NMR (376 MHz, bottom) spectra for **6u** in $CDCl_3$.

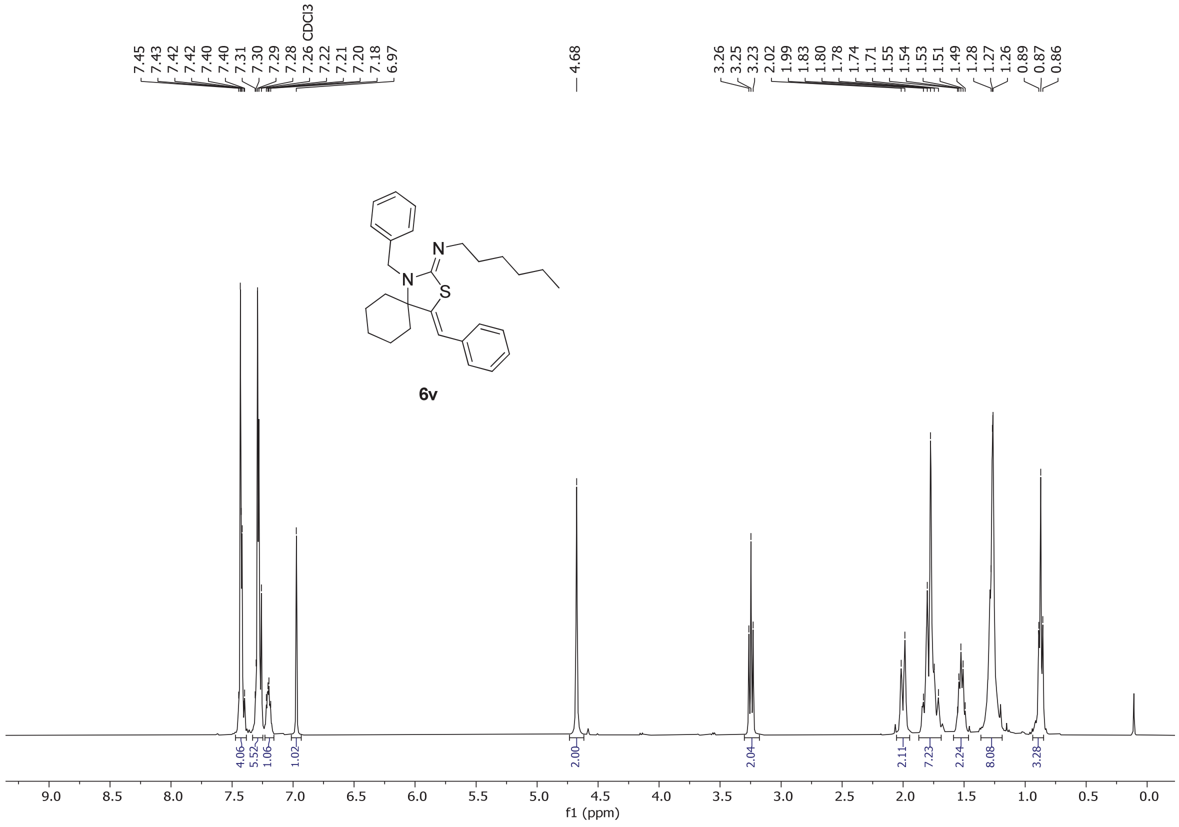

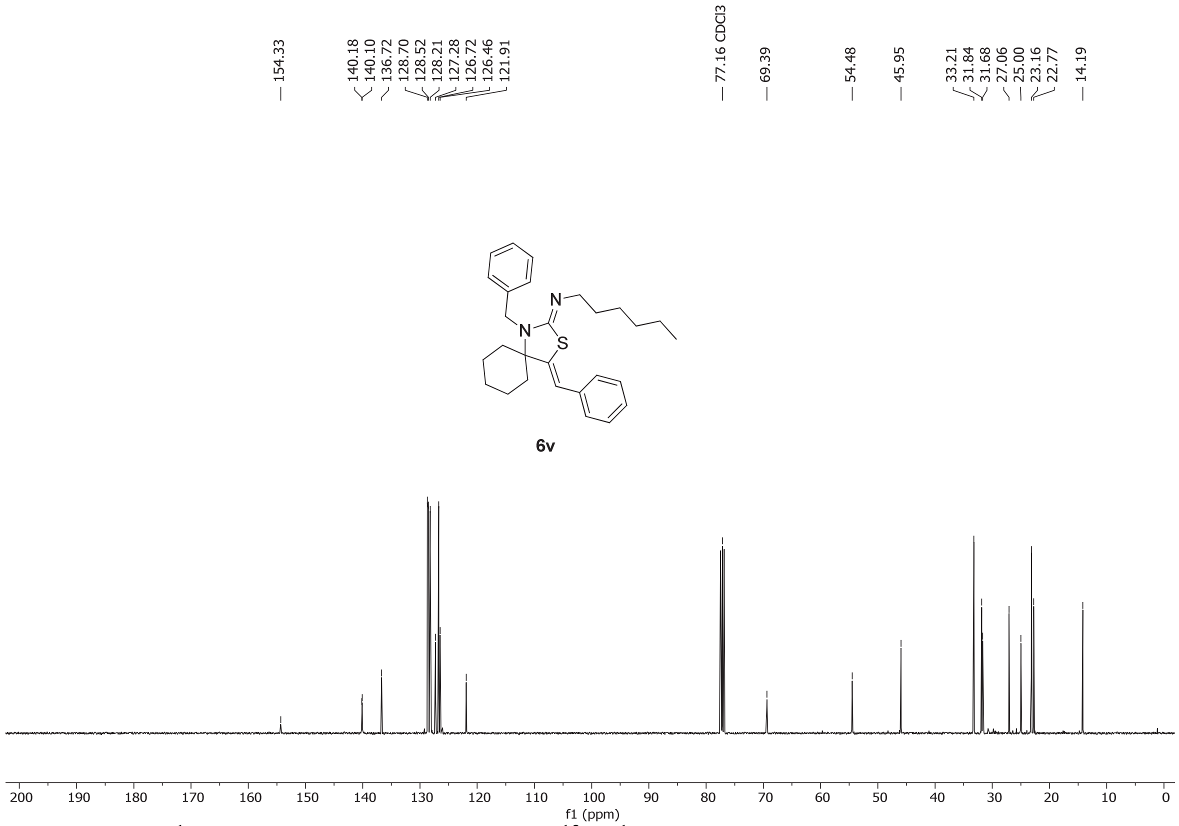

Figure S22: ^{1}H-NMR (400 MHz, up) and ^{13}C{^{1}H}-NMR (101 MHz, bottom) spectra for **6v** in $CDCl_3$.

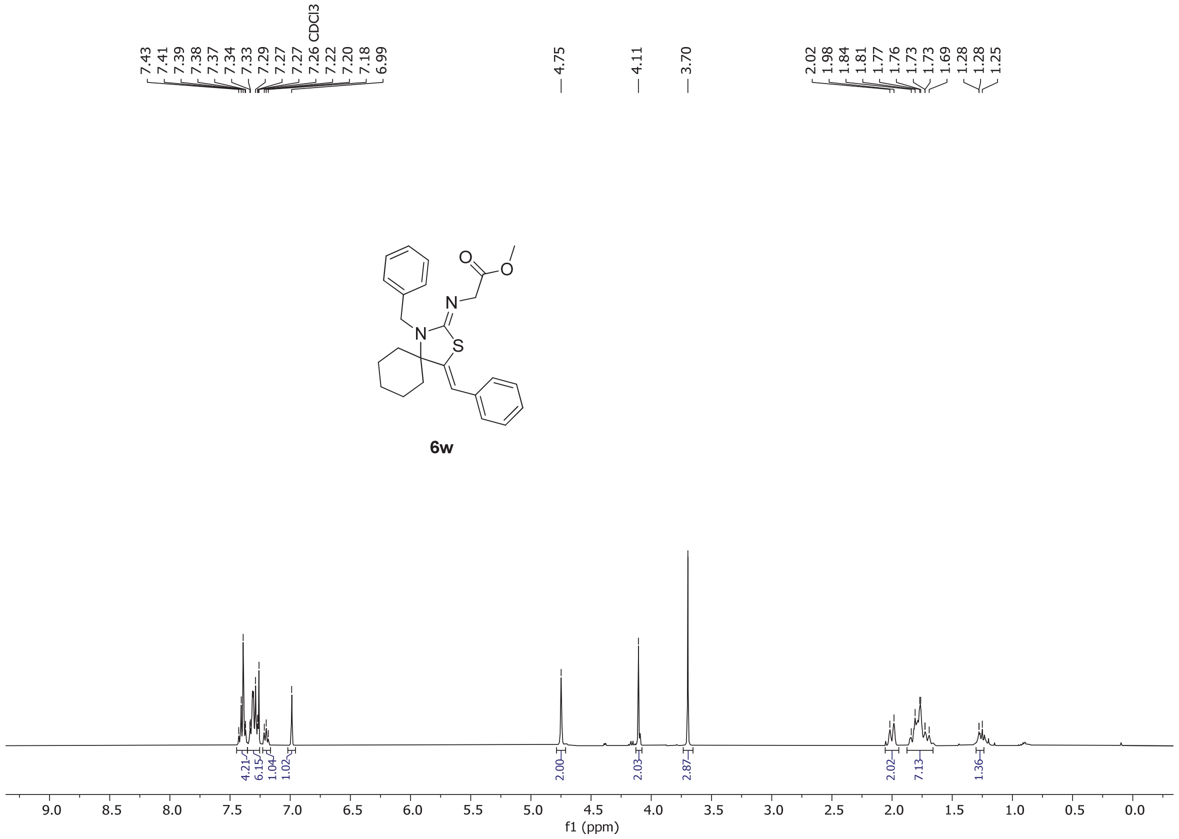

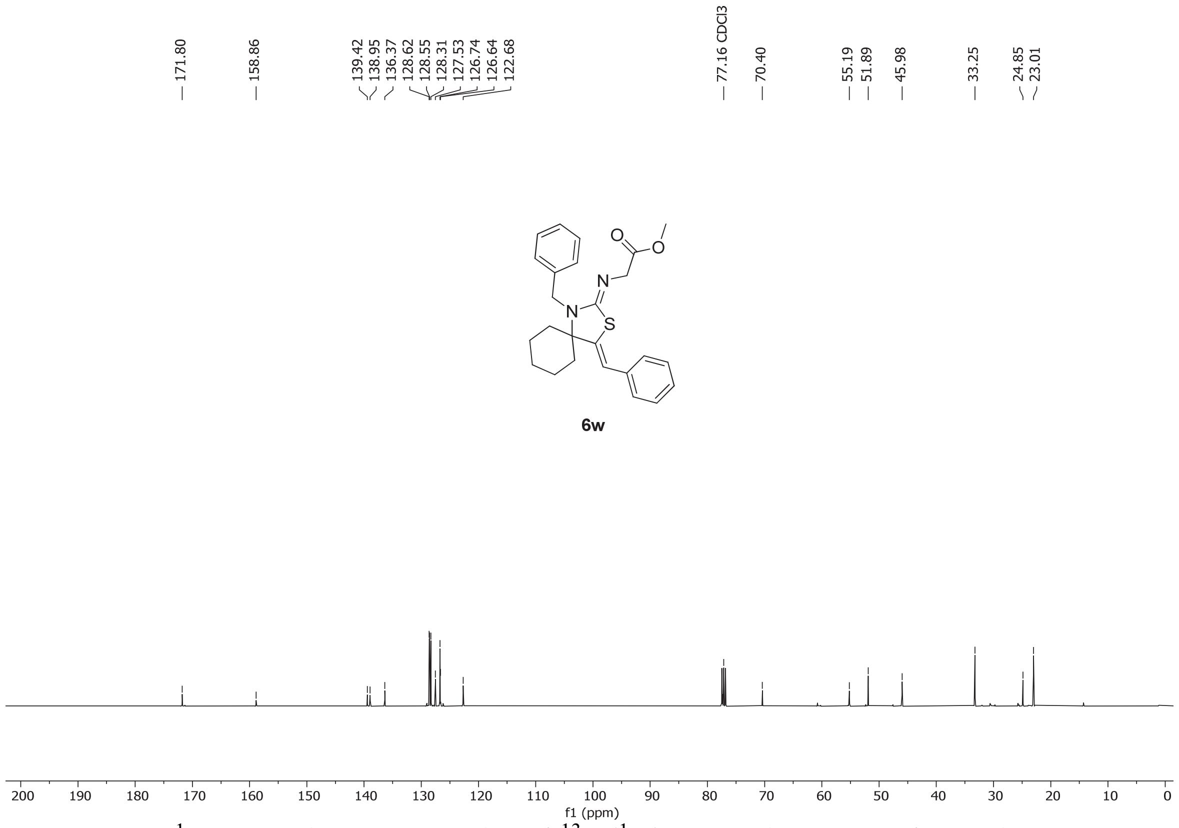

Figure S23: ^{1}H-NMR (400 MHz, up) and ^{13}C{^{1}H}-NMR (101 MHz, bottom) spectra for **6w** in $CDCl_3$.

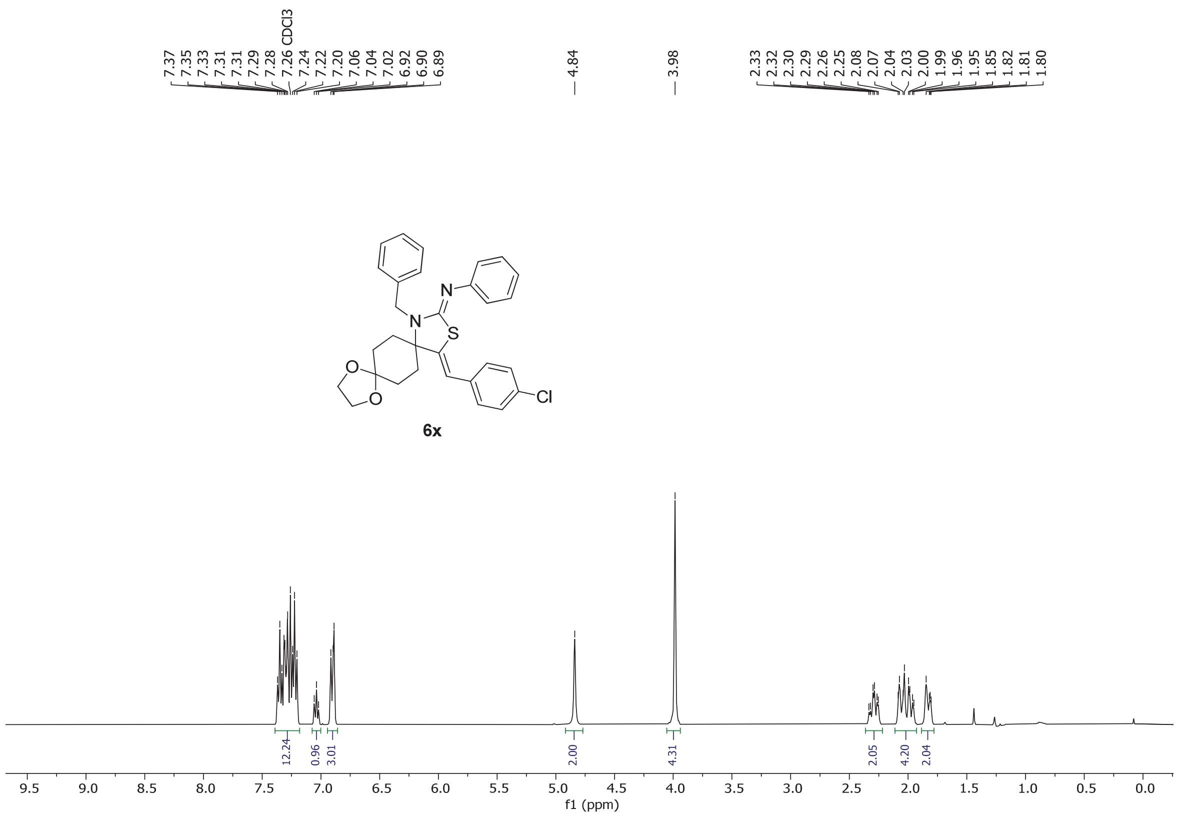

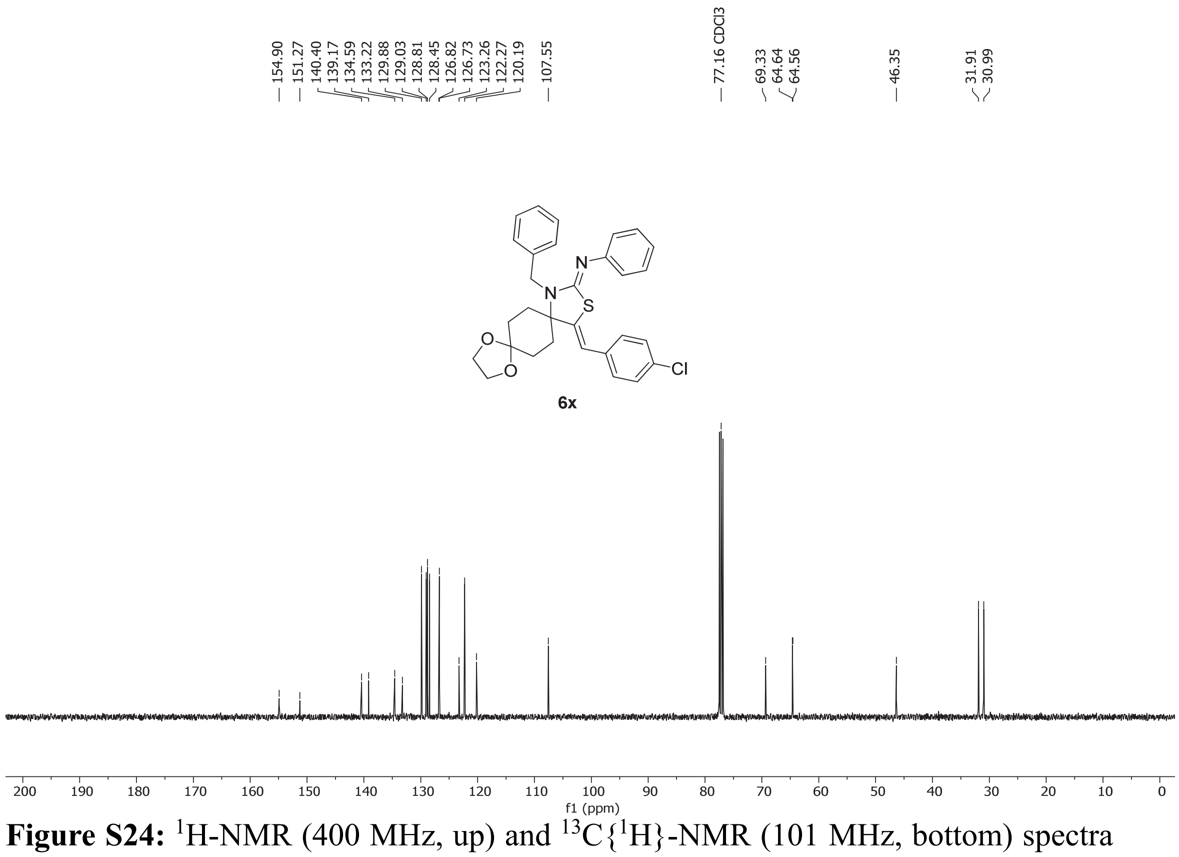

Figure S24: ^{1}H-NMR (400 MHz, up) and $^{13}C\{^{1}H\}$-NMR (101 MHz, bottom) spectra for **6x** in $CDCl_3$.

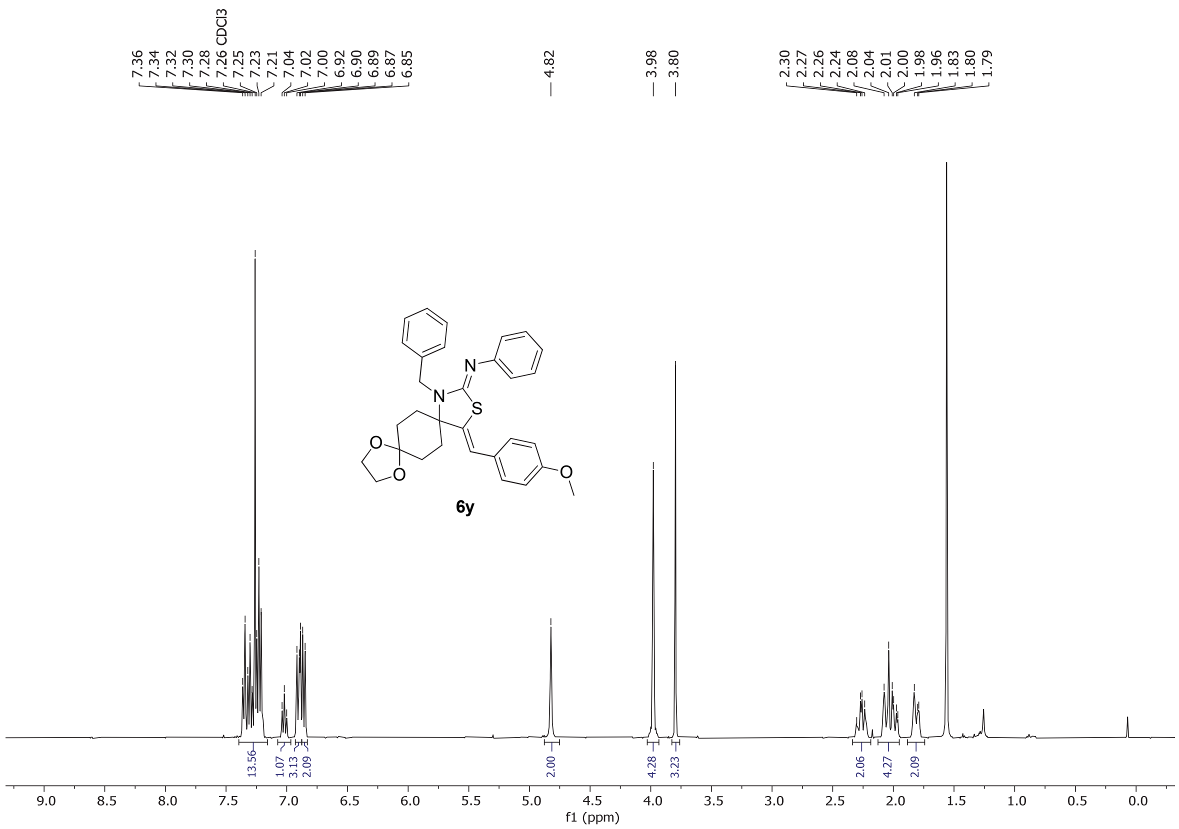

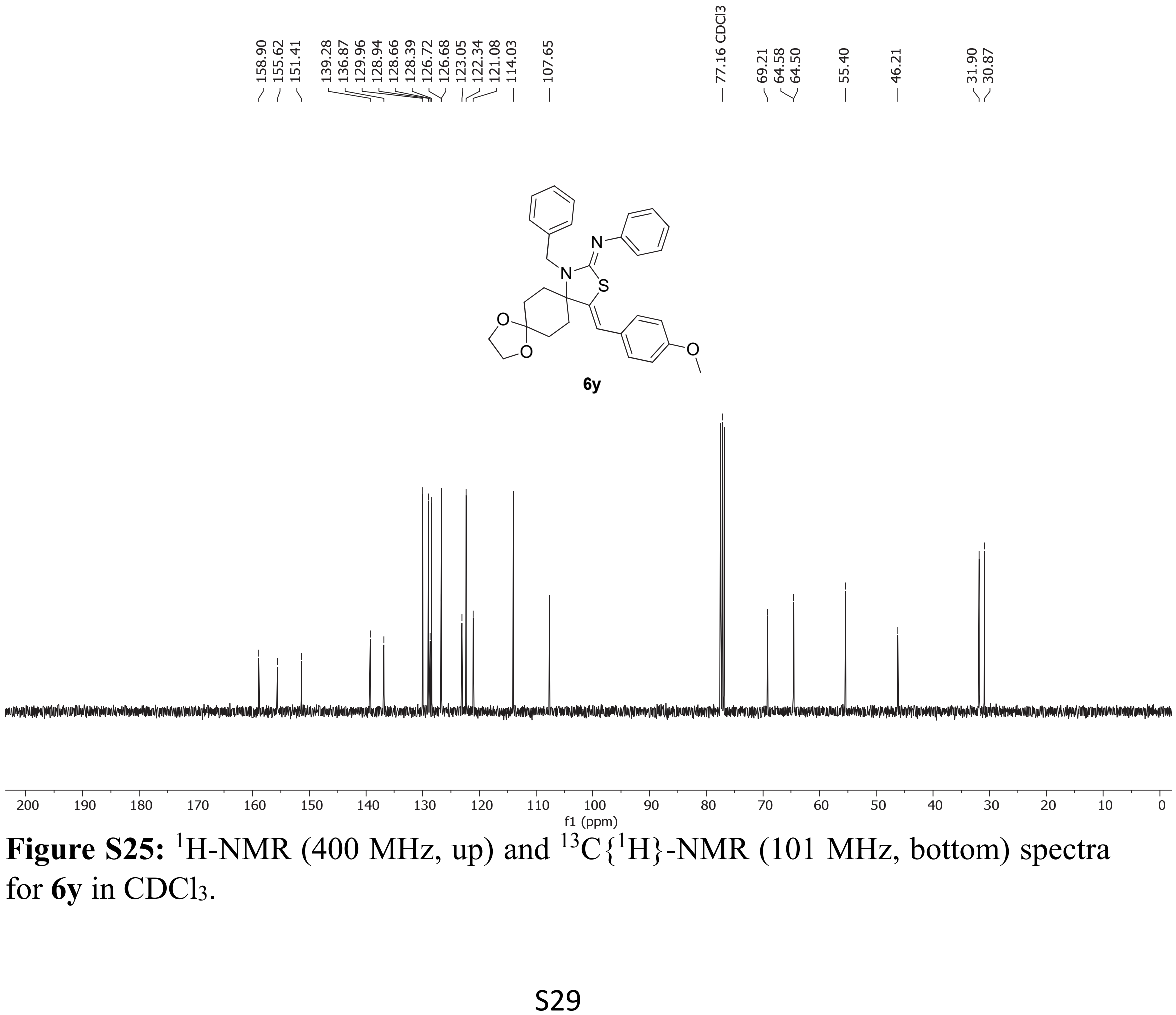

Figure S25: ^{1}H-NMR (400 MHz, up) and ^{13}C{^{1}H}-NMR (101 MHz, bottom) spectra for **6y** in $CDCl_3$.

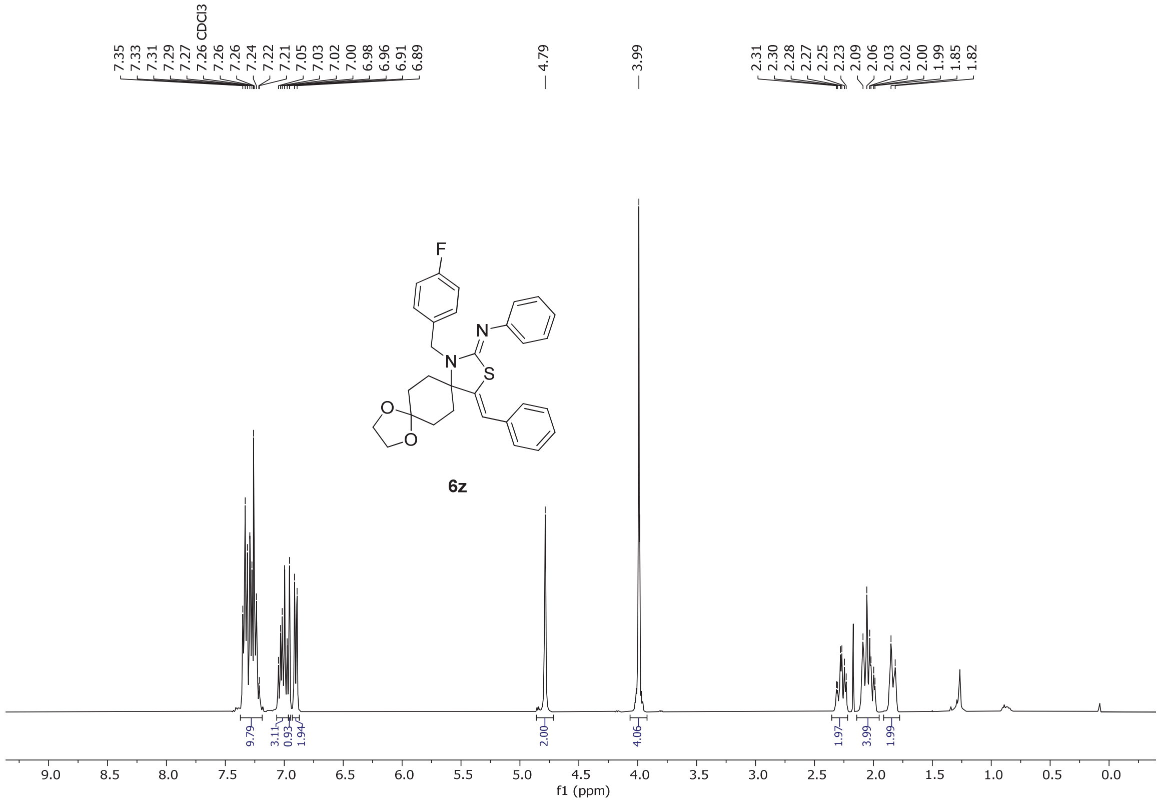
7.35 7.33 7.31 7.29 7.27 7.26 CDCl3 7.26 7.24 7.22 7.21 7.05 7.03 7.02 7.00 6.98 6.96 6.91 6.89
4.79
3.99
2.31 2.30 2.28 2.27 2.25 2.23 2.09 2.06 2.03 2.02 2.00 1.99 1.85 1.82
F
N
N
S
O
O
6z
9.79 3.11 0.93 1.94
2.00
4.06
1.97 3.99 1.99
f1 (ppm)

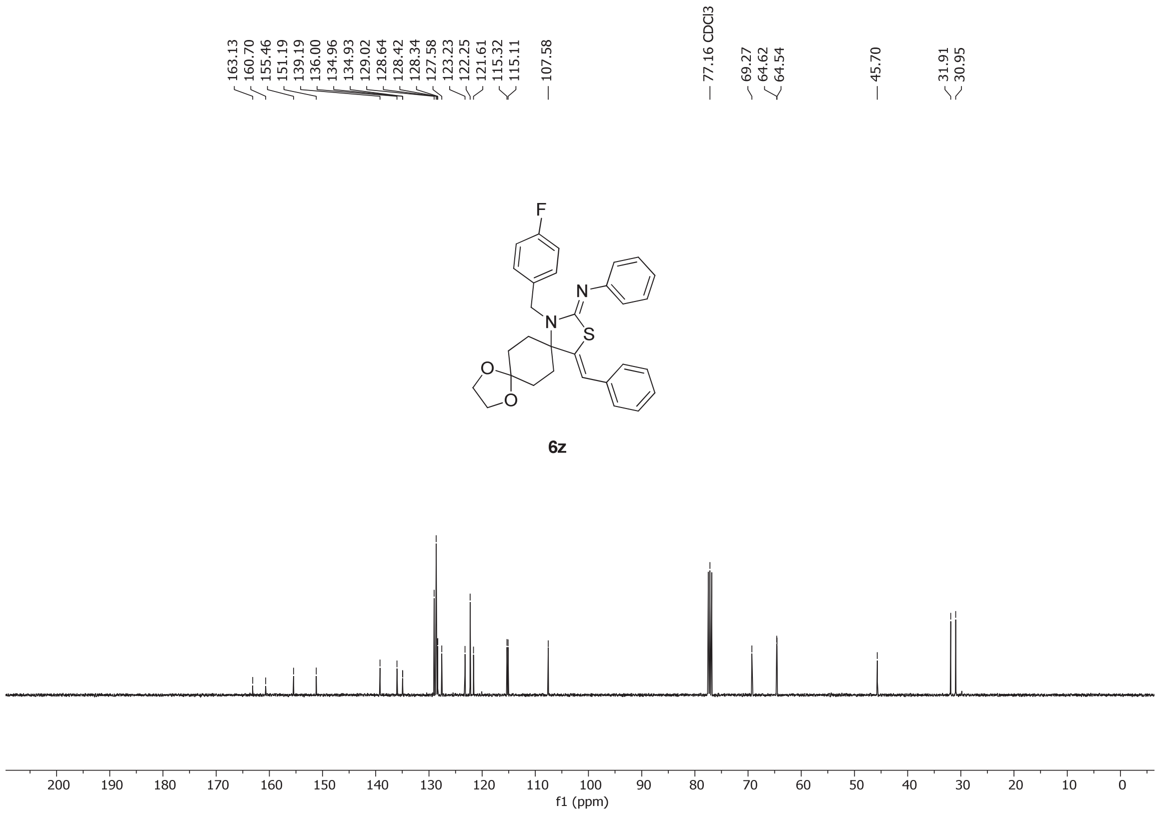
163.13 160.70 155.46 151.19 139.19 136.00 134.96 134.93 129.02 128.64 128.42 128.34 127.58 123.23 122.25 121.61 115.32 115.11
107.58
77.16 CDCl3
69.27 64.62 64.54
45.70
31.91 30.95
F
N
N
S
O
O
6z
f1 (ppm)

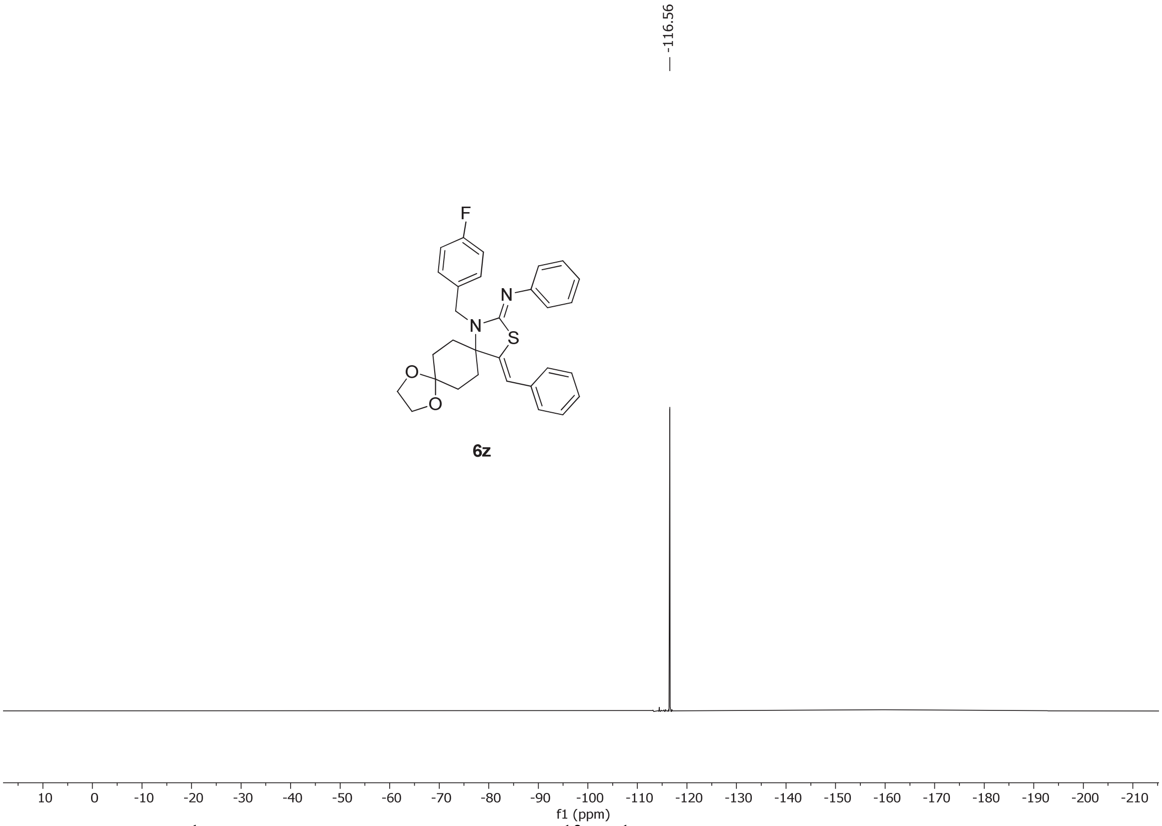

Figure S26: ^{1}H-NMR (400 MHz, up), ^{13}C{^{1}H}-NMR (101 MHz, middle), and ^{19}F{^{1}H}-NMR (376 MHz, bottom) for **6z** in $CDCl_3$.

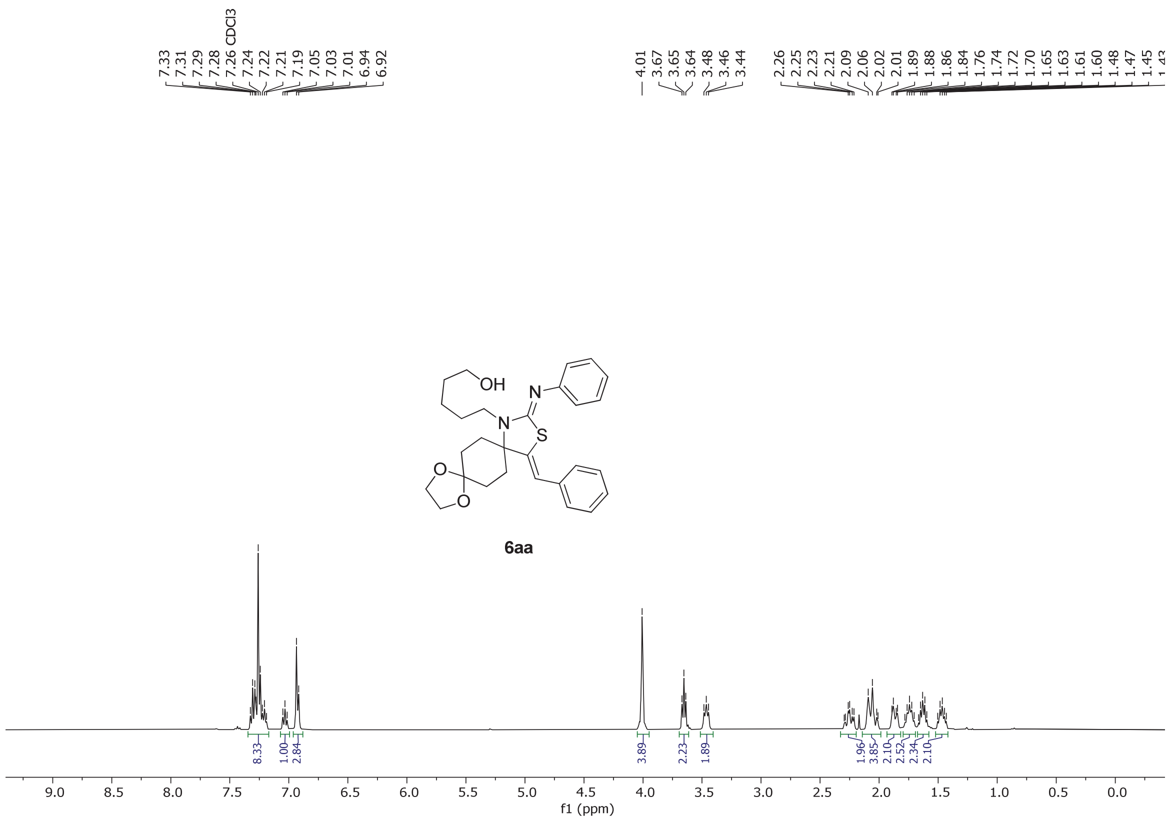

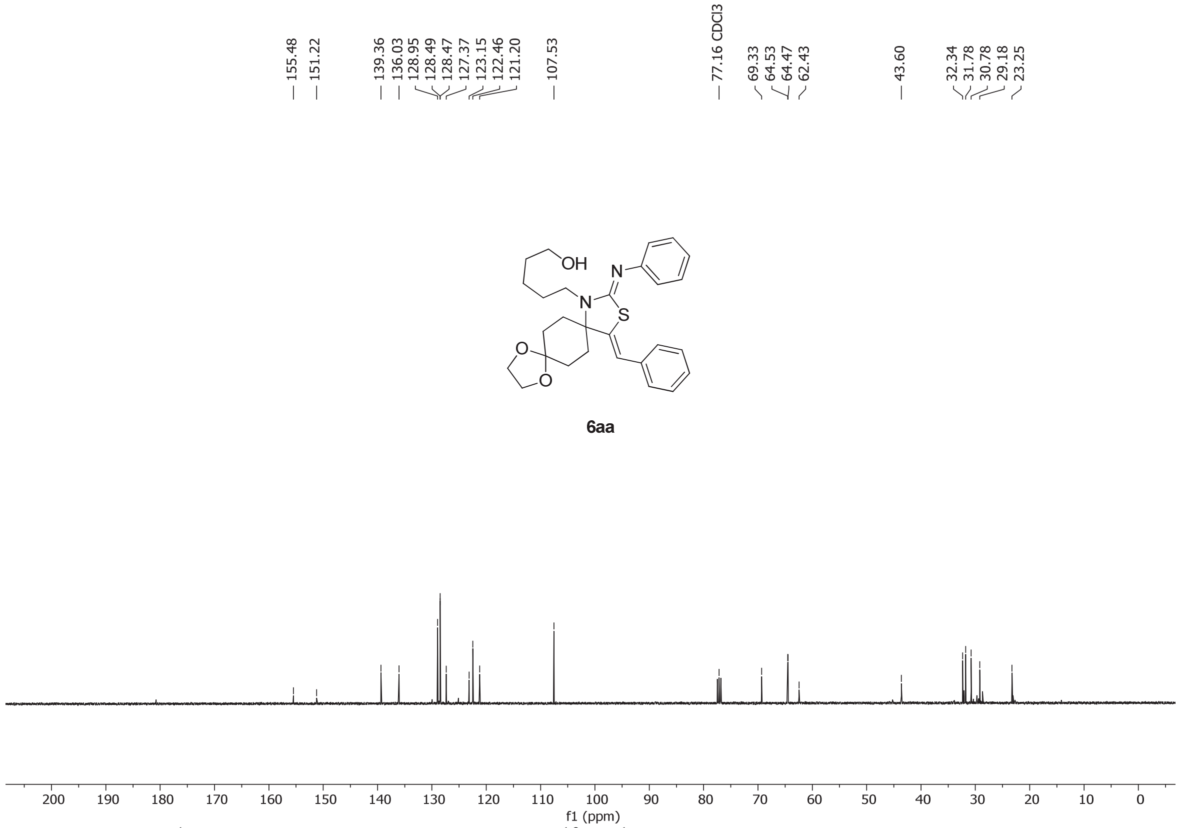

Figure S27: ^{1}H-NMR (400 MHz, up) and ^{13}C{^{1}H}-NMR (101 MHz, bottom) spectra for **6aa** in $CDCl_3$.

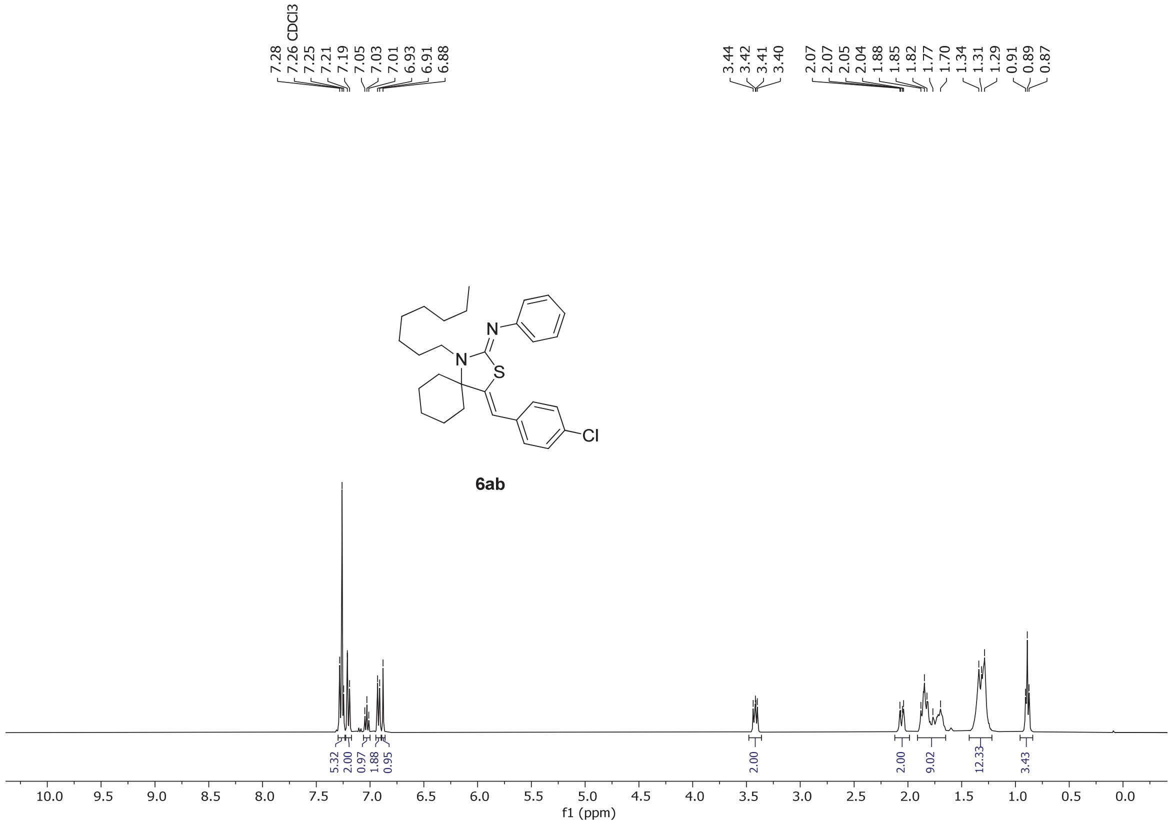

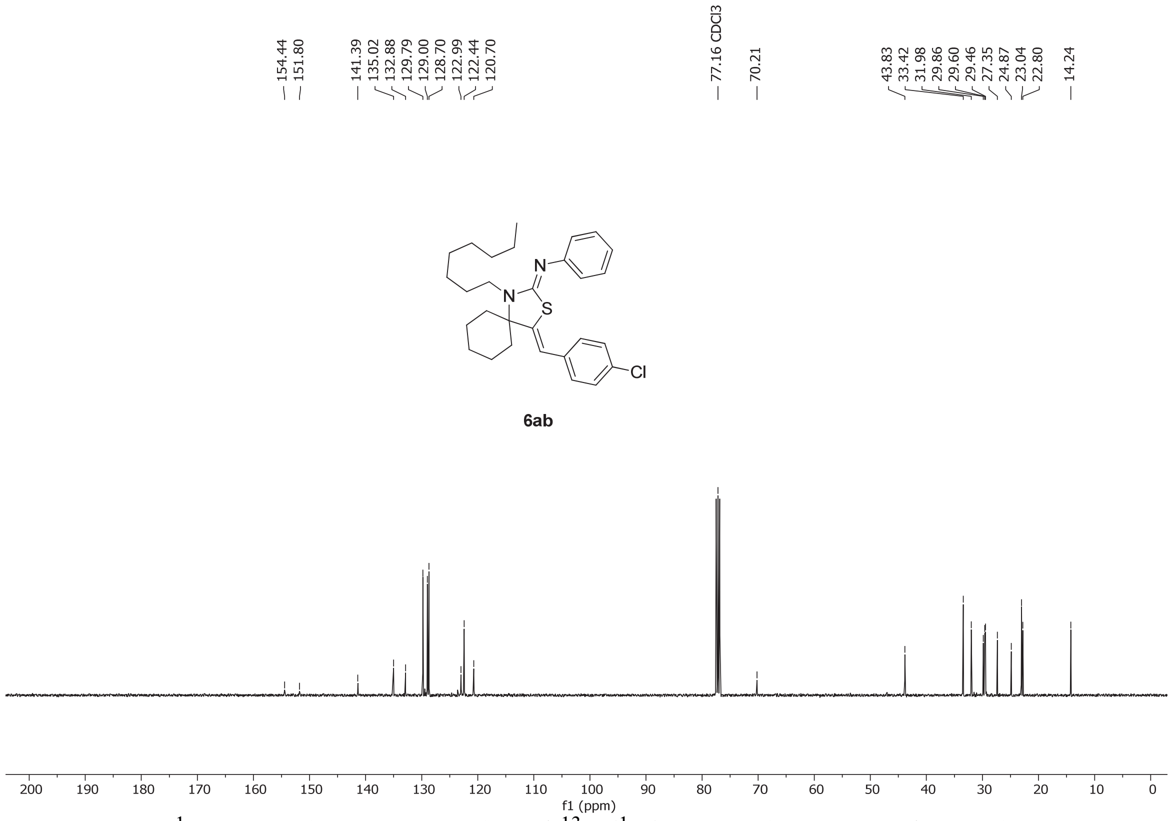

Figure S28: ^{1}H-NMR (400 MHz, up) and ^{13}C{^{1}H}-NMR (101 MHz, bottom) spectra for **6ab** in $CDCl_3$.

2. Supporting schemes for DFT calculations concerning the intramolecular *vs* intermolecular proton transfer

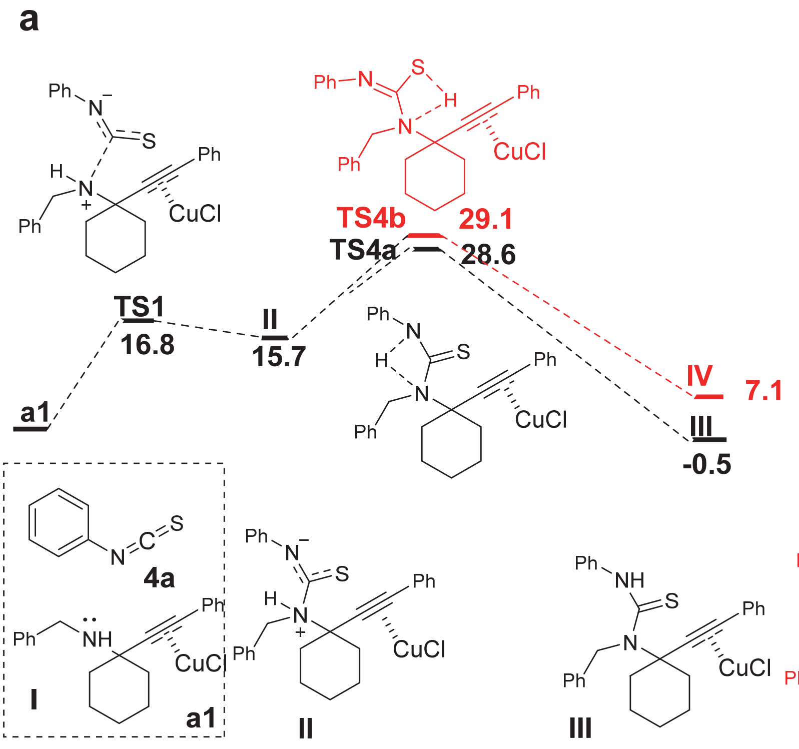

Scheme S1. Intramolecular proton transfer pathways from **II** to produce thiourea **III** or its imine thiol tautomer **IV**.

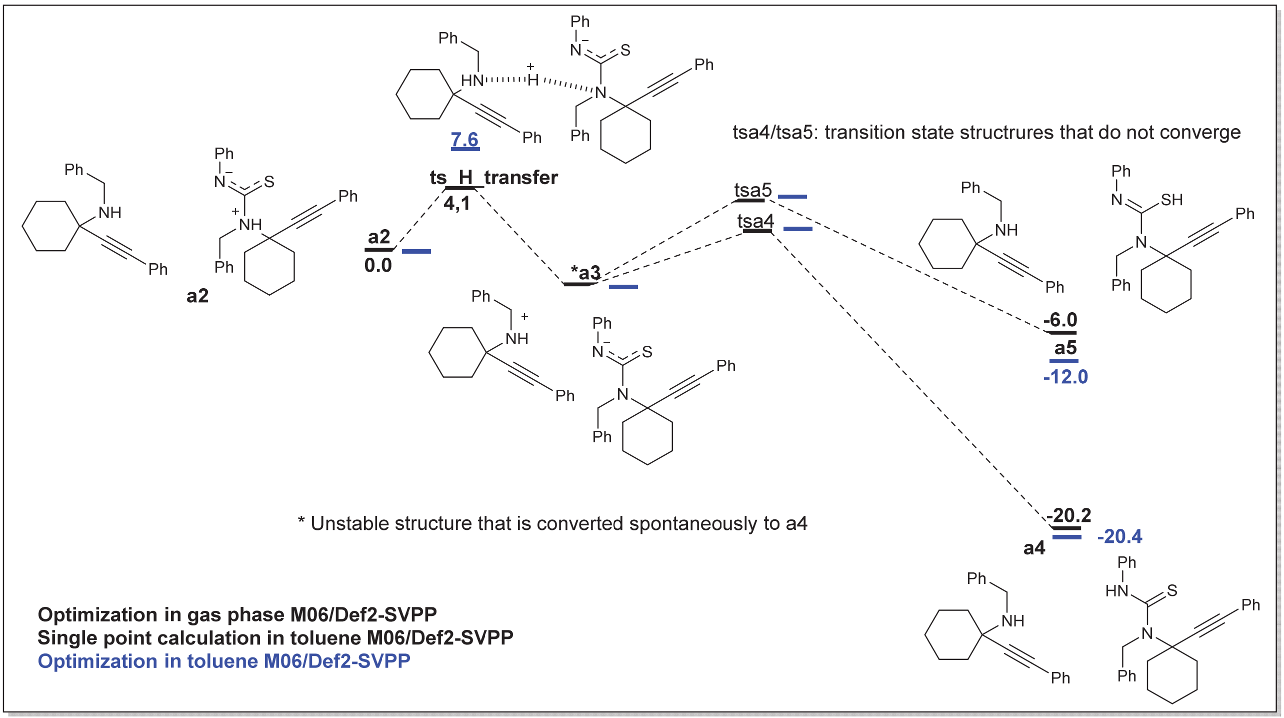

Scheme S2. Intermolecular proton transfer pathways from **a2** to produce thiourea **a4**.

3. Cartesian coordinates of the structures of Scheme 5

Cartesian coordinates of the structures in Scheme 5 describing plausible mechanistic pathways by DFT calculations for the Cu(I)-catalyzed cyclization of propargylamine thiourea III and its tautomer IV (optimization in toluene M06/Def2-SVPP)

I

SCF Energy: -3689.889246
ZPE-corrected Energy: -3689.399521
ΔU: -3689.367223
ΔH: -3689.366278
ΔG: -3689.468627
Num. Imaginary Frequencies:0

C	0.64599800	-1.42186700	-0.16672000
C	0.69198900	-2.12934300	1.20253100
N	-0.61203500	-0.70772800	-0.30075300
C	-0.26929700	-3.30689800	1.29806800
H	1.72847000	-2.49975300	1.34570000
H	0.50211100	-1.38076500	1.99605900
C	-0.04688600	-4.29760700	0.16282600
H	-0.13657600	-3.80076500	2.27886900
H	-1.32214300	-2.95223700	1.29052900
C	-0.10545900	-3.60634500	-1.19359900
H	0.95400900	-4.76291000	0.28336100
H	-0.78391900	-5.12115900	0.21554200
C	0.90435200	-2.47017600	-1.27032600
H	0.08632900	-4.33015700	-2.00754600
H	-1.12867600	-3.20873400	-1.36746400
H	1.93067500	-2.87630100	-1.14097300
H	0.88633100	-1.98266800	-2.26377600
C	1.70789600	-0.38096900	-0.16874300
C	-2.01876900	0.33803700	2.44191200
C	-0.96296600	-0.14222800	-1.58199400
H	-1.41493100	-0.87774400	-2.28907500
C	-1.87565000	1.05206300	-1.45819500
H	-0.01937400	0.19344300	-2.06210100
N	-2.87435900	-0.23493700	1.84114500
S	-0.85053800	1.02745600	3.25725800
C	2.41955500	0.63461700	-0.15312900
C	3.03061000	1.93162400	-0.05719700
C	2.44225900	2.91149200	0.75964300
C	3.01938700	4.17302800	0.85498100
C	4.18454800	4.46734600	0.14583500
C	4.77666800	3.49533200	-0.66073800
C	4.20586700	2.23152700	-0.76369500
H	1.53365800	2.66551000	1.32200900
H	2.55692300	4.93452500	1.49280600
H	4.63710200	5.46219600	0.22548300
H	5.69395800	3.72349100	-1.21453900
H	4.66806600	1.46134000	-1.39357700
C	-1.60027600	2.05282600	-0.51855600
C	-2.40562000	3.18473300	-0.42984800
C	-3.49932500	3.33625500	-1.28574000
C	-3.78217500	2.34473400	-2.22245600
C	-2.97630900	1.20827800	-2.30267100
H	-0.74264200	1.92365300	0.15496500
H	-2.17593800	3.96008500	0.31130400
H	-4.13135400	4.22993200	-1.21965100

H	-4.64321800	2.45070500	-2.89351400
H	-3.20839400	0.42334900	-3.03528800
H	-1.39012800	-1.17162600	0.16467800
C	-3.94706600	-0.43431700	1.00059200
C	-4.83598100	0.61502100	0.73091100
C	-5.89760200	0.40660300	-0.14154900
C	-6.08174800	-0.83539800	-0.75030200
C	-5.19850200	-1.87950800	-0.47601500
C	-4.13591500	-1.68752100	0.40152400
H	-4.67040300	1.59089900	1.20014500
H	-6.58620300	1.23208200	-0.35592400
H	-6.91928900	-0.99125800	-1.43916700
H	-5.34103100	-2.85957900	-0.94473600
H	-3.44701500	-2.50680800	0.63941400
Cu	3.60932000	-1.03009600	-0.24686500
Cl	5.05230600	-2.56733000	-0.31867100

TS1
SCF Energy: -3689.864853
ZPE-corrected Energy: -3689.374576
ΔU: -3689.343649
ΔH: -3689.342704
ΔG: -3689.441826
Num. Imaginary Frequencies:1
Imaginary Frequency: -222.5794

C	-0.44826100	-0.37900400	-1.38103000
C	0.07299900	0.70865300	-2.34502600
N	0.57099300	-0.68663100	-0.34021800
C	1.23043800	0.23824900	-3.21747600
H	-0.78196000	0.98145000	-2.99637600
H	0.33347300	1.61720900	-1.77207600
C	0.85732400	-1.01316900	-4.00110200
H	1.50932400	1.05790100	-3.90582400
H	2.12823600	0.05387800	-2.59271100
C	0.36204900	-2.11515300	-3.07433400
H	0.05147100	-0.76371100	-4.72335900
H	1.71621400	-1.36751900	-4.60089900
C	-0.79804400	-1.63717100	-2.21080400
H	0.03370900	-2.99758000	-3.65396500
H	1.19759700	-2.47646000	-2.43724200
H	-1.65100400	-1.36320000	-2.86557700
H	-1.17209700	-2.44755500	-1.55600600
C	-1.65958600	0.15370900	-0.72412000
C	1.51781400	0.92182600	0.38587600
C	0.13979800	-1.52519000	0.78834200
H	-0.63401800	-2.25593800	0.47571000
C	1.31361100	-2.24597600	1.38618200
H	-0.33022100	-0.84562100	1.52855500
N	2.61402900	0.79291500	-0.18310500
S	0.53011800	1.71020200	1.40851200
C	-2.63109800	0.79815600	-0.30230100
C	-3.54033700	1.79963600	0.17958400
C	-3.05027700	3.09361100	0.42378500
C	-3.91055000	4.08048300	0.89144200
C	-5.25587500	3.78746800	1.11999700
C	-5.74498800	2.50269600	0.88221500
C	-4.89295800	1.50864700	0.41404700
H	-1.98839700	3.30441300	0.24964100

H	-3.52732700	5.08894400	1.08263800
H	-5.92981100	4.56845700	1.48984500
H	-6.80022500	2.27239900	1.06483400
H	-5.26915500	0.49464000	0.22743200
C	2.04561600	-1.69117800	2.44025600
C	3.15982400	-2.35483700	2.94998900
C	3.55392700	-3.57871500	2.41032000
C	2.82797600	-4.14122300	1.36088500
C	1.71280300	-3.47730400	0.85432700
H	1.73541800	-0.72569100	2.86050800
H	3.72585800	-1.91154100	3.77719800
H	4.43001000	-4.10005300	2.81308500
H	3.12709800	-5.10797700	0.93982300
H	1.12811700	-3.92700700	0.03919500
H	1.40157600	-1.09076700	-0.78434300
C	3.74375800	1.60879900	-0.11051000
C	3.82574200	2.74994000	0.70261000
C	4.99020900	3.51304200	0.71377600
C	6.08214500	3.15689300	-0.07823800
C	6.00432600	2.02244000	-0.88751200
C	4.84572700	1.25407100	-0.90323800
H	2.96856000	3.03127400	1.32453400
H	5.04476400	4.40235200	1.35305800
H	6.99501900	3.76332100	-0.06407200
H	6.85718100	1.73283400	-1.51259100
H	4.76779900	0.35723500	-1.52916000
Cu	-3.00937700	-1.18463100	-0.03071800
Cl	-3.68805800	-3.14911900	0.33990500

II

SCF Energy: -3689.868444

ZPE-corrected Energy: -3689.376460

ΔU: -3689.345499

ΔH: -3689.344555

ΔG: -3689.443668

Num. Imaginary Frequencies:0

C	-0.55853100	-1.11783700	0.30752000
C	-0.29581200	-2.02555300	-0.91131800
N	0.68061500	-0.28807600	0.63305800
C	0.71225600	-3.13893300	-0.65315200
H	-1.27773200	-2.48284700	-1.15515000
H	-0.01550300	-1.40302100	-1.77936900
C	0.32065400	-3.97553500	0.55657300
H	0.76839900	-3.77009200	-1.55901700
H	1.72608600	-2.71062200	-0.51825700
C	0.14220000	-3.09716400	1.78676200
H	-0.63902500	-4.49438900	0.34944500
H	1.07315700	-4.76268600	0.74677000
C	-0.89094500	-2.00880000	1.52768600
H	-0.19150800	-3.69535000	2.65452300
H	1.11997100	-2.66241500	2.09188500
H	-1.86140900	-2.49551500	1.29904600
H	-1.07157200	-1.39048600	2.42661000
C	-1.68603300	-0.20468100	0.03646200
C	1.49221200	0.27769500	-0.61166300
C	0.47456300	0.79474400	1.65416700
H	-0.28422000	0.45037900	2.37607700
C	1.76809800	1.10737900	2.34379100

H	0.06991400	1.65711000	1.09645700
N	2.60173900	-0.35939300	-0.62499400
S	0.71929300	1.48056900	-1.49048600
C	-2.57402000	0.64880900	-0.10171700
C	-3.35622300	1.83585800	-0.30351300
C	-2.69917400	3.03097200	-0.64028600
C	-3.44024000	4.19148400	-0.83461900
C	-4.82927100	4.16938100	-0.70166500
C	-5.48437400	2.98223500	-0.37250800
C	-4.75346000	1.81683600	-0.17194200
H	-1.60781000	3.02492700	-0.75322300
H	-2.92834100	5.12380400	-1.09760200
H	-5.40797100	5.08667600	-0.85846600
H	-6.57492000	2.96412600	-0.27128000
H	-5.26196100	0.87904500	0.08688300
C	2.65184200	2.06089200	1.82750700
C	3.87415000	2.29322200	2.45319100
C	4.22355800	1.57599900	3.59745700
C	3.34822500	0.62415500	4.11805300
C	2.12548200	0.39209200	3.49179500
H	2.37725700	2.61383200	0.91955800
H	4.56230000	3.04000000	2.04165500
H	5.18631000	1.76111300	4.08737700
H	3.61712500	0.06214100	5.01952900
H	1.43045500	-0.35295200	3.90551400
H	1.37968400	-0.94576400	1.01405300
C	3.67847100	-0.14981400	-1.48076400
C	3.93290300	1.03772100	-2.18981100
C	5.07263600	1.14914400	-2.98285500
C	5.96939300	0.08791700	-3.10154800
C	5.72590600	-1.09450800	-2.40027600
C	4.60102200	-1.20608100	-1.59216200
H	3.22730200	1.87036200	-2.11431100
H	5.25878800	2.08443600	-3.52493200
H	6.85813000	0.18234400	-3.73633700
H	6.42519200	-1.93556000	-2.47875100
H	4.40584700	-2.12150500	-1.02021200
Cu	-3.41663400	-1.20652700	-0.24598200
Cl	-4.41409800	-3.06414300	-0.36650800

III
SCF Energy: -3689.8952953
ZPE-corrected Energy: -3689.402952
ΔU: -3689.372160
ΔH: -3689.371162
ΔG: -3689.469444
Num. Imaginary Frequencies:0

C	-0.29384800	-0.62300000	1.28860400
C	-0.32945200	-2.17234500	1.39238100
N	0.83314400	-0.14613400	0.44033500
C	0.77736800	-2.80374300	2.22835400
H	-1.30547500	-2.40333600	1.86665800
H	-0.37538600	-2.64222300	0.39249800
C	0.88409000	-2.18157400	3.61130300
H	0.57511700	-3.88948000	2.29924700
H	1.74817800	-2.70996400	1.70923000
C	0.98689000	-0.66920200	3.49818200
H	-0.01743400	-2.43589300	4.20821100

H	1.75055800	-2.60124500	4.15645200
C	-0.20689500	-0.10457200	2.74406900
H	1.03384700	-0.20022200	4.49896800
H	1.92686600	-0.38871300	2.97751600
H	-1.14122500	-0.43195100	3.24349600
H	-0.21184500	0.99458800	2.76110500
C	-1.58470900	-0.18163700	0.69702500
C	1.15091200	1.21486300	0.38414800
C	1.07493400	-0.89335900	-0.80720600
H	1.40573000	-0.16486800	-1.56920400
C	2.05753000	-2.03457100	-0.76288800
H	0.10510900	-1.26481200	-1.19983400
N	2.41251200	1.42107600	-0.10407200
S	0.14737800	2.47719400	0.80052400
C	3.04458000	2.63251300	-0.46844600
C	4.38147700	2.80635000	-0.09752700
C	5.07643600	3.95021600	-0.48288900
C	4.43918300	4.93491400	-1.23443500
C	3.10660200	4.75802300	-1.60858500
C	2.40856300	3.61299900	-1.23695700
H	4.87668700	2.03467800	0.50532800
H	6.12390900	4.07281400	-0.18468400
H	4.98099600	5.83923900	-1.53334000
H	2.60044900	5.52123000	-2.21089700
H	1.36902900	3.47272900	-1.54583600
C	-2.70837800	0.23234300	0.37330500
C	-3.89211200	1.02527800	0.18387700
C	-3.90674800	2.33928100	0.68024300
C	-5.03866200	3.12909800	0.51306100
C	-6.15730100	2.62062000	-0.14900300
C	-6.14473400	1.31790600	-0.64791700
C	-5.01767200	0.51962500	-0.48380500
H	-3.01555500	2.72494200	1.18901400
H	-5.04840600	4.15381600	0.90106500
H	-7.04727500	3.24683700	-0.27894200
H	-7.02155700	0.91973400	-1.17022600
H	-4.99878200	-0.50572000	-0.87470100
C	3.26254600	-1.96399300	-0.05541500
C	4.17192200	-3.01917900	-0.08509700
C	3.88374600	-4.16852600	-0.82006500
C	2.68055700	-4.25469000	-1.51854000
C	1.77507000	-3.19498900	-1.49067200
H	3.48188300	-1.09703500	0.58414500
H	5.10782500	-2.94735200	0.48098900
H	4.59493300	-5.00221800	-0.83994400
H	2.43830800	-5.15971300	-2.08735000
H	0.81766800	-3.27145600	-2.02574400
H	3.03104300	0.61054400	-0.10491000
Cu	-2.27139800	-1.30424700	-0.87616600
Cl	-2.13433200	-2.83431600	-2.33955000

IV

SCF Energy: -3689.8792679

ZPE-corrected Energy: -3689.390480

ΔU: -3689.359339

ΔH: -3689.358395

ΔG: -3689.457280

Num. Imaginary Frequencies:0

C	0.23944900	-1.36904300	0.13475400
C	0.35007600	-1.87436400	1.60732000
N	-1.02077800	-0.61881900	-0.13102900
C	-0.28903100	-3.21804300	1.95735600
H	1.43712900	-2.00113200	1.78966300
H	0.01898900	-1.06810000	2.28986600
C	0.02019800	-4.27961900	0.91405000
H	0.09791300	-3.52224500	2.94818200
H	-1.38608600	-3.14080100	2.08500900
C	-0.37461700	-3.78295200	-0.46839200
H	1.10999100	-4.49534600	0.91934300
H	-0.49572500	-5.22510400	1.16390400
C	0.44896400	-2.55903900	-0.83161200
H	-0.20995000	-4.56648000	-1.23102200
H	-1.45992500	-3.54693600	-0.49652900
H	1.52243600	-2.84326900	-0.79170700
H	0.25434800	-2.22590700	-1.86709100
C	1.35135300	-0.39543800	-0.04079000
C	-1.02302500	0.48783400	-0.98737000
C	-2.21707000	-0.80655100	0.68601300
H	-2.27798600	-0.08974100	1.53114400
C	-3.49754000	-0.75898600	-0.10932500
H	-2.14193700	-1.80553600	1.14048600
N	-1.45663000	1.66003300	-0.74207600
S	-0.46544000	0.13374500	-2.65053400
C	-1.74122100	2.15747400	0.53013100
C	-0.81559900	2.09557700	1.58538800
C	-1.12808000	2.64263900	2.82736400
C	-2.36155500	3.25859500	3.04051900
C	-3.27493300	3.34324300	1.98867400
C	-2.96389900	2.81217300	0.74050700
H	0.15705200	1.61445100	1.42128800
H	-0.39420800	2.58692800	3.64019600
H	-2.60473800	3.68422500	4.02061600
H	-4.24127800	3.83907200	2.13983200
H	-3.66833700	2.87790000	-0.09674100
C	2.13681800	0.54892300	-0.21664000
C	2.75748900	1.80938300	-0.51804900
C	1.94714500	2.89306400	-0.89998300
C	2.53160000	4.11837100	-1.20174800
C	3.91622300	4.27504600	-1.12861000
C	4.72310000	3.20179100	-0.75034000
C	4.14904800	1.97287500	-0.44459900
H	0.85830700	2.76312600	-0.95653500
H	1.89745900	4.96150900	-1.49723500
H	4.37065800	5.24297700	-1.36839300
H	5.81030800	3.32273700	-0.69252500
H	4.77814600	1.12435900	-0.14631900
C	-3.60438900	-1.38882700	-1.35417000
C	-4.81319700	-1.39242700	-2.04575800
C	-5.93540000	-0.76828400	-1.50063000
C	-5.83892800	-0.13904400	-0.26104400
C	-4.62575600	-0.13235400	0.42656300
H	-2.72326600	-1.87479400	-1.79304400
H	-4.87947900	-1.88792200	-3.02136400
H	-6.88634600	-0.77014000	-2.04590300
H	-6.71419300	0.35765300	0.17384300
H	-4.54808100	0.37333400	1.39832200
H	-0.48015200	1.43815300	-3.03203900
Cu	3.19435200	-1.14367400	0.22711600
Cl	4.38519200	-2.81746700	0.71948200

TS2

SCF Energy: -3689.865778

ZPE-corrected Energy: -3689.377553

ΔU: -3689.347253

ΔH: -3689.346309

ΔG: -3689.441062

Num. Imaginary Frequencies:1

Imaginary Frequency: -255.2304

C	-0.92764800	0.63462300	-0.28012400
C	-1.41519700	1.72283500	-1.26991400
N	-1.43926100	-0.72265500	-0.66701200
C	-2.79911200	2.30845500	-1.00841900
H	-0.70665000	2.57133400	-1.18347600
H	-1.29270400	1.35008000	-2.30506000
C	-2.92535600	2.76701100	0.43610400
H	-2.94322600	3.15699100	-1.70367700
H	-3.60977800	1.58725400	-1.22973700
C	-2.70749300	1.58585100	1.36897900
H	-2.16385400	3.54941400	0.63995300
H	-3.91738400	3.22376400	0.60988200
C	-1.31548600	0.99775800	1.17949900
H	-2.80725900	1.89798000	2.42582800
H	-3.49366000	0.82518200	1.19621800
H	-0.58875500	1.76592600	1.51796900
H	-1.14628000	0.10888100	1.81748100
C	0.57116600	0.59260800	-0.34194600
C	-0.61635600	-1.76593400	-0.34832900
C	-2.68748700	-0.94029000	-1.38150700
H	-2.55588800	-1.84847400	-2.00301700
C	-3.94358600	-1.07800500	-0.55172100
H	-2.81955200	-0.13603600	-2.12357700
N	0.53036000	-1.48560200	0.14253500
S	-1.27022800	-3.41159000	-0.61767300
C	1.61670100	-2.28557900	0.46793000
C	2.12264900	-3.27000400	-0.39894600
C	3.26080300	-3.99392900	-0.05108200
C	3.91709300	-3.74340900	1.15270900
C	3.42958600	-2.75401000	2.00912100
C	2.29161500	-2.03033400	1.67180300
H	1.64481800	-3.43524600	-1.37241400
H	3.64676700	-4.75490200	-0.73968300
H	4.81574900	-4.31050500	1.41986600
H	3.94668800	-2.54054900	2.95168200
H	1.90771600	-1.23724000	2.32472100
C	1.74291700	1.04577000	-0.46574500
Cu	1.16114700	2.86258000	-0.01555100
C	3.14770700	0.69152900	-0.53732100
C	4.10535700	1.38193100	0.22024400
C	5.44073500	0.99088900	0.19166900
C	5.84377700	-0.07414100	-0.61364000
C	4.90194400	-0.74640900	-1.39336200
C	3.56352400	-0.36958900	-1.35664000
H	3.78790600	2.22712300	0.84447300
H	6.17616900	1.53067600	0.79921900
H	6.89700800	-0.37626000	-0.64037000
H	5.21198200	-1.58134000	-2.03309700
H	2.81883100	-0.89816700	-1.96341700

Cl	0.58076600	4.80489700	0.62876700
C	-3.93730100	-1.63703700	0.72925000
C	-5.12041100	-1.76345400	1.45409400
C	-6.32949800	-1.33645500	0.90657900
C	-6.34760000	-0.78432100	-0.37301600
C	-5.16178300	-0.65782800	-1.09403300
H	-2.99210500	-1.96218800	1.18040200
H	-5.09563500	-2.19683200	2.46054300
H	-7.25878400	-1.43146100	1.47972700
H	-7.29135700	-0.44131500	-0.81229100
H	-5.18189300	-0.21400300	-2.09872300
H	-0.17675300	-4.01612500	-0.07678500

TS3
SCF Energy: -3689.881283
ZPE-corrected Energy: -3689.389836
ΔU: -3689.359614
ΔH: -3689.358669
ΔG: -3689.456288
Num. Imaginary Frequencies:1
Imaginary Frequency: -235.0005

C	0.34433000	-1.10836200	0.01762900
C	0.75267600	-2.22205600	1.01089800
N	-0.95093700	-0.45613300	0.42318800
C	0.04153700	-3.56361500	0.85452200
H	1.82274000	-2.43219000	0.81357200
H	0.72830100	-1.82909600	2.04517000
C	0.16667900	-4.06145400	-0.57671300
H	0.50498500	-4.27668900	1.56228300
H	-1.03230600	-3.51390500	1.12678200
C	-0.44799600	-3.04012000	-1.51975000
H	1.24054600	-4.19810500	-0.82420800
H	-0.32296300	-5.04654300	-0.68966300
C	0.25930800	-1.69663100	-1.41220400
H	-0.38424500	-3.38317700	-2.56955200
H	-1.52813600	-2.93794900	-1.29230400
H	1.30611900	-1.83654500	-1.75465900
H	-0.18826500	-0.94338400	-2.08624500
C	1.39964000	-0.03407700	0.04791900
C	-1.20543300	0.78923300	-0.06666800
C	-1.84547700	-1.00801000	1.43371100
H	-2.13214100	-0.18125100	2.11454900
C	-3.09619600	-1.70076500	0.93593200
H	-1.27962400	-1.69592400	2.07719200
N	-2.42718800	1.32156600	0.20181900
S	-0.03618600	1.61739100	-0.94948900
C	-2.89174700	2.61662200	-0.15037200
H	-3.17069900	0.67478700	0.46813700
C	-4.11673400	2.71932600	-0.81297500
C	-4.63732300	3.97283200	-1.12636300
C	-3.93230400	5.12618300	-0.78866600
C	-2.71101000	5.01961900	-0.12245900
C	-2.19083600	3.77140500	0.20632700
H	-4.66007200	1.80576400	-1.08678100
H	-5.59910300	4.04512000	-1.64641500
H	-4.33702000	6.11264600	-1.04077300
H	-2.15707300	5.92307500	0.15635900
H	-1.24281600	3.68773400	0.74732900

C	2.54574100	0.40749700	0.38455800
Cu	3.36801600	-1.28190500	-0.17047000
C	3.29342500	1.64104100	0.51631100
C	4.66172800	1.69463000	0.20419800
C	5.36784600	2.88802700	0.31430700
C	4.72699300	4.04294000	0.76337000
C	3.37260200	3.99722400	1.09561200
C	2.65785100	2.81012000	0.97022000
H	5.16864200	0.78056100	-0.13218700
H	6.43277900	2.91390300	0.05643700
H	5.28650600	4.98042700	0.86103300
H	2.86551700	4.90032600	1.45533800
H	1.59228500	2.77369400	1.22591500
Cl	4.23303100	-3.09423200	-0.88512500
C	-3.53188800	-1.64628400	-0.39095400
C	-4.71691500	-2.27735600	-0.77528200
C	-5.47782800	-2.97137800	0.16106500
C	-5.04840700	-3.03433000	1.48767600
C	-3.86855500	-2.40355800	1.86906000
H	-2.93650100	-1.11728400	-1.14661800
H	-5.04079300	-2.22817300	-1.82104300
H	-6.40597700	-3.46867400	-0.14202200
H	-5.63735700	-3.58326700	2.23118700
H	-3.53171500	-2.45716100	2.91325400

V

SCF Energy: -3689.906270

ZPE-corrected Energy: -3689.413233

ΔU: -3689.383051

ΔH: -3689.382106

ΔG: -3689.477997

Num. Imaginary Frequencies:0

C	-0.53694000	-1.09834500	-0.39928700
C	-1.21101500	-1.76840000	-1.61473100
N	0.93598400	-0.90132500	-0.63177700
C	-1.18620700	-3.29578700	-1.69305700
H	-2.27695800	-1.46702000	-1.54522000
H	-0.82393900	-1.30041300	-2.54192000
C	-1.52695400	-3.94140800	-0.35607500
H	-1.92351600	-3.60555600	-2.45767600
H	-0.21474100	-3.68112300	-2.06538500
C	-0.62898200	-3.42084300	0.75940200
H	-2.58153800	-3.70319700	-0.10280000
H	-1.45719800	-5.04260800	-0.43497200
C	-0.78338100	-1.91113800	0.88532500
H	-0.91248900	-3.88384000	1.72345800
H	0.42771400	-3.71938500	0.58960800
H	-1.84010300	-1.70868200	1.15900500
H	-0.17321800	-1.47959100	1.70460600
C	-1.10097700	0.31688200	-0.20570400
C	1.40434300	0.33882400	-0.49768100
C	1.82720000	-1.97457900	-1.06491700
H	2.20427400	-1.73997100	-2.08191700
C	2.99417400	-2.25673300	-0.14477900
H	1.21500700	-2.87976300	-1.17651600
N	2.70769600	0.61971900	-0.65807400
S	0.24561800	1.54342700	-0.11299100
C	3.33858000	1.87624000	-0.43607200

H	3.34417500	-0.18168500	-0.66161100
C	4.43554200	1.90835400	0.42806600
C	5.10470300	3.10851800	0.64848500
C	4.67351500	4.27539000	0.01797800
C	3.58134100	4.23462100	-0.84735700
C	2.91602900	3.03472300	-1.08910500
H	4.75324800	0.98412700	0.92927000
H	5.96472800	3.13295300	1.32671300
H	5.19545600	5.22150300	0.19816000
H	3.24740400	5.14597300	-1.35530600
H	2.07825900	2.99593300	-1.79346700
C	-2.37637500	0.67141500	-0.02089300
Cu	-3.74555600	-0.66553200	0.08692700
C	-2.74639700	2.07936900	0.18418500
C	-3.37794600	2.48261900	1.37357800
C	-3.71902800	3.81379500	1.58897100
C	-3.46469300	4.77567600	0.60903000
C	-2.86315500	4.38822500	-0.58690100
C	-2.50328500	3.05752600	-0.79594700
H	-3.60271100	1.72277000	2.13332400
H	-4.20173000	4.10411900	2.53006200
H	-3.74733800	5.82200400	0.77373800
H	-2.67379900	5.13066800	-1.37196800
H	-2.04345200	2.75720300	-1.74671000
Cl	-5.08208100	-2.32995500	0.23150000
C	2.88696700	-2.14620700	1.24603400
C	3.98335400	-2.41702500	2.06182100
C	5.20018300	-2.80317000	1.49954700
C	5.31669700	-2.91423600	0.11530200
C	4.22097800	-2.63749500	-0.70101600
H	1.93870900	-1.83029800	1.69803600
H	3.88475600	-2.32511000	3.14917500
H	6.06139900	-3.01411200	2.14328500
H	6.26963000	-3.21251000	-0.33588400
H	4.31919500	-2.71717400	-1.79183800

VI

SCF Energy: -3689.902344

ZPE-corrected Energy: -3689.411812

ΔU: -3689.381787

ΔH: -3689.380843

ΔG: -3689.474329

Num. Imaginary Frequencies:0

C	-0.96311400	0.48988600	-0.45440300
C	-1.19954100	1.48142400	-1.60221500
N	-1.56160600	-0.86010800	-0.76657600
C	-2.51282200	2.25961700	-1.57120600
H	-0.37449600	2.22029100	-1.52264100
H	-1.04423600	0.96089600	-2.56885900
C	-2.74716700	2.89336600	-0.20818600
H	-2.47328900	3.03503900	-2.35960600
H	-3.38168900	1.61646100	-1.82500300
C	-2.78326600	1.81699600	0.86680200
H	-1.92538300	3.60872100	0.00881200
H	-3.68935800	3.47319000	-0.21072200
C	-1.47013300	1.04683200	0.89036500
H	-2.93960900	2.26915900	1.86468700
H	-3.64671300	1.14253700	0.69705200

H	-0.67500400	1.75347000	1.21227200
H	-1.47599300	0.22624400	1.63674400
C	0.52442400	0.11861800	-0.31963100
C	-0.65249700	-1.81678400	-0.61384200
C	-2.89294800	-1.10654300	-1.29725900
H	-2.85026700	-2.05917100	-1.86241600
C	-4.00863900	-1.15445700	-0.28457600
H	-3.10496700	-0.33723800	-2.05936800
N	0.56176400	-1.34113600	-0.34616900
S	-1.09947600	-3.50311900	-0.71876500
C	1.67785500	-2.14571500	0.03241100
C	2.30279600	-2.95841800	-0.91537600
C	3.37653200	-3.75900100	-0.53234400
C	3.83242300	-3.72911500	0.78471000
C	3.21062700	-2.90406200	1.72249600
C	2.12697100	-2.11343700	1.35145000
H	1.95341200	-2.94282600	-1.95526000
H	3.87043700	-4.39696800	-1.27335000
H	4.68458700	-4.34967700	1.08275800
H	3.57373200	-2.87485400	2.75558700
H	1.63151700	-1.45233400	2.07184500
C	1.54276500	0.97124000	-0.14517400
Cu	1.18654100	2.84486600	0.08171500
C	2.96587900	0.61725900	-0.07173100
C	3.71968800	0.94262900	1.07135500
C	5.07688600	0.64958700	1.14795600
C	5.73353700	0.05536700	0.06793800
C	5.01016400	-0.23992200	-1.08664200
C	3.64511600	0.03315900	-1.15426200
H	3.21420000	1.44044700	1.90980800
H	5.63550700	0.90456100	2.05695200
H	6.80727000	-0.15955500	0.12077900
H	5.51524600	-0.69118800	-1.95003200
H	3.08342600	-0.19928500	-2.06925100
Cl	0.60448500	4.88361900	0.37235200
C	-3.82083700	-1.68449500	0.99434600
C	-4.87264900	-1.71243000	1.90745100
C	-6.12735600	-1.22165400	1.54770200
C	-6.32660700	-0.70747000	0.26757200
C	-5.27114100	-0.67315500	-0.64135700
H	-2.83608000	-2.06521100	1.29438000
H	-4.70930300	-2.11931100	2.91184000
H	-6.95223200	-1.23852800	2.26890700
H	-7.30869000	-0.31740500	-0.02280000
H	-5.42642200	-0.24914300	-1.64251300
H	0.16729700	-3.95828500	-0.50694700

4. X-ray crystallographic data for 6t

Single Crystal X-ray Crystallography (SC-XRD):

Colorless crystals of compound **6t** grown from slow evaporation of solution of **6t** in chloroform were mounted on Mitigen loops from silicon oil at RT. Data were collected at 100K using a Bruker D8-Venture SC-XRD diffractometer equipped with a four-circle kappa goniometer, a Photon-III area detector and an *IμS* Diamond Cu/Kα source (λ = 1,54178 Å). Data collection, integration and scaling were handled by the APEX4 software suite (Bruker AXS, 2021) and a numerical absorption correction based on crystal faces was applied. Data were collected to a resolution of 0.80 Å. Structure solution and model refinement were performed using the Olex2-1.5, software suite, and hydrogens were added using the riding model.[1-3] The maximum residual e^- density of 1.17 e. $Å^{-3}$ after final model refinement was located at a distance of 1.23 Å away from the bromine atom and resulted in a C-level alert (PLAT094); it is attributed to absorption correction artifacts and has no chemical meaning. All other data collection and final model refinement parameters are given in the table below.

Compound	**6t**
Colour, habit	Colourless, Rod
Size/mm	0.27 x 0.02 x 0.02
Empirical formula	$C_{28}H_{27}BrN_2S$
FW	503.48
Crystal system	Monoclinic
Space group	*C2/c*
a/Å	43.202(2)
b/Å	6.1514(4)
c/Å	19.0226(9)
a/°	90
b/°	114.0520(10)
g/°	90
$V/Å^3$	4616.4(4)
Z	8
μ/mm^{-1}	3.405
T/K	100
$\theta_{min/max}$	4.483/ 74.519
Completeness to θ_{max} (%)	99.8 to 74.519
Reflections Total/ Independent	48432/4403
Parameters/restraints/constraints	289/0/0
R_{int}	0.0393
Final *R*1, *wR*2	0.0276/0.0675
Goof	1.062
Largest peak, hole / e.$Å^{-3}$	1.2, -0.4
$\rho_{calc}/g\ cm^{-3}$	1.449
CCDC Reference	2322776

5. References

1 O. V. Dolomanov, L. J. Bourhis, R. J. Gildea, J. A. K. Howard, H. Puschmann, *J. Appl. Crystallogr.* **2009**, *42*, 339–341.

2 G. M. Sheldrick, *Acta Crystallogr. A* **2008**, *64*, 112–122.

3 G. M. Sheldrick, *Acta Crystallogr. Sect. Found. Adv.* **2015**, *71*, 3–8.
